# The *Seminavis robusta* genome provides insights into the evolutionary adaptations of benthic diatoms

**DOI:** 10.1038/s41467-020-17191-8

**Published:** 2020-07-03

**Authors:** Cristina Maria Osuna-Cruz, Gust Bilcke, Emmelien Vancaester, Sam De Decker, Atle M. Bones, Per Winge, Nicole Poulsen, Petra Bulankova, Bram Verhelst, Sien Audoor, Darja Belisova, Aikaterini Pargana, Monia Russo, Frederike Stock, Emilio Cirri, Tore Brembu, Georg Pohnert, Gwenael Piganeau, Maria Immacolata Ferrante, Thomas Mock, Lieven Sterck, Koen Sabbe, Lieven De Veylder, Wim Vyverman, Klaas Vandepoele

**Affiliations:** 1 Department of Plant Biotechnology and Bioinformatics, Ghent University, Technologiepark 71, 9052 Ghent, Belgium; 2 VIB Center for Plant Systems Biology, Technologiepark 71, 9052 Ghent, Belgium; 3 Bioinformatics Institute Ghent, Ghent University, Technologiepark 71, 9052 Ghent, Belgium; 4 Protistology and Aquatic Ecology, Department of Biology, Ghent University, 9000 Ghent, Belgium; 5 Department of Applied Mathematics, Computer Science and Statistics, Ghent University, 9000 Ghent, Belgium; 6 Cell Molecular Biology and Genomics Group, Department of Biology, Norwegian University of Science and Technology, 7491 Trondheim, Norway; 7 B CUBE Center for Molecular Bioengineering, Technical University of Dresden, Tatzberg 41, 01307 Dresden, Germany; 8 Integrative Marine Ecology, Stazione Zoologica Anton Dohrn, Villa Comunale, Naples Italy; 9 Friedrich Schiller University Jena, Institute of Inorganic and Analytical Chemistry, Lessingstrasse 8, 07745 Jena, Germany; 10 Sorbonne Université, CNRS, UMR 7232 Biologie Intégrative des Organismes Marins BIOM, Observatoire Océanologique, F-66650 Banyuls-sur-Mer, France; 11 School of Environmental Sciences, University of East Anglia, Norwich Research Park, Norwich, NR4 7TJ UK

**Keywords:** Comparative genomics, Molecular evolution, Genetic variation

## Abstract

Benthic diatoms are the main primary producers in shallow freshwater and coastal environments, fulfilling important ecological functions such as nutrient cycling and sediment stabilization. However, little is known about their evolutionary adaptations to these highly structured but heterogeneous environments. Here, we report a reference genome for the marine biofilm-forming diatom *Seminavis robusta*, showing that gene family expansions are responsible for a quarter of all 36,254 protein-coding genes. Tandem duplications play a key role in extending the repertoire of specific gene functions, including light and oxygen sensing, which are probably central for its adaptation to benthic habitats. Genes differentially expressed during interactions with bacteria are strongly conserved in other benthic diatoms while many species-specific genes are strongly upregulated during sexual reproduction. Combined with re-sequencing data from 48 strains, our results offer insights into the genetic diversity and gene functions in benthic diatoms.

## Abstract (web summary)

Available genomics studies have mostly focused on planktonic centric diatom. Here, the authors report the genome assembly of the marine biofilm-forming diatom *Seminavis robusta* and the resequencing data of a panel of accessions to reveal their evolutionary adaptations.

## Introduction

In contrast to planktonic environments, benthic habitats are complex and heterogeneous, characterized by sharp and sometimes dynamic microscale gradients of light, oxygen, nutrient availability, and redox state^1^. Benthic organisms are regularly exposed to inhospitable conditions such as extended periods of darkness, anoxia or the presence of toxic compounds such as sulfides^2^. Yet, illuminated surfaces in shallow aquatic systems are inhabited by dense and highly productive phototrophic biofilms which fuel food webs, modulate fluxes of carbon and nutrients, and can even stabilize sediments through the production of copious amounts of extracellular polymeric substances^3^.

In temperate and polar regions, phototrophic biofilms are frequently dominated by diatoms. These stramenopile microalgae are key players in aquatic ecosystems, accounting for up to 20% of global primary production^4^. They are uniquely characterized by a complex, bipartite silica cell wall and a particular size reduction–restitution life cycle^5^. Compared to the largely planktonic group of centric diatoms, the predominantly benthic and evolutionary younger pennate diatoms are far more species-rich^6^. This remarkable diversification is especially pronounced among the raphid pennates, being attributed to their heterothallic mating systems and active cell motility. Heterothally (differentiation in two or more mating types, as opposed to homothally in most centrics) promotes outcrossing, generating high levels of genetic diversity. Diatom cell motility, which is thought to be driven by an intracellular actomyosin cytoskeletal motor system linked to adhesive mucilage secretions from a cell wall slit termed the raphe, allows active positioning along chemical and physical microgradients within or on submerged surfaces. Importantly, motility of gametangial cells or gametes enables pheromone-guided mate finding, thus optimizing encounter success between opposite mating types in highly diverse and densely packed biofilms^7^. Combined, these two features may underlie the extraordinary diversity of raphid diatom species with often finely differentiated abiotic and biotic microniches^2^.

To date, diatom genomic studies have mostly focused on planktonic centric species or on pennate species that have colonized the planktonic environment. The first diatom genome that has been sequenced was the planktonic centric *Thalassiosira pseudonana*^8^, followed by the pennate *Phaeodactylum tricornutum*^9^, which evolved morphological plasticity to switch between benthic and planktonic morphotypes. More recent studies have extended our knowledge of the complexity of diatom genomes, some examples being the oleaginous pennate *Fistulifera solaris*^10^ with an allodiploid genome structure and the cold-adapted pennate *Fragilariopsis cylindrus* with a highly heterozygous genome showing allele-specific expression in response to environmental stresses^11^. Sequencing of diatoms with larger genomes, including the centric *Thalassiosira oceanica* (92 Mb)^12^ and the araphid pennate *Synedra acus* (98 Mb, also named *Fragilaria radians*)^13^, have shown that these species have more than twice the number of protein-coding genes than *T. pseudonana* and *P. tricornutum*, which suggests the traditional diatom models are underestimating gene diversity.

In this work, we explore the genomic features of the pennate raphid benthic diatom *Seminavis robusta* to improve our understanding of genome evolution in diatoms as well as to provide insights into the molecular basis of ecological adaptations to the benthos. *S. robusta* resides in biofilms in shallow coastal habitats^14^ and has been developed as a model system to study life cycle regulation, sexual reproduction, and ecology of benthic pennate diatoms^15^. Through the integrative analysis of the genome sequence of *S. robusta*, resequencing data of additional strains, detailed gene expression profiles, and gene information of 88 other diatom species, we shed light on key genes mediating differences in cell symmetry, motility and environmental adaptations between distinct groups of diatom clades.

## Results

### Genome characterization and expression atlas

**Fig. 1:**
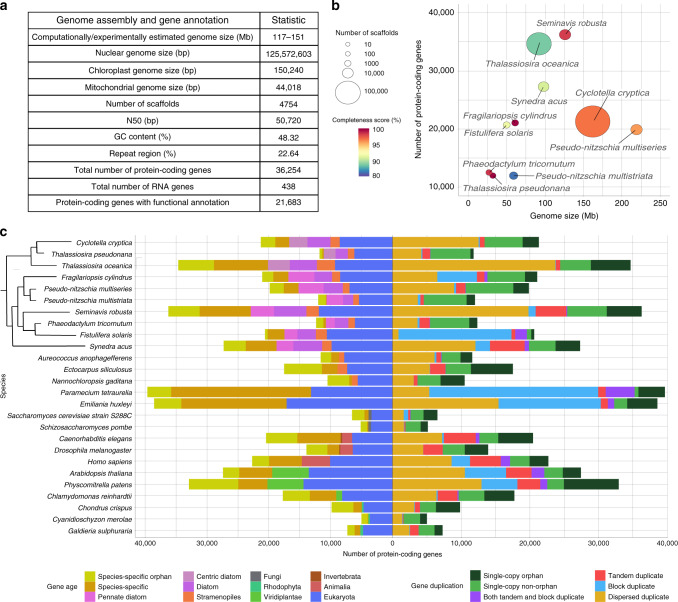
Genome properties for *S. robusta* and comparison with other sequenced diatoms. **a** Summary of the *S. robusta* genome assembly and gene annotation statistics. **b** Scatter plot showing genome assembly contiguity and gene family completeness score in sequenced diatom genomes. Every dot represents a diatom genome assembly. The *x* axis displays the genome size in Mb, whereas the *y* axis represents the number of protein-coding genes. Genome assemblies are colored according to the gene family completeness score in a rainbow scale from blue to red. The size of the circle indicates the number of scaffolds in the genome assembly. **c** Comparative genomics analysis among diatoms and other eukaryote species. Left side of the barplot represents the age of the genes inferred through phylostratification, whereas the right side represents the duplication information. The phylogenetic relationship between diatom species is shown in a cladogram.

Illumina paired-end sequencing in combination with long PacBio reads of the *Seminavis robusta* D6 strain, yielding 79× and 34× coverage, respectively, were used as input for genome assembly (Supplementary Table 1). A k-mer distribution analysis of the Illumina reads revealed a high level of heterozygosity (0.79%) and an estimated genome size of 117 Mb (Supplementary Fig. 1), while flow cytometry yielded an estimate of 151 Mb (standard deviation 17.65 Mb, Supplementary Note 1). Both PacBio and Illumina data were used to generate several genome assemblies and to compare the performance of different tools and integration strategies, keeping as a final assembly the one that obtained the best balance between contiguity, completeness and quality (Supplementary Table 2 and Supplementary Note 2). After removing four scaffolds corresponding to bacterial contamination (Supplementary Fig. 2), the assembly consisted of 4754 genomic scaffolds covering 125.57 Mb, which is within the range of estimated genome sizes, and had a scaffold N50 of 51 kb (Fig. 1a). This genome assembly was further experimentally validated through the successful amplification of 22 regions that had the expected size (Supplementary Fig. 3 and Supplementary Table 3).

Repeat detection analysis revealed that 23% of the *S. robusta* genome assembly consists of repeats and transposable elements (Supplementary Table 4). After masking these regions, the *S. robusta* genome was subjected to an initial round of gene prediction and manual curation, followed by the use of newly generated expression data to improve gene models and identify additional expressed genes. Besides nuclear RNA gene prediction, resulting in 54 rRNAs, 227 tRNAs, 46 snoRNAs, and 18 snRNAs, the chloroplast and mitochondrial genomes (Supplementary Fig. 4) were also annotated, leading to a total number of 36,254 protein-coding genes and 438 RNA genes (Fig. 1a). To date, *S. robusta* is the diatom with the largest number of predicted protein-coding genes, of which 88% show expression support (Supplementary Fig. 5), 63% share similarity to proteins from other eukaryotes and 60% are functionally annotated. Application of a phylogeny-based horizontal gene transfer detection procedure identified 1741 genes of putative bacterial origin^16^. Assessing the accuracy and quality of *S. robusta* gene models revealed the successful recovery of 99% of core gene families conserved in *Bacillariophyta* (Supplementary Note 2, Supplementary Fig. 6, and Supplementary Table 5), demonstrating the higher completeness of the *S. robusta* gene annotation compared with other diatoms with large (>90 Mb) genomes (Fig. 1b; Supplementary Table 6).

Newly generated expression data combined with existing data (Supplementary Table 7) were used to create a large-scale gene expression atlas profiling 31 different experimental conditions. This atlas comprises a total number of 167 samples (4.2 billion Illumina reads) and covers different sexual reproduction stages, abiotic stresses, and bacterial interaction-related treatments, the latter including responses to bacterial exudates as well as N-acyl homoserine lactones (AHLs), a class of signaling molecules involved in bacterial quorum sensing. To investigate the functional significance of the *S. robusta* protein-coding genes, a differential expression analysis was performed, obtaining a total of 27,963 (77%) differentially expressed genes.

### Gene family expansions driving adaptive evolution

**Fig. 2:**
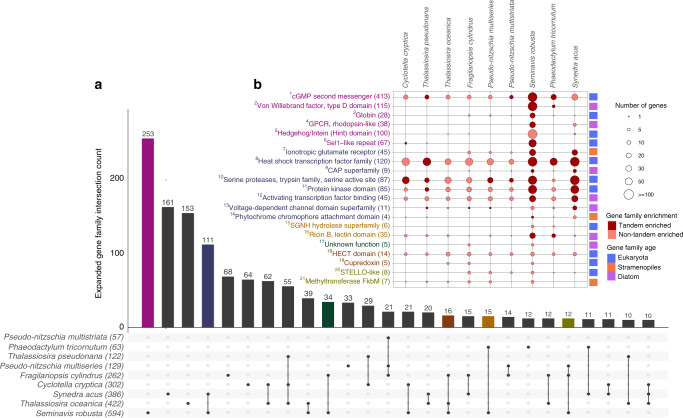
Species-specific and shared gene family expansions in diatoms. **a** Upset plot showing the intersection of gene family expansions in diatoms. Each row represents a diatom species, reporting the total number of expanded gene families within parenthesis. Black circles and vertical lines between the rows represent the intersection of expanded families between species. The barplot indicates the total gene family count in each intersection, displaying only intersections that contain at least ten gene families. Diatoms with a genome size > 90 Mb are highlighted in bold. **b** Examples of species-specific and shared gene family expansions in *S. robusta*. Each column represents a diatom species, and each row a given gene family showing expansion in *S. robusta*, indicating the total number of genes in *S. robusta* in parenthesis and matching the font color with the intersection subset in panel **a**. The size of the circles is proportional to the number of genes falling under the given gene family per species, whereas the color of the circles indicates if the gene family is significantly tandem-enriched. Source data are provided as a Source Data file. Numbers in superscript refer to families annotated in this Source Data file.

In order to study gene organization and evolution, we built the PLAZA Diatoms 1.0 platform (https://bioinformatics.psb.ugent.be/plaza/versions/plaza_diatoms_01/) to identify gene families based on the protein-coding genes from 26 eukaryotic genomes, which include nine other diatoms (Supplementary Table 8). Diatoms with the largest number of protein-coding genes (*S. robusta*, *T. oceanica*, and *S. acus*, Fig. 1b) had overall more families and a higher proportion of species-specific families compared with diatoms with smaller genomes such as *P. tricornutum* (Fig. 1c; Supplementary Note 3 and Supplementary Figs. 7–9). We identified 594 *S. robusta* expanded families (Fig. 2a; Supplementary Fig. 10), driven by different duplication mechanisms (Supplementary Fig. 11), containing 9178 genes. Despite the presence of a small number of block duplicates, no evidence for a recent whole-genome duplication was found. Several *S. robusta* expansions include families encoding for genes involved in molecular sensing, light signaling and motility (Fig. 2b; Supplementary Note 4). For instance, we found that the single-domain voltage-gated channels (EukCatAs) and the red/far-red light-sensing phytochrome (DPH) families expanded by dispersed and tandem gene duplicates, respectively. While the EukCatAs family has been shown to modulate gliding locomotion in raphid pennate diatoms through fast Na^+^/Ca^2+^ signaling^17^, the DPH family may be relevant for sensing the density and stress status of biofilms^18^.

*S. robusta* displayed a remarkably high number of tandem duplicates (4594 genes), which is also observed for *S. acus* (5670 genes), as well as several multicellular eukaryotes (Fig. 1c). A DNA coverage analysis indicated that the vast majority (81–84%) of these tandem gene duplicates, organized in gene cluster arrays, exist as distinct copies in the *S. robusta* genome, and are not an artefact of the assembly procedure (Supplementary Fig. 12). Tandem duplications have been shown to play an important role in accommodating responses to external stimuli and adaptation to rapidly changing environments^19^, as well as generating novel expression patterns through exon shuffling^20^. Of the 318 expanded families in *S. robusta* having tandem duplicates, 69 were significantly enriched in tandem duplicates and only six of these families were shared with *S. acus* (Fig. 2b), revealing that tandem-mediated family expansions are largely species-specific (Supplementary Fig. 13). *S. robusta* tandem gene duplicates mainly consisted of leucine-rich repeat (LRR) containing proteins, cyclases, heat-shock factors, serine proteases, ubiquitin ligases, ricin B-like lectins, rhodopsins, ionotropic glutamate receptors, polyketide enzymes, and globins, all genes that may be important for the adaptive evolution of this species (Supplementary Table 9).

**Fig. 3:**
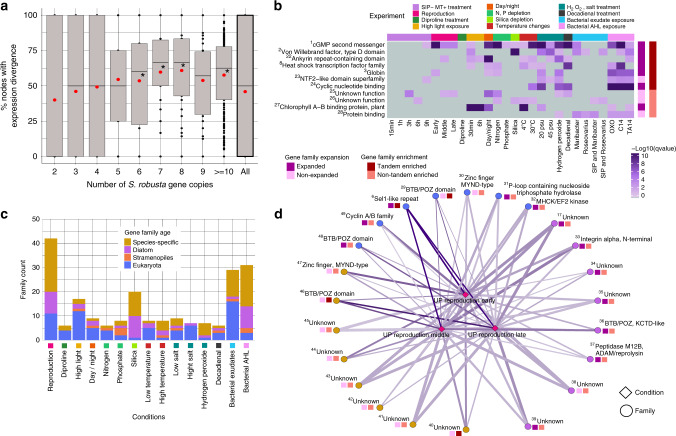
Expression analysis for *S. robusta* multi-copy gene families. **a** Expression divergence trend for multi-copy *S. robusta* families (*n* = 4444 families). The *y* axis denotes the percentage of nodes showing expression divergence in the phylogenetic tree of the family, while the *x* axis represents the number of *S. robusta* gene copies in the family. Average expression divergence percentages are indicated by red dots. Median expression divergence values significantly higher than the median of all nodes are highlighted with a star (*P*-value < 0.05, Wilcoxon rank-sum test, two-sided). **b** Heatmap showing pleiotropic families significantly enriched in upregulated genes for more than seven different conditions. The *x* axis represents the different conditions/experiments, whereas the *y* axis reports the families. The significance of the upregulation in a certain condition for a family is shown in –log_10_(*q*-value) scale highlighted by a color gradient from gray to dark purple. Expansion and tandem enrichment of each family are highlighted in different colors on the right side of the heatmap. **c** Barplot showing family counts with significant condition-specific expression. The *x* axis represents the different conditions/experiments, whereas the *y* axis represents the number of families having significant expression bias for that condition. The color of the bars denotes the family age distribution. **d** Network showing families with significant specific expression in the three reproduction stages available. Families are represented with circles, while conditions are represented with diamonds. The color of the circles denotes the family age following the same color code as panel **c**. The edge’s width denotes the fraction of genes in the family that shows upregulation for the given condition, while the edge’s color represents the significance of the enrichment, following the same color code as panel **b** from gray to dark purple. Expansion and tandem enrichment of each family are indicated by the squares next to the gene family circles, also following the same color code as panel **b**. Source data are provided as a Source Data file. Numbers in superscript refer to families annotated in this Source Data file.

As 70% of *S. robusta* genes are part of multi-copy gene families, we evaluated potential divergence and redundancy of gene duplicates by studying their expression profiles (Supplementary Fig. 14). We observed that families with an increasing number of genes tended to display higher expression divergence than smaller families (Fig. 3a), and that families showing expression divergence are significantly enriched in eukaryote and diatom age classes. Conversely, families showing expression conservation are significantly enriched in the species-specific age class (Supplementary Fig. 15). These results indicate that globally gene duplication is associated with expression divergence and that expression divergence increases with the age of gene duplicates. However, 152 cases of diatom-specific or species-specific families showing strong expression divergence were found (Supplementary Fig. 15 and Supplementary Table 10), revealing that rapid expression evolution may also occur for specific gene functions encoded by young gene families. To determine under which conditions *S. robusta*’s families are expressed and in which processes they are involved, we identified families significantly enriched in upregulated genes under seven or more diverse experimental conditions (referred to as pleiotropic families) or in few specific related conditions.

Eight out of the 11 pleiotropic families were expanded and/or enriched in tandem duplicates, and several encoded proteins involved in signaling (Fig. 3b), indicating a strong link between gene family evolution, tandem duplication, and *S. robusta*’s transcriptional response to environmental stimuli. The family that showed the highest pleiotropic signal is involved in cyclic guanosine monophosphate (cGMP) biosynthesis, which was shown to play a key role during the onset of sexual reproduction in *S. robusta*^15^ as well as *Pseudo-nitzschia multistriata*^21^. A recent study suggests that this signaling pathway might also be involved in the response to bacteria^22^. Our expression data corroborate these functions, but also indicate that the cGMP-related signaling may have a much broader role than previously thought, showing significant upregulation in a wide range of abiotic stresses (Fig. 3b). For a heat-shock transcription factor family (HSFs) expanded in diatoms^9^ and showing pleiotropic expression in *S. robusta*, we identified that this expansion happened through tandem duplication in several diatoms (Fig. 2b). Interestingly, the *S. robusta* NTF2-like tandem-enriched pleiotropic family had 19/68 copies annotated with the polyketide cyclase SnoaL-like domain. Enzymes for polyketide metabolism are absent in some protist lineages but expanded in others such as dinoflagellates and haptophytes, suggesting that the evolution of these compounds may have played an important role in their ecological success^23^.

Von Willebrand factor, type D domains (vWDs) are found in numerous extracellular proteins and are believed to be involved in protein multimerization. For example, several adhesive proteins contain vWDs (e.g., zonadhesin, sea star foot protein, diatom adhesive trail proteins), which are thought to be important for maturation of the adhesive into multiprotein complexes^24,25^. Sixty-one of the *S. robusta* vWD containing proteins also held the diatom-specific GDPH domain, hypothesized to be important for secreted diatom proteins with adhesive functions (e.g., motility, mucilage pads, gamete fusion)^25^. The identified pleiotropic tandem-enriched vWD family is highly abundant in raphid species and was significantly upregulated in ten different conditions in *S. robusta*, half of these related to bacterial interactions, suggesting that they might be important for bacterial recognition, motility, and adhesion. Hence, the expansion of the vWD family in *S. robusta* may reflect a key adaptation to highly heterogeneous benthic environments, which are inhabited by very diverse and dense bacterial populations compared with the water column.

In contrast to the wide expression of pleiotropic gene families, more than 200 families were identified showing significant enrichment for upregulation in one or a limited number of related conditions (Supplementary Note 5 and Supplementary Figs. 16–19). Notably, many of these families were species or diatom-specific and responsive to either sexual reproduction, silica depletion, or bacterial interaction (Fig. 3c). Hence, the strong specific expression of these families indicates a role in these distinctive diatom traits. Diatoms are unique by having a silica cell wall and a size reduction–restitution life cycle, that for many diatoms, includes sexual reproduction^5^. Twenty-three out of the 42 reproduction responsive families are strongly expressed in the three profiled sexual stages (Fig. 3d). The 12 families with functional annotation encoded for proteins related to protein–protein interactions (BTB/POZ domain)^26^, U box ubiquitin ligases (Sel1 repeats), and potential candidates for cell–cell recognition of gametangia and/or fusion of gametes (integrins and M12B domain)^27^. In addition to these, the cyclin A/B family was enriched in all stages of sexual reproduction, indicating that some of these genes have a specific regulatory function during meiosis (Supplementary Fig. 19). Finally, eight of these reproduction responsive families showed a simultaneous enrichment for hydrogen peroxide responsive genes (Supplementary Fig. 16A), suggesting that reactive oxygen species signaling plays an important role during sexual reproduction.

*S. robusta* is found in shallow coastal habitats, often as part of subtidal biofilm communities, which can experience pronounced temperature changes. Seventy-one families showed specific expression toward bacterial interaction experiments (Fig. 3c; Supplementary Fig. 16B, C) and eight families toward high temperature (Supplementary Fig. 17). Half of these families were species-specific and/or lack functional annotation, but their strong specific expression now sheds light on the biological processes they are putatively active in. The functionally annotated bacterial interaction responsive families were involved in intracellular signaling, oxygen sensing, detoxification, oxidative stress responses, and cell adhesion. As an example, Arf GAPs can function as regulators of specialized membrane surfaces implicated in cell migration^28^. Together with their strong upregulation in the bacterial interaction experiments (Supplementary Fig. 16C), we suggest that the tandem duplication-driven expansion of these genes may be important for *S. robusta* cell adhesion and movement during biofilm formation. In contrast, the expansion by tandem duplicates of two families annotated as LRRs and ionotropic glutamate receptors (iGluRs) are relevant for *S. robusta*’s signaling during high-temperature acclimatization (Supplementary Fig. 17).

### Intra-species gene variability and patterns of gene selection

**Fig. 4:**
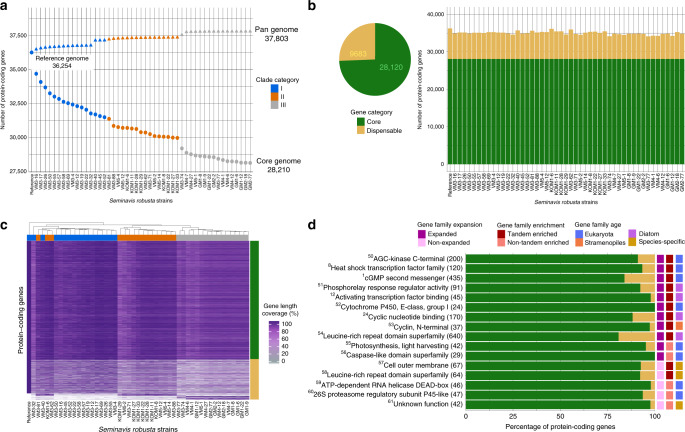
*S. robusta* within-species variability using a gene-based pan-genome analysis. **a** Representation of reference, core and pan gene size. The size of pan genome increases with each added strain up to 37,803 protein-coding genes, whereas the size of core genome diminishes to 28,120 protein-coding genes. Clade category color code refers to the population groups described in ref. ^14^. **b** Number of core and dispensable genes per *S. robusta* strain. The pie chart shows the total gene count, where core genes are genes present in all strains, dispensable genes are genes present in a subset of strains. **c** Percentage of gene length coverage by short read for all pan genes for each strain. The *x* axis represents the *S. robusta* strains, whereas the *y* axis represents all protein-coding pan genes. The percentage of horizontal gene coverage is highlighted by a color gradient from white (0%) to dark purple (100%). Gene categories are labeled on the right side of the *y* axis following the color code of panel **b**, whereas clade categories are labeled on the upper part of *x* axis following the color code of panel **a**. **d** Set of gene families that are significantly enriched in core genes. The *x* axis represents the percentage of protein-coding pan genes that are core or dispensable, following the color code of panel **b**, while the *y* axis represents gene families, denoting in parenthesis the total number of pan genes belonging to that gene family (reference and de novo genes). Expansion, tandem enrichment, and age of each family are highlighted in different colors on the right side of the *y* axis. Numbers in superscript refer to families annotated in Source Data file from Fig. 3. Source data underlying Fig. 4a, b are provided as a Source Data file.

Resequencing data from 48 different *S. robusta* strains, representing three genetically distinct clades sampled from a small geographic region^14^, was used to estimate the *S. robusta* pan-genome size and to validate the number of predicted protein-coding genes as well as the observed family expansions. While the core genome refers to genes present in all strains, the pan genome encompasses these core genes and dispensable ones present in only a subset of strains (Fig. 4a). We identified 1549 de novo genes absent from the reference strain and therefore estimated the *S. robusta* pan genome to cover 37,803 genes. Assessing the gene content diversity across all 49 *S. robusta* strains revealed that 28,120 (74%) genes were core while the remaining 9683 (26%) genes represented the potential dispensable fraction (Fig. 4b). From this dispensable fraction, we found 2551 (6.7%) softcore genes (present in >= 95%, but not all of the strains), indicating that the fraction of core genes probably ranges between 74–81% (Supplementary Fig. 20). Globally, the resequencing of individual *S. robusta* strains indicated an average gene count of 35,012 protein-coding genes, therefore confirming the number of genes identified in the reference strain (Fig. 4b). Clustering the strains by short-read DNA gene length coverage recovered the three genetically distinct clades and hybrid strains previously described^14^ (Fig. 4c), supporting our presence/absence variation classification and showing this strain variability is not simply an artefact of the procedure. In total, 73% of the reference tandem gene duplicates and 83% of the genes in expanded families were classified as core, indicating a strong conservation of duplicated genes in the different *S. robusta* strains. In fact, 13/16 families significantly enriched in core genes were expanded and/or enriched in tandem duplicates (Fig. 4d). These families are related to essential functions (e.g., light-harvesting involved in photosynthesis, metacaspases involved in proteolysis, cyclins controlling cell cycle regulation) but also encompass tandem-enriched expansions involved in signaling, including HSF, cGMP, and LRR related proteins. In contrast to these, 122/151 families significantly enriched in dispensable genes lacked functional annotation. Among the remaining annotated dispensable genes, we found telomeric proteins, which are known to undergo rapid evolution, in particular, it has been shown that gene duplicates create telomere paralogs with novel functions in telomere replication and chromosome end-protection^29^. The observation that 63% of the *S. robusta* dispensable genes in the reference genome were expressed and several of these encoded for transporters and metabolic proteins indicates this genomic variation represents transcriptionally active genes, potentially offering a template for phenotypic or physiological evolution in this diatom.

The *S. robusta* dispensable gene fraction is comparable with what has been described in the marine phytoplankton *E. huxleyi*, where 33% of the reference genes were missing in other strains^30^, and plants (20–27% dispensable genes in *Brassica oleracea, Helianthus annuus*, *Brachypodium distachyon*, and *Solanum lycopersicum*^31^). However, determining the size of the dispensable gene content using resequencing data of ten geographically distant accessions in *P. tricornutum*^32^ revealed that only 4.46% of the genes are absent in at least one accession (558/12,517 core genes, 172 de novo-assembled genes). The six times higher fraction of dispensable genes in *S. robusta* suggests much more de novo gene evolution in this benthic diatom compared with *P. tricornutum*, although convergent gene loss due to long-term culturing for the latter species might provide an alternative explanation.

To evaluate conservation at the amino acid level and identify *S. robusta* genes that are under positive or purifying selection, we used the resequencing strains to generate a high-confidence coding single-nucleotide polymorphism (SNP) data set (see Supplementary Note 6 and Supplementary Fig. 21). The average estimates of nucleotide diversity at nonsynonymous (π_N_) and synonymous (π_S_) sites were 0.003 and 0.022, respectively. This indicates that two homologous sequences drawn at random from different strains will on average differ by 2.2% on synonymous sites and 0.3% on nonsynonymous sites. The average π_N_/π_S_ ratio was 0.14, which is smaller than what has been reported in other organisms such as *O. tauri* (0.20)^33^, *C. reinhardtii* (0.22)^34^, or the pennate diatom *P. tricornutum* (0.43)^32^. These differences reveal that globally the analyzed amino acid composition of *S. robusta* genes undergoes a higher level of purifying selection, which is expected if it has a larger effective population size, as suggested by the higher π_S_. Furthermore, the distribution of the π_N_/π_S_ ratio was estimated on individual genes. Interestingly, genes with higher π_N_/π_S_ ratios, suggesting positive selection, were significantly enriched in genes upregulated during early, middle, and late sexual reproduction stages, showing that genes involved in sexual reproduction are more prone to undergo diversification in their amino acid sequence, resulting in potential new protein functions or reflecting coevolution of receptor/ligand systems. Although some of these genes belonged to pleiotropic families (e.g cGMPs and vWDs) and families with reproduction expression bias (e.g., BTB/POZ domain and U box ubiquitin ligases) discussed previously, the vast majority were, however, genes of unknown function (100/128 genes) and/or species-specific single copy (63/128), representing interesting candidates for future experimental characterization.

In addition, median π_N_/π_S_ values were further analyzed to compare levels of purifying selection between distinct gene groups. Focusing on different gene age classes, we observed that gene age is positively correlated with purifying selection, with old genes being significantly more constrained than young genes (Supplementary Fig. 22A). Genes with homologs in other eukaryotes are also more likely to be functionally annotated than species-specific genes; hence, poorly characterized genes showed significantly less purifying selection than genes having functional annotations (Supplementary Fig. 22B). Likewise, dispensable genes had significantly higher π_N_/π_S_ values than core genes (Supplementary Fig. 22C), suggesting reduced functional constraint for variable genes in the *S. robusta* species complex. Strikingly, single-copy genes did not display significantly more purifying selection than duplicated genes. In contrast, when comparing between gene duplicate types, tandem duplicates were under less constraint than dispersed duplicates (Supplementary Fig. 22D), indicating that tandem duplicates are more susceptible to amino acid changes, representing the potential evolution towards novel functionalities. We further observed that genes that are not expressed or show condition-specific expression were less constrained than genes that are broadly expressed (Supplementary Fig. 22E, F), the latter being frequently associated with housekeeping functions. Overall, our findings reveal that different levels of purifying selection act on different subsets of the *S. robusta* gene repertoire, and that dispensable genes as well as genes showing expression under few experimental conditions are less evolutionary constrained, suggesting they might only be relevant in specific environments.

### Genes involved in cell motility and benthic adaptations

The Marine Microbial Eukaryote Transcriptome Sequencing Project (MMETSP) database^35^ was used as a reference data set to find *S. robusta* signature genes that might be implicated in specific morphological or ecological properties. These genes were identified by first determining, for each *S. robusta* gene, homologs in a set of 88 diatoms and subsequently selecting those genes predominantly present in pennate, raphid, and benthic species, compared with centric, araphid, and planktonic species, respectively (see “Methods”). The differential expression of these *S. robusta* signature genes was further inspected to give more insight into the biological roles of these genes.

**Fig. 5:**
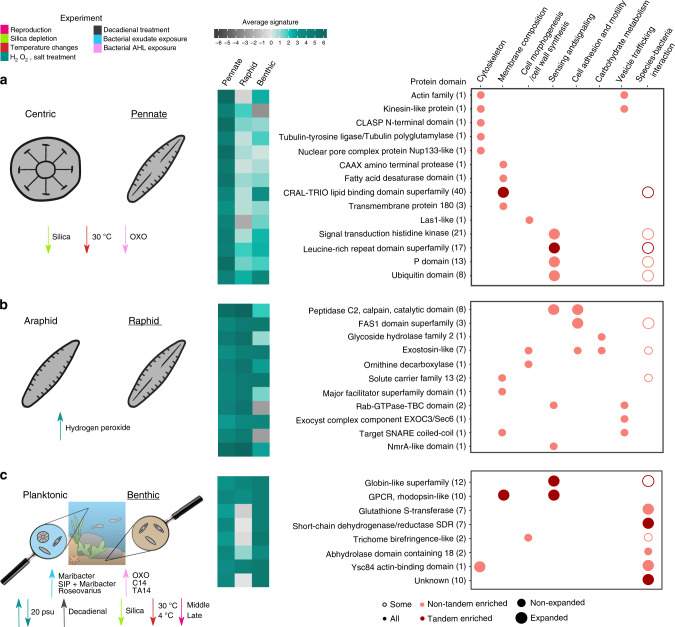
Selection of signature genes showing strong clade-specific conservation. Significant enrichment for differential expression in the *S. robusta* transcriptome of genes showing the specified signature is highlighted with downward (for downregulation) or upward (for upregulation) arrows colored by experiment. Each row is a protein domain, the number of genes showing the signature, and having that protein domain is indicated in parenthesis. If any of the genes containing one of the highlighted protein domains belong to an expanded and/or tandem-enriched family, this is encoded by the size and color of the circles. The fill of the circles indicates if all or some genes with a given protein domain are upregulated during bacterial interaction experiments. The average pennate/raphid/benthic signature per protein domain is highlighted by a color gradient from dark gray (−6) to dark green (6). A selection of genes with high pennate signature is shown in panel (**a**), which high raphid signature in panel (**b**) and with high benthic signature in panel (**c**). Source data are provided as a Source Data file.

A significant number of genes showing high pennate signature were downregulated during silica depletion (55/630 genes). As many of these signature genes were related to cytoskeleton and membrane composition, this suggests they might play a role in cell wall and/or pennate cell shape formation (Fig. 5a; Supplementary Note 7 and Supplementary Figs. 23–28). Some examples of these cytoskeleton proteins include an actin-related protein 10 and a microtubule motor kinesin, modulating the dynein-mediated movement that contributes to nuclear migration during mitosis^36^. Another gene with high pennate signature encodes a tubulin–tyrosine ligase/tubulin polyglutamylase, involved in the post-translational modification of tubulins, as well as a CLASP N-terminal protein, implicated in the attachment of microtubules to the cell cortex and therefore regulating their stability. The loss-of-function mutation of the latter gene in *A. thaliana* has shown to result in various plant growth reductions, cell form defects, and reduced mitotic activity^37^, indicating that CLASP genes can potentially contribute to differences between pennate and centric diatoms in cell division and expansion. We also identified the nucleolar Las1-like protein showing high conservation in pennate diatoms, which has been linked to cell morphogenesis and cell surface growth in yeast^38^ and showed strong upregulation during early sexual reproduction in *S. robusta*. Related to membrane composition, we identified a CAAX amino terminal protease, inserted in the bilayer structure of the membrane, that is potentially implicated in protein and/or peptide modification and secretion^39^. The upregulation of this protease during early sexual reproduction hints toward a role in recognition during cell pairing between opposite mating types. In contrast, we found a fatty acid desaturase with high pennate signature upregulated during low temperatures, indicating that centric and pennate species might have also evolved different mechanisms to increase membrane fluidity for cold adaptation. The finding of 40 genes belonging to the CRAL-TRIO lipid binding superfamily further suggests the existence of differences in phospholipid metabolism^40^.

In addition to these pennate signature genes, we also searched for raphid-specific and benthic-specific traits in the diatom phylogeny (Supplementary Fig. 29), finding proteins widely conserved in most raphid species and others more restricted to certain benthic species. In particular, within the 241 genes showing a high raphid signature, several genes related to cell adhesion and motility were found, including three proteins containing the ancient cell adhesion domain fasciclin (FAS1) and eight with a “peptidase C2, calpain” domain (Fig. 5b; Supplementary Note 7 and Supplementary Figs. 30–33). Raphid species are responsive to intracellular calcium, playing a role in changing the speed and direction of locomotion^41^. Calpain proteins are calcium-responsive intracellular proteases that are implicated in the regulation of cell migration by controlling the dynamics of both integrin-mediated adhesion and actin-based membrane protrusion, enabling cell movement by modifying these adhesion sites^42^. To achieve their gliding motility and underwater adhesion, raphid diatoms secrete carbohydrate-rich extracellular polymeric substances (EPS)^25^. In line with this, multiple proteins with strong raphid conservation related to carbohydrate metabolism were identified, comprising one glycoside hydrolase and seven exostosin-like proteins, the latter encoding glycosyltransferases involved in the synthesis of sulphated proteoglycans present in the extracellular matrix of mammalian cells^43^. Interestingly, sulphated polysaccharides/proteoglycans have been identified in the EPS and cell wall-associated glycoproteins of raphid benthic diatoms, such as *Stauroneis amphioxys* and *Craspedostauros australis*^44,45^. The strong conservation of several of these exostosin-like proteins in different raphid benthic species (Supplementary Fig. 33) hints toward their importance for EPS synthesis in these diatoms. In addition, the presence of a raphid-specific ornithine decarboxylase, upregulated during reproduction, further suggests there are unique enzymes in raphid species controlling cell wall synthesis, since the inhibition of ornithine decarboxylase, which affects polyamine biosynthesis, has shown to result in a dramatic alteration of diatom silica structure^46^. Furthermore, several raphid-specific membrane transporters were found, as well as proteins containing the “Rab-GTPase-TBC”, “exocyst complex component EXOC3/Sec6”, or “target SNARE coiled-coil” domain, the latter three encoding key components related to vesicle trafficking^47^.

Benthic diatoms are frequently part of illuminated surfaces in shallow aquatic systems that are inhabited by dense and highly productive phototrophic biofilms, which are characterized by highly variable oxygen and light conditions. To identify genes that can be linked with molecular components explaining the success of diatoms in these environments, we explored the *S. robusta* gene functions showing strong conservation in other benthic diatoms. Within the set of 492 benthic signature genes, we observed a strong overrepresentation of different genes that can directly be related to oxygen and light signaling as well as diatom–bacteria interactions (Fig. 5c). Twelve globin-like *S. robusta* genes with high benthic signature, organized in three different families, were identified. Globins have been found across all phyla of life, meaning that the oxygen transport function of vertebrate hemoglobins is a relatively recent adaptation and that the early globin functions were enzymatic and oxygen-sensing. Dissolved and particulate carbon and nutrient levels are higher within benthic habitats compared with the water column, increasing microbial numbers and heterotrophic activity. As a result, oxygen is quickly depleted, leading to a steep redox gradient in the sediment^48^, which implies benthic species may have evolved mechanisms to sense oxygen concentration changes and regulate chemotaxis towards optimal conditions. One of the globin-like signature families is within the top pleiotropic families expanded by tandem duplication (Fig. 3b; Supplementary Fig. 34) and is restricted to pennate diatoms (Fig. 2b), while the other two families encode for benthic-specific globins (Supplementary Fig. 35). Whereas one of these benthic-specific families showed strong upregulation under hydrogen peroxide treatment, future experiments are needed to clarify the role of these globin-like proteins in sensing oxygen concentrations.

We identified ten putative G-protein-coupled receptor (GPCR) rhodopsin-like proteins having high benthic signature, of which seven belong to one family expanded by tandem duplication (Fig. 2b). In the ocean, the spectral composition of light becomes progressively dominated by blue–green light (400–500 nm) with increasing depth. In biofilms, differential absorption of light by phototrophs and particulate and dissolved matter can contribute to variability in the spectral composition and intensity of available photosynthetically active radiation. Hence, benthic diatoms might have expanded and/or evolved their gene repertoire for light sensing. GPCR rhodopsin-like receptors transduce extracellular signals of a wide range of stimuli including light^49^. The existence of homologs to bacterial proteorhodopsins in some diatoms has been suggested to represent an alternative ATP-generating pathway, especially in iron-limited regions like the Southern Ocean^50^. A phylogenetic analysis revealed that the tandem-enriched family containing GPCR rhodopsin-like proteins with high benthic signature is not similar to these proteorhodopsins, rather, their members are more related to rhodopsin-like proteins found in other eukaryotes (Supplementary Fig. 36A). A differential expression analysis under different light conditions^51^ of the GPCR rhodopsin-like protein ortholog in *P. tricornutum* showed that this gene is specifically upregulated under blue–green light exposure (Supplementary Fig. 36B), confirming our hypothesis that this family plays an important role in light sensing. Apart from *S. robusta*, *Amphora* and *Trybionella* are other diatom genera containing these globin-like and GPCR rhodopsin-like signature genes (Supplementary Fig. 29), confirming that these traits are present in different clades of benthic diatoms.

The enrichment of benthic signature genes upregulated during bacterial interaction experiments (69 genes, Supplementary Fig. 37) strongly correlates with a benthic lifestyle, where diatom–bacteria encounters are frequent due to high cell packing in biofilm communities. Secretion of EPS by benthic diatoms and their physicochemical properties are important factors driving biofilm formation, which has been shown to be strongly influenced by interaction with bacteria and/or their extracellular substances^52^. Here, we found one of the previously mentioned exostosin-like proteins, as well as a trichome birefringence-like protein, both potentially related to benthic EPS synthesis. In particular, the latter protein is responsible for the O-acetylation of polysaccharides in bacterial biofilm formation, suggesting O-acetylation may also play an important role in benthic diatom biofilm architecture^53^. Benthic signature genes also contained several NAD- or NADP-dependent oxidoreductases and detoxification enzymes (Supplementary Fig. 37), covering seven short-chain dehydrogenases/reductases (SDRs) and seven glutathione S-transferases (GSTs). While SDRs are enzymes of a great functional diversity, GSTs are often involved in the detoxification of reactive oxygen species (ROS) and xenobiotics, indicating that benthic diatoms have an evolved gene set to control ROS homeostasis and increase oxidative stress defense in biofilms^54^.

## Discussion

The reported *S. robusta* genome sequence and its integration with resequencing data from 48 additional strains, large-scale expression profiling, and transcriptome data from different classes of diatoms, provided a unique opportunity to shed light on the genome complexity and evolution in benthic diatoms. To date, *S. robusta* is the diatom with the largest number of protein-coding genes, of which 40% are of unknown function and 60% are diatom or species-specific. The strong specific expression pattern of many of these genes has provided valuable insights into the biological processes they are involved in, including 42 families related to sexual reproduction and 71 families showing specific responses to bacterial interactions. Our findings therefore reveal previously unknown candidate genes potentially controlling specific diatom traits. We show that the large number of *S. robusta* genes that originated through gene family expansions are conserved in the *S. robusta* species complex and are extending the gene repertoire of this species for molecular sensing, light signaling, and motility. Tandem gene duplication is a prominent feature of pleiotropic gene families showing a complex pattern of wide-spread regulation in numerous conditions. Adaptation to the benthos through tandem gene duplication has also been observed in the penaeid shrimp^55^, which shares with *S. robusta* the iGluR expansion. In particular, rhodopsin-like proteins, globins, and detoxification enzymes seem to be key players in the ecological adaptation of not only *S. robusta* but also of other benthic diatoms that dominate marine biofilms. Whereas the large fraction of dispensable genes in *S. robusta* indicates a major difference in gene space variation compared with *P. tricornutum*, the phenotypic, ecological, and physiological consequences of missing genes in specific strains remain to be seen. Future studies of additional diatom species will help to identify and better define their adaptations to the benthic lifestyle, paving the road to understand the extraordinary evolutionary success of the pennate diatoms.

## Methods

### DNA extraction and genome size estimation

Both Illumina and PacBio technologies were used for sequencing of *S. robusta* D6 strain mating type plus (MT+) to take advantage of short-read (better quality) and long-read (better contiguity) sequencing approaches. Diatoms have silicified cell walls, which means that the high-molecular-weight (HMW) DNA extraction is not trivial. Harsh conditions are needed to break the wall, which also affects the integrity of the chromosomal DNA. Our final assembly (see next section) is based on a hybrid data set combining 18 million Illumina paired-end reads as well as >1 million PacBio long reads (Supplementary Table 1). In particular, Illumina DNA was extracted using a DNeasy Plant Mini Kit (Qiagen), paired-end libraries were prepared with an insert size of 500–800 bp, and sequencing was performed on a 2 × 300-bp Miseq system. In the case of PacBio, different HMW DNA extraction protocols were tested, Blue Pippin size selection was applied to enrich for long fragments, libraries were prepared with an insert size of 10,000 bp, and sequencing was performed in two different locations: ten SMRT cells were sequenced on the RSII at VIB Nucleomics Core (Leuven, Belgium), whereas an extra one SMRT cell was sequenced on the RS at GATC Biotech AG (Konstanz, Germany). In all scenarios, the obtained average read length was around 5 kb (Supplementary Table 1), which is lower compared with standard PacBio read lengths applied on other non-diatom species. A comparison with the *Fragilariopsis cylindrus* genome^11^ where the average PacBio read length was 2.4 kb reveals our long reads are of good quality. More details about DNA isolation methods and genome size estimation by flow cytometry are found in Supplementary Note 1. In addition, k-mer distribution statistical approach was used to estimate the genome size, repeat content and heterozygosity (Supplementary Fig. 1).

### Genome assembly and repeat identification

Due to the complexity caused by the heterozygosity, several genome assemblies were generated to compare the results of different tools and assembly integration strategies, including Illumina-only, PacBio-only as well as hybrid approaches combining the Illumina and PacBio data (Supplementary Table 2 and Supplementary Note 2). The ultimate chosen genome assembly was computed using the following protocols: (i) Illumina paired-end reads were assembled into contigs using the haplotype-aware Platanus v1.2.4^56^ tool, (ii) a heterogeneity cleaning process was executed to identify potential redundant sequences still present in the assembly based on a pairwise comparison using BLASTn v2.3.0^57^ (>85% identity, >75% coverage), keeping the largest sequence as non-redundant (strategy similar to redundans pipeline^58^), (iii) an extra round of scaffolding and gap-closing steps with Platanus was applied, (iv) PacBio reads were integrated using PBJelly v15.8.24^59^; a second heterogeneity cleaning process was performed onto the generated hybrid assembly, (vi) a final re-scaffolding with PBJelly was executed. The redundant sequences resulting from the heterogeneity cleaning comprised a total of 12.07 Mb. One-third of the Illumina paired-end reads that did not map to the reference genome did map to these sequences, revealing that 94% of all Illumina reads are present in the assembled sequences.

Both de novo-based and homology-based approaches to mask repeats prior to gene prediction were employed by using RepeatModeler and RepeatMasker^60^. A summary of the detected repeats in the *S. robusta* genome and the detailed protocol can be found in Supplementary Table 4.

### Gene prediction and functional annotation

Reads from ten different RNA-Seq experiments in *S. robusta*, that were available when this analysis started (see Supplementary Table 7), were employed as a training to infer gene predictions using the BRAKER1 pipeline^61^. The resulting gene prediction contained 35,265 protein-coding genes and was named as gene annotation v1.0. To further improve the gene annotation, manual curation of the *S. robusta* expanded gene families (582 curated genes in total) was performed using the ORCAE interface (gene annotation v1.1). Next, reads from 21 newly generated RNA-Seq samples were trimmed (see Supplementary Table 7), and mapped to the genome assembly using STAR v2.5.2^62^. These alignments were used to run the Genome-guided Trinity De novo Transcriptome v2.6.6 and PASA v2.3.3 pipeline^63^ in order to add untranslated regions (UTRs) (to 14,634 genes) and additional detected expressed gene models (938 genes), generating the final gene annotation v1.2 with a total number of 36,254 genes. The assessment of the quality of these gene models is described in the Supplementary Note 2.

Functional annotation of the protein-coding genes was performed by integrating the results from three different approaches. First, InterProScan v5.31^64^ was run (used applications: Phobius, SUPERFAMILY, PANTHER, Gene3D, Hamap, Coils, ProSiteProfiles, TIGRFAM, SMART, CDD, PRINTS, PIRSF, ProSitePatterns, Pfam, SignalP_EUK, ProDom, TMHMM) to scan our sequences for matches against the InterPro protein-signature database. Second, eggNOG-mapper v1^65^ was executed with DIAMOND mapping mode, based on eggNOG 4.5 orthology data. Third, a consensus functional annotation per gene was computed based on similarity searches following the AnnoMine pipeline described in^66^.

Noncoding RNA genes were predicted using Infernal v1.1.2^67^ (*e*-value < 10e−03, coverage >= 90%). The predictions that overcame the defined thresholds were manually checked in the Rfam database for the taxonomic origin, retaining mainly eukaryotic hits. In the specific case of tRNA genes, tRNAscan-SE v1.31^68^ (default parameters) was also executed and the results were merged with the Infernal output.

### *S. robusta* gene expression analysis

Cultures of *S. robusta* were subjected to 31 different conditions (167 samples) encompassing sexual reproduction, abiotic, and biotic stress. RNA-Seq data were generated for 83 samples, and were complemented with previously published data sets. The transcriptomic data were produced by subjecting *S. robusta* cultures to various treatments followed by RNA extraction and Illumina sequencing. A detailed overview of conditions and procedures can be found in Supplementary Table 7.

The sequenced reads were mapped to the longest isoform of *S. robusta* gene models with UTRs using Salmon v0.9.1^69^ (index using–type quasi -k 31, quantification using -l A parameter). Gene-level abundances of all 167 samples were imported in R using the tximport package v1.8.0, and transcript per million (TPM) values were calculated. A gene was considered to be expressed when TPM > = 2. For the differential expression analysis, genes with very low counts were filtered, retaining only genes with >1 count per million (CPM) in at least three samples. Out of a total of 36,254 genes, 32,273 genes were retained after filtering. Counts for each experiment were loaded into a separate DGEList object from the R package EdgeR^70^. TMM normalization factors were used as an offset to correct for differences in sequencing depth and RNA composition. After estimating tagwise dispersion, negative binomial GLMs were computed for every gene, and contrast matrices were designed to define 31 conditions (see Supplementary Table 7). To test for differential expression, likelihood ratio tests (LRT) were carried out. To limit the number of differentially expressed genes, we tested for differential expression relative to a log_2_ (fold change) threshold of ±1 with the glmTreat() function in EdgeR. FDR adjusted *P*-values were calculated for each comparison using the Benjamini–Hochberg correction, setting an adjusted *P*-value < 0.05 threshold for differential expression.

### Comparative genomics analysis

All *S. robusta* predicted genes were loaded into a diatom-centered version of the PLAZA comparative genomics platform^66^, containing 26 eukaryotic genomes (Supplementary Table 8). A detailed list of tools used to build PLAZA Diatoms 1.0 is shown in Supplementary Table 11. First, an all-against-all protein sequence similarity search was performed to subsequently delineate gene families. Families with more than three members and less than one thousand (from all species) were subjected to a multiple sequence alignment, followed by filtering and trimming and a phylogenetic tree construction using an approximate maximum likelihood method. Nearly single-copy families, having at least one and at most two gene copies per species, recovered across all diatoms were selected and the filtered and trimmed multiple sequence alignments for these families were concatenated per species (913 families, 269,486 amino acid positions). This alignment was used to infer the diatom species tree. Expanded families were delineated by calculating the *Z*-score profile of the gene copy number across all diatoms excluding the allodiploid *F. solaris*. Families where the variance was larger than two and the *Z* score for a particular species was larger than three, were deemed expanded in that species. To assign genes or families to age classes, the taxonomic scope of each gene/family was evaluated using phylostratification^71^. Gene families significantly enriched in upregulated genes for a specific condition were computed using hypergeometric distribution with a *q*-value cutoff of 0.05 and a minimum of two hits. The same protocol was applied for computing families significantly enriched in core/dispensable genes (see next section). Gene duplication type and collinear regions, i.e., regions with conserved gene content and order, were also identified. Block duplication, also known as segmental duplication is the duplication of a whole genomic fragment. Here, a block is defined as consisting out of at least three anchor gene pairs, where a pair consists of genes belonging to the same gene family, which are separated by maximal 15 genes (gap size) and blocks are separated by at least 15 genes (cluster gap) when belonging to the same scaffold. Tandem genes on the other hand belong to the same gene family and form gene cluster arrays, which are located within 15 genes of each other (tandem gap). Dispersed duplicates are duplicated genes within a gene family, but are not found to have originated through either a block or a tandem duplication event. Genes categorized as tandem duplicates were validated through a DNA coverage analysis (Supplementary Fig. 12) and InterPro domain significant enrichment was calculated using the same protocol described before for differentially expressed genes (Supplementary Table 9).

### Identification of core and dispensable protein-coding genes

Adaptor-trimmed paired-end 151-bp reads were obtained from the Nucleomics Core Facility (VIB, Leuven, Belgium) for 48 different *S. robusta* strains. Quality of these reads was assessed using FastQC v0.11.4, and then they were uniquely aligned to the D6 reference genome using the BWA-MEM v0.7.5a tool^72^, including nuclear, chloroplast, and mitochondrial scaffolds. Next, reference gene presence/absence variation was characterized using the SGSGeneLoss package^73^, defining as gene absence when the horizontal coverage across all exons of the gene was <5%.

De novo assembly of unmapped reads was conducted using Velvet as implemented by VelvetOptimizer^74^ with a range of k-mers between 21 and 51 to enable the assembly of contigs from low coverage data. All contigs were aligned to the NCBI protein database and the diatom proteins from the PLAZA Diatoms 1.0 and MMETSP^35^ using DIAMOND in translated DNA mode^75^ (*e*-value < 10e−05, bitscore >= 200, –more-sensitive) to search for regions that potentially contain de novo-assembled dispensable genes. In addition, contigs were aligned to the D6 reference genome using BLASTn v2.7.1^57^ (>= 75% identity and *e*-value < 10e−03) to identify regions that after de novo assembly were already represented in the reference genome. These regions were queried using Bedtools v2.27^76^ (subtract -A) to discard any de novo-assembled gene that overlapped with the reference genome. The resulting genes were screened for contamination by checking the taxonomy of the top five best hits in the DIAMOND search against the NCBI protein database, discarding genes exclusively matching non-eukaryotic genes. The remaining de novo-assembled genes were functionally annotated by transferring the functional annotation of its best hit in the PLAZA Diatoms 1.0 and genes annotated as transposable elements were discarded. To identify redundant de novo-assembled genes, the gene DNA sequences were processed using CD-HIT v4.8.1^77^, with a similarity threshold of 95%, keeping the longest sequence of each cluster and generating a final non-reference dispensable gene data set. The unmapped reads were mapped back to the de novo-assembled contigs that contained this final non-reference gene data set in order to determine the presence/absence variation across all resequencing strains following the previously reported SGSGeneLoss protocol. All these mentioned steps were also applied to determine the presence/absence of genes in the *P. tricornutum* species complex as well as to identify extra de novo-assembled dispensable genes using resequencing data of ten geographically distant *P. tricornutum* strains^32^.

### SNP calling and analysis of gene selection patterns

Picard tool v1.94 was employed to mark duplicate reads in the previously mentioned alignments of the 48 different *S. robusta* strains to the reference D6 genome. SNP calling was executed using Genome Analysis Toolkit (GATK) v3.7-0^78^ according to the GATK best practices. Variants were called per sample using HaplotypeCaller in GVCF mode and then passed together to the GenotypeGVCFs tool to generate a joint-called set of SNPs. The SNPs were further filtered out to generate a high-confidence coding SNP data set as explained in Supplementary Note 6. This high confidence coding SNP data set comprised 28 Mb covering 20,891 genes, and was functionally annotated using snpEff v4.3t^79^. Finally, we calculated the nucleotide diversity at nonsynonymous (π_N_) and synonymous (π_S_) sites and the ratio of these two for this set of genes by computing the polymorphism of the callable coding sequence of each gene, combining this with the functional information of snpEff output and correcting for the allele frequency of the mutation in the population.

### Identification of *S. robusta* signature genes

All *S. robusta* protein-coding genes (core and dispensable) were aligned to the diatom MMETSP^35^ proteins using DIAMOND in translated DNA mode^75^ (–*e*-value 1e-05–min-score 200–max-target-seqs 2000–more-sensitive). These 180 diatom MMETSP samples were classified as pennate/centric, raphid/araphid, and benthic/planktonic species, recovering protein sequences for 32 pennate, 56 centric, 18 raphid, 14 araphid, 8 benthic, and 73 planktonic species in total. For benthic/planktonic classification, we only kept those species with a clearly benthic or planktonic lifestyle, excluding those that were unclear as well as the ice benthic diatoms. For each *S. robusta* gene, we computed a pennate, centric, raphid, araphid, benthic, and planktonic score based on the number of species from each category that the *S. robusta* query gene had a homologous hit. This score ranges from zero to one, where, for example, a pennate score of zero reports the gene has no hits with any pennate species, and a pennate score of one means homologs were found in all pennate species of the MMETSP samples. Next, we calculated the pennate/raphid/benthic signature as follows:

1 $${\mathrm{Pennate}}\,{\mathrm{signature}} = {\mathrm{log}}2\left( {\frac{{{\mathrm{pennate}}\,{\mathrm{score}}}}{{{\mathrm{centric}}\,{\mathrm{score}}}}} \right)$$

2 $${\mathrm{Raphid}}\,{\mathrm{signature}} = {\mathrm{log}}2\left( {\frac{{{\mathrm{raphid}}\,{\mathrm{score}}}}{{{\mathrm{araphid}}\,{\mathrm{score}}}}} \right)$$

3 $${\mathrm{Benthic}}\,{\mathrm{signature}} = {\mathrm{log}}2\left( {\frac{{{\mathrm{benthic}}\,{\mathrm{score}}}}{{{\mathrm{planktonic}}\,{\mathrm{score}}}}} \right).$$

Since we are working with transcriptomes, note we cannot irrefutably affirm the absence of a certain gene in a diatom clade. As a consequence, to define genes with high pennate or raphid signature we used a signature threshold > = 3 but also verified if the genomic data support the observed signal, i.e., the diatom homologs of the family of the query gene were all from pennate and/or raphid diatoms. In contrast, as we only have genomic data from one true benthic diatom species (*S. robusta*), genes with high benthic signature were defined as having a signature threshold > = 3 and a minimum of two benthic species in the MMETSP samples. Enrichment analysis of genes with a specific high signature was performed using the previously described enrichment analysis (*q*-value cutoff of 0.05 and minimum of two hits).

To evaluate how gene signature values change when using different cutoffs to identify homologs in the MMETSP data set, we performed a control experiment using a more relaxed threshold (–*e*-value 1e-03–min-score 0). This analysis revealed that 81%, 73%, and 97% of the genes with high pennate, raphid and benthic signature had the same positive signature trend using both the stringent and relaxed cutoff, and that the use of these more relaxed thresholds leads to the recovery of more distantly related homologs that do not strongly change the detected signature (Supplementary Fig. 38A–C).

### Reporting summary

Further information on research design is available in the Nature Research Reporting Summary linked to this article.

## Supplementary information

Supplementary information

Peer Review

Reporting Summary

## Data Availability

Data supporting the findings of this work are available within the paper and its Supplementary Information files. A reporting summary for this article is available as a Supplementary Information file. The data sets generated and analyzed during this study are available from the corresponding author upon request. The genome assembly and gene model annotation can be downloaded from the ORCAE platform [https://bioinformatics.psb.ugent.be/orcae/overview/Semro]. DNA-sequencing data generated from this study have been deposited in the European Nucleotide Archive with the accession code of PRJEB36614. The fastq files representing the raw RNA sequencing data have been deposited in the EBI Array Express under accession number E-MTAB-8685. Source for comparative genomics analysis of this study is accessible in the PLAZA Diatoms 1.0 [https://bioinformatics.psb.ugent.be/plaza/versions/plaza_diatoms_01/], which includes differential gene expression information for all *S. robusta* genes. The source data underlying Figs. 2, 3, 4a, b, and 5, and Supplementary Figs. 10, 13–17, 20, 22, and 29, as well as Supplementary Table 10 are provided as a Source Data file. Source data are provided with this paper.

## Source data

Source Data
