# Author Correction: The *Seminavis robusta* genome provides insights into the evolutionary adaptations of benthic diatoms

**DOI:** 10.1038/s41467-020-19222-w

**Published:** 2020-10-16

**Authors:** Cristina Maria Osuna-Cruz, Gust Bilcke, Emmelien Vancaester, Sam De Decker, Atle M. Bones, Per Winge, Nicole Poulsen, Petra Bulankova, Bram Verhelst, Sien Audoor, Darja Belisova, Aikaterini Pargana, Monia Russo, Frederike Stock, Emilio Cirri, Tore Brembu, Georg Pohnert, Gwenael Piganeau, Maria Immacolata Ferrante, Thomas Mock, Lieven Sterck, Koen Sabbe, Lieven De Veylder, Wim Vyverman, Klaas Vandepoele

**Affiliations:** 1 Department of Plant Biotechnology and Bioinformatics, Ghent University, Technologiepark 71, 9052 Ghent, Belgium; 2 VIB Center for Plant Systems Biology, Technologiepark 71, 9052 Ghent, Belgium; 3 Bioinformatics Institute Ghent, Ghent University, Technologiepark 71, 9052 Ghent, Belgium; 4 Protistology and Aquatic Ecology, Department of Biology, Ghent University, 9000 Ghent, Belgium; 5 Department of Applied Mathematics, Computer Science and Statistics, Ghent University, 9000 Ghent, Belgium; 6 Cell Molecular Biology and Genomics Group, Department of Biology, Norwegian University of Science and Technology, 7491 Trondheim, Norway; 7 B CUBE Center for Molecular Bioengineering, Technical University of Dresden, Tatzberg 41, 01307 Dresden, Germany; 8 Integrative Marine Ecology, Stazione Zoologica Anton Dohrn, Villa Comunale, Naples, Italy; 9 Friedrich Schiller University Jena, Institute of Inorganic and Analytical Chemistry, Lessingstrasse 8, 07745 Jena, Germany; 10 Sorbonne Université, CNRS, UMR 7232 Biologie Intégrative des Organismes Marins BIOM, Observatoire Océanologique, F-66650 Banyuls-sur-Mer, France; 11 School of Environmental Sciences, University of East Anglia, Norwich Research Park, Norwich, NR4 7TJ UK

**Keywords:** Comparative genomics, Molecular evolution, Genetic variation

Correction to: *Nature Communications* 10.1038/s41467-020-17191-8, published online 03 July 2020.

In the original version of this Article, under the Methods subsection “DNA extraction and genome size estimation”, the availability of the benthic diatoms used in this study and the accession number of the genome-sequenced strain were omitted. The first sentence, “Both Illumina and PacBio technologies were used for sequencing of *S. robusta* D6 strain mating type plus (MT+) to take advantage of short-read (better quality) and long-read (better contiguity) sequencing approaches”, should be replaced by “The *S. robusta* D6 reference strain (accession number DCG 0498) and the 48 additional strains used in this study are available from the BCCM/DCG diatom culture collection at Ghent University (http://bccm.belspo.be/about-us/bccm-dcg). Both Illumina and PacBio technologies were used for sequencing of the D6 strain (mating type plus (MT+)) to take advantage of short-read (better quality) and long-read (better contiguity) sequencing approaches”. This has been corrected in both the PDF and HTML versions of the Article.

