# Supplementary material for: The Seminavis robusta genome provides insights into the evolutionary adaptations of benthic diatoms
Source: Nat Commun. 2020 Jul 3;11:3320. doi: 10.1038/s41467-020-17191-8 (PMC7335047; doi:10.1038/s41467-020-17191-8)
Supplement: Supplementary file 1 — Supplementary information [file 41467_2020_17191_MOESM1_ESM.pdf]

The *Seminavis robusta* genome provides insights into the evolutionary adaptations of benthic diatoms

Osuna-Cruz *et al.*

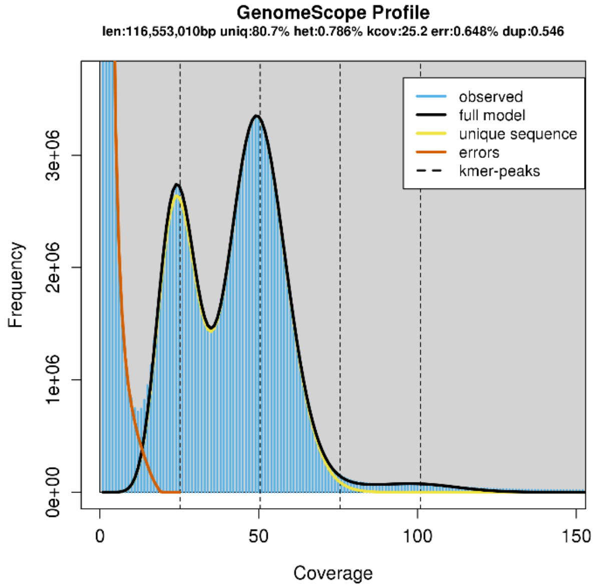

Supplementary Figure 1. 31-mer frequency distribution of the *S. robusta* genome Illumina sequence reads. The k-mer distribution was calculated using Jellyfish v2.2.6 [1] (k = 31) and the resulting histogram was uploaded to GenomeScope v1 [2]. The first lower-frequency peak corresponds to k-mers present on one haplotype (heterozygous regions), whereas the second higher-frequency peak corresponds to k-mers present on both haplotypes (homozygous regions).

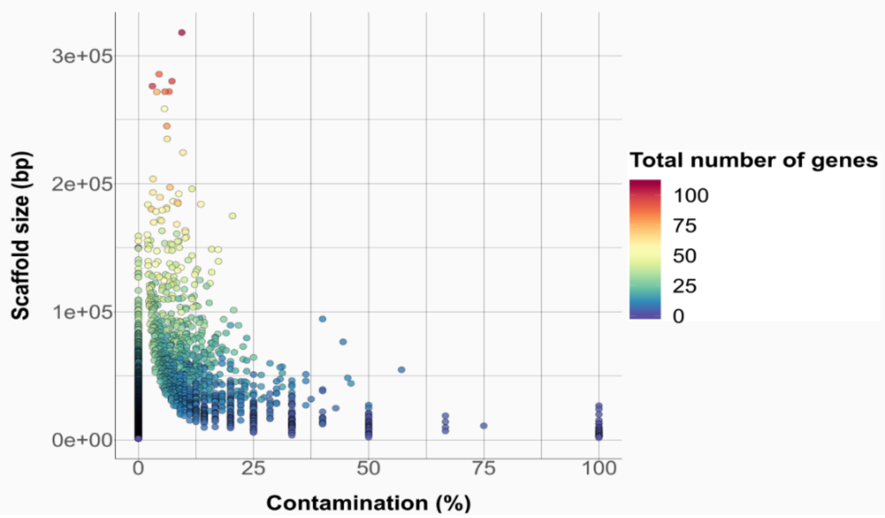

Supplementary Figure 2. Scatter plot of scaffold contamination content prior removal of contaminants. The possible contamination in the genome was addressed by the taxonomic assignment of 22,224 protein-coding genes based on the top 5 best hits of a Tera-BLASTX v7.6.1 [3] search against the NCBI protein database (e-value < 10e-05). Every dot represents a scaffold, x-axis shows percentage of contaminant genes (Bacteria, Fungi or Archaea taxonomy) whereas y-axis represents the scaffold size. Scaffolds are colored according to the number of genes they contain, in a rainbow scale, from blue to red. This analysis revealed that *S. robusta* scaffolds were minimally contaminated by bacterial sequences since only 33 scaffolds had a contamination level higher than 50%. These scaffolds were manually inspected and only four scaffolds were removed from the assembly as true contaminants since these contain genes without strong expression evidence and coming from the same contaminant species (e.g. *Escherichia coli*). Secondly, a BLASTN sequence similarity search (identity >70% and coverage >25%) against the NCBI nucleotide database was performed to identify scaffolds that could still represent potential contamination. Apart from the scaffolds already removed, no additional scaffolds were identified as contaminant.

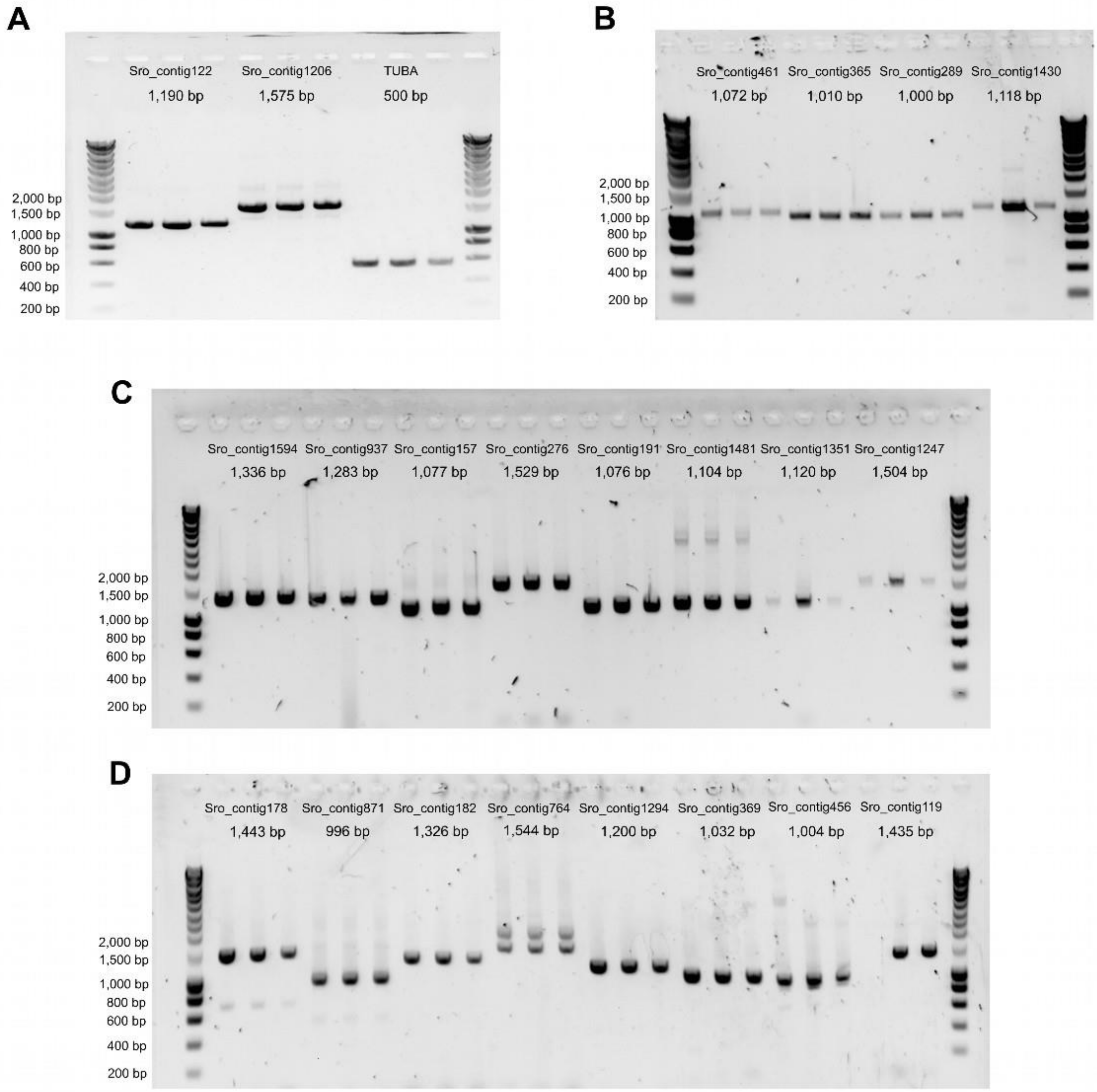

Supplementary Figure 3. Gels from the experimental validation of the *S. robusta* genome assembly. From panel A–D, twenty-two regions amplified by PCR are shown. There are three lanes from different independent PCR runs for each amplified region (n = 3), reporting the expected product size on the upper part. More details about these amplified regions, their coordinates, primers and exact product size obtained are given in Supplementary Note 2 and Supplementary Table 3.

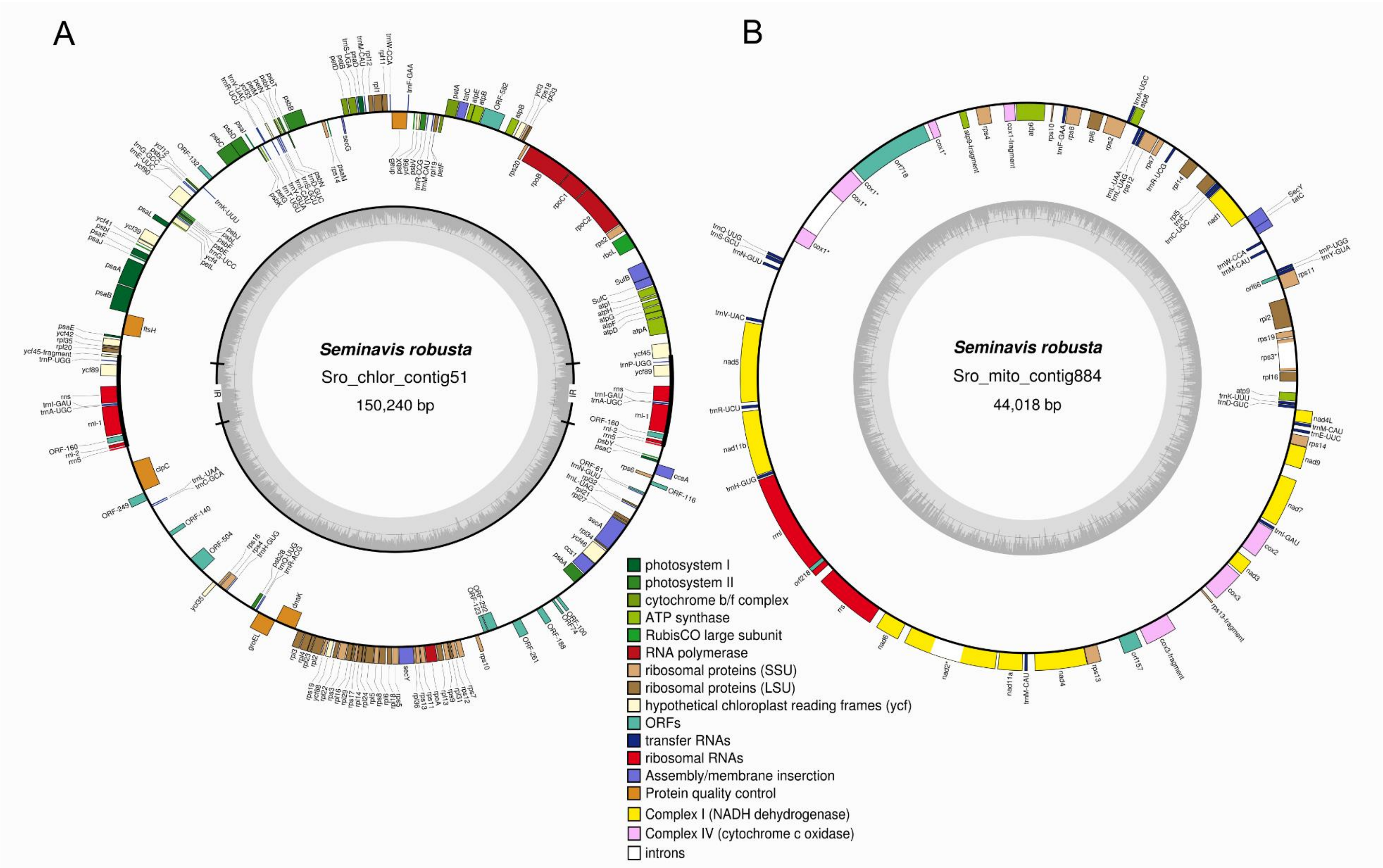

Supplementary Figure 4. Overview of the *S. robusta* chloroplast and mitochondrial annotated genomes. A BLASTn (>95% identity) was executed to search for similarity with the already published *S. robusta* chloroplast genome [4] and the published mitochondrial genomes of several closely related species (*Phaeodactylum tricornutum* [5], *Thalassiosira pseudonana* [5] and *Navicula ramosissima* [6]) in the final genome assembly. These searches indicated that Sro_chlor_contig51 and Sro_mito_contig884 are the chloroplast and mitochondrial genome, respectively. Next, GeSeq [7] was employed to perform organelle genome annotation and manual curation was done based on gene homology information from other diatom chloroplast/mitochondrial genomes. GeSeq was run with default parameters, except the maximum intron size in tRNAscan-SE, which was set up to 6,000. The final resulting chloroplast (A) and mitochondrial (B) genomes were uploaded to OGDraw v1.3.1 [8] for data visualization.

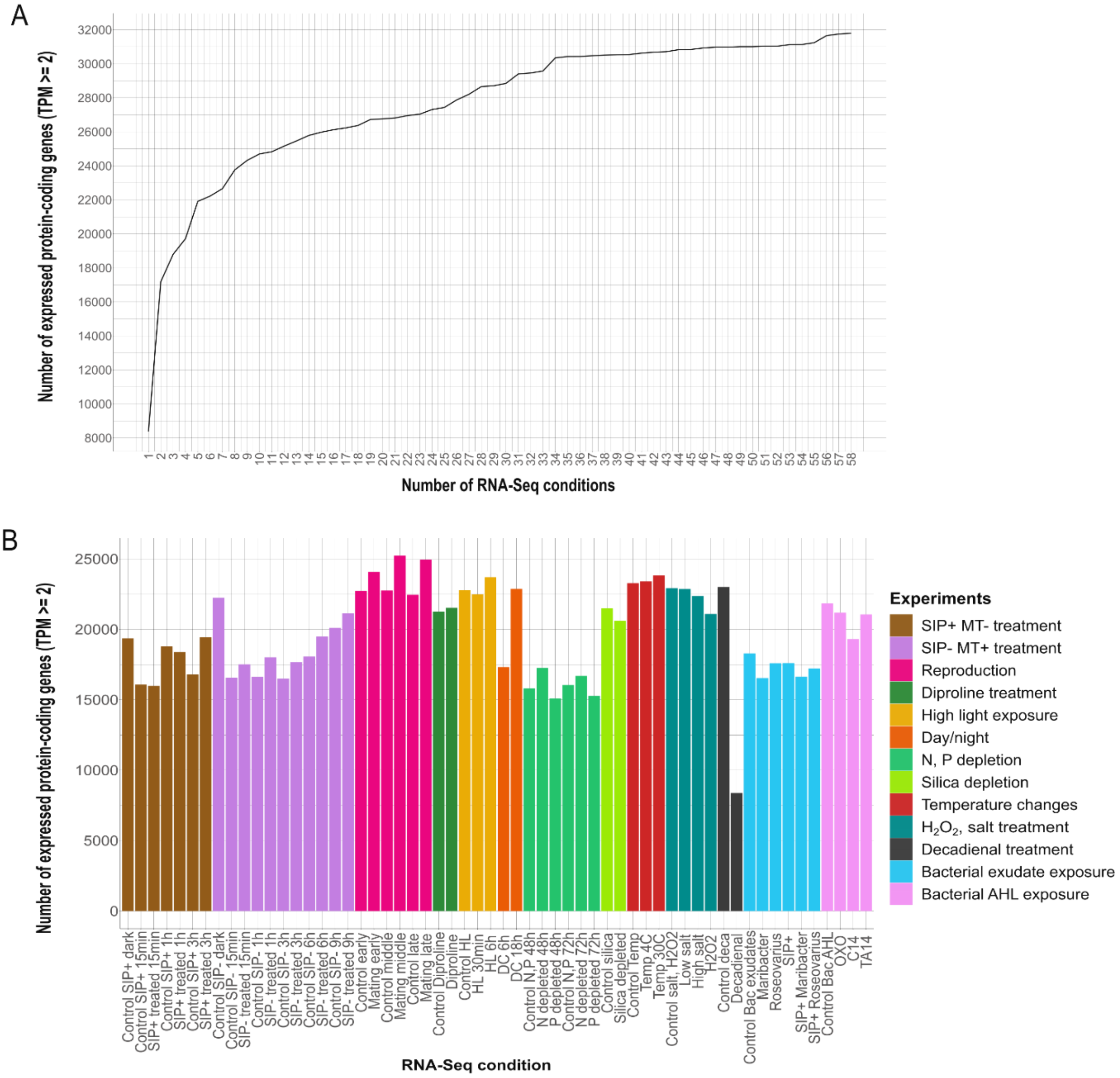

Supplementary Figure 5. Overview of profiled conditions for *S. robusta* expression atlas and expressed protein-coding genes (TPM >= 2). (A) Cumulative curve showing the total number of detected expressed protein-coding genes across all conditions (control + treatment). **(B)** Number of expressed protein-coding genes per condition (control + treatment), including sexual reproduction, abiotic stress and bacteria-interaction experiments. TPM refers to Transcripts Per Million.

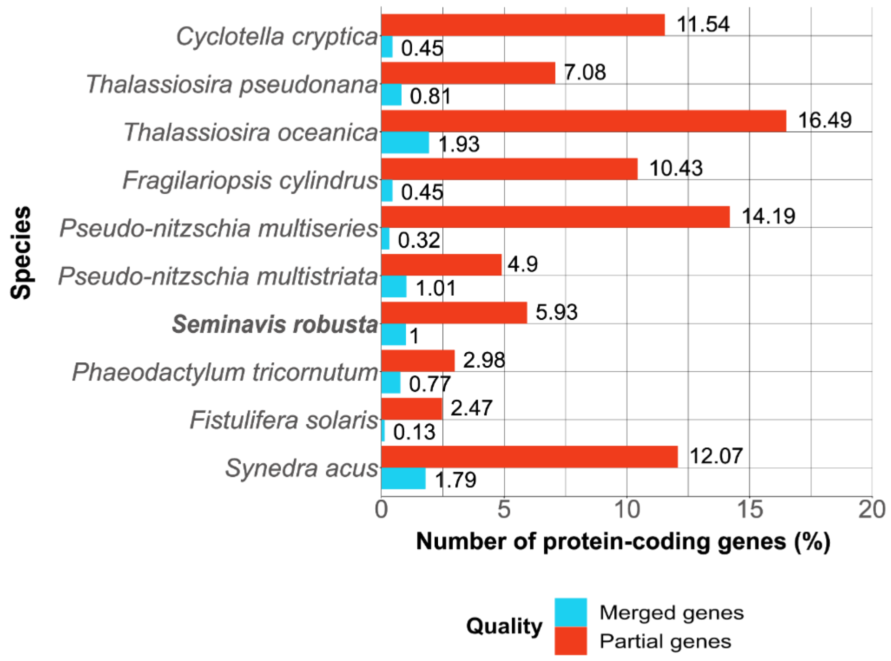

Supplementary Figure 6. Quality of diatom gene models measured as percentage of potential partial (blue) and merged (red) protein-coding genes. More information can be found in Supplementary Note 2.

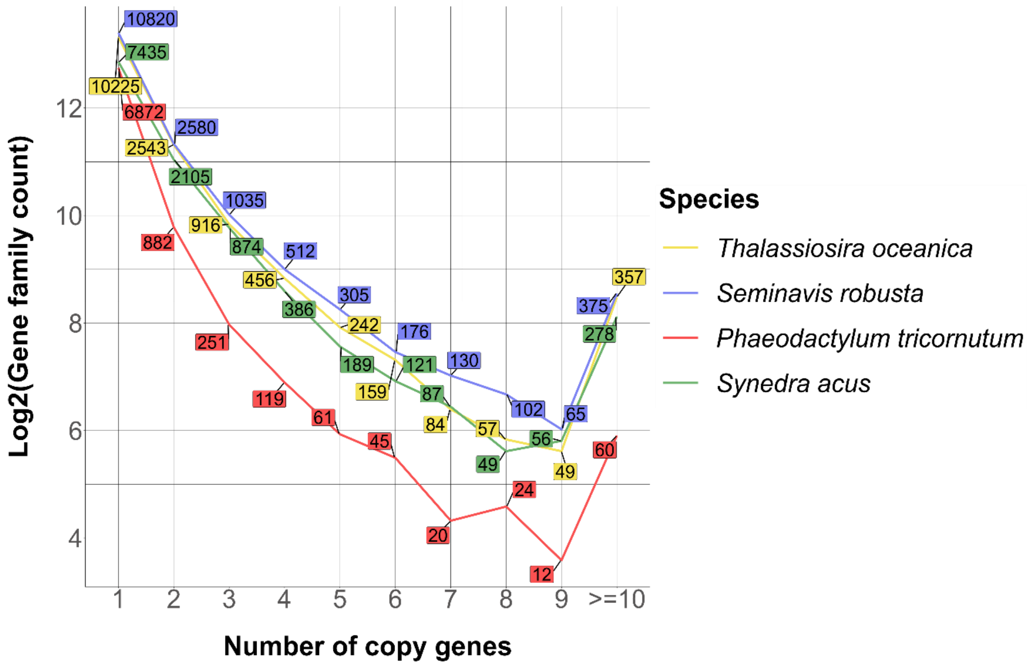

Supplementary Figure 7. Gene family size distribution of *S. robusta*, *T. oceanica, S. acus* and *P. tricornutum* species. *S. robusta* has the largest number of gene families with more than 10 copy genes. More information can be found in Supplementary Note 3.

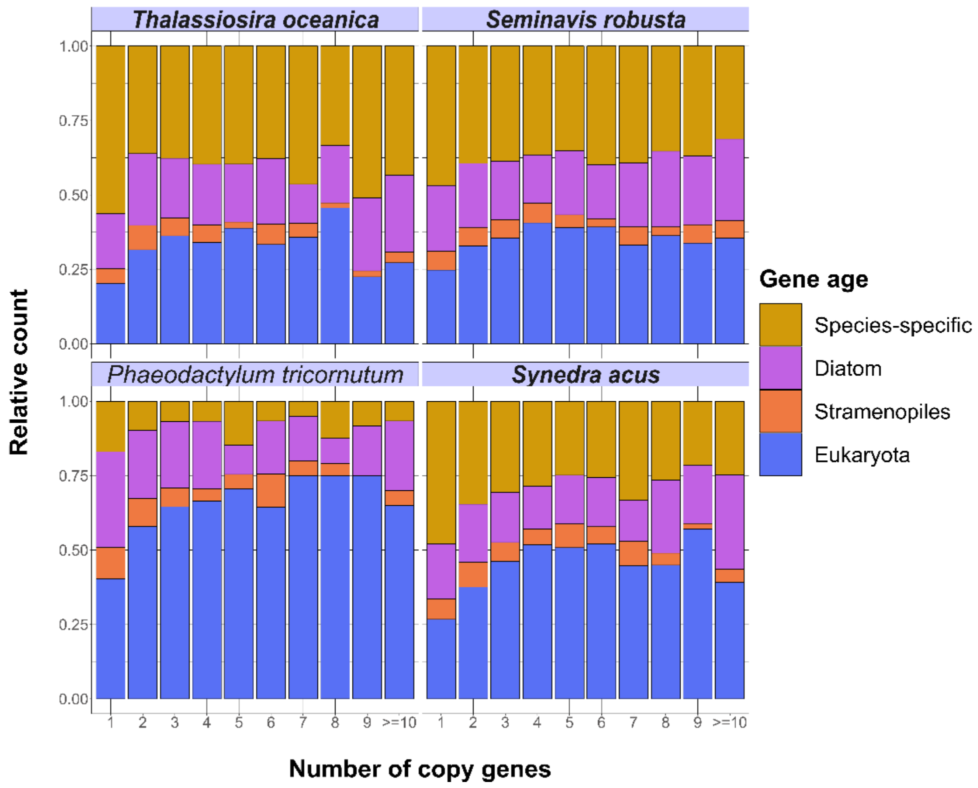

Supplementary Figure 8. Gene family size and gene age proportions for *S. robusta*, *T. oceanica, S. acus* and *P. tricornutum* species. Diatoms with a genome size > 90 Mb are highlighted in bold. More information can be found in Supplementary Note 3.

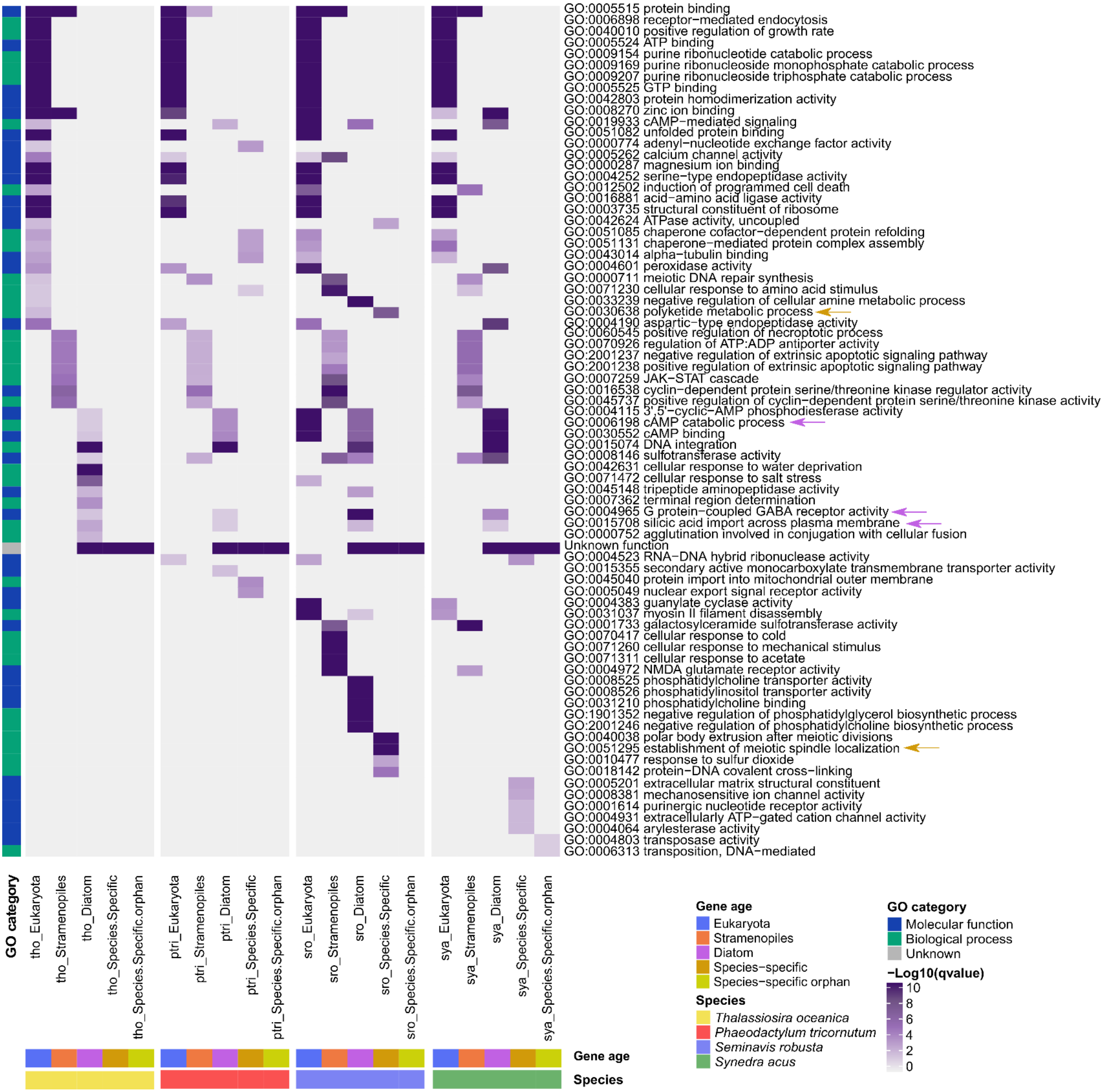

Supplementary Figure 9. A selection of enriched GO terms for each gene age category for *S. robusta*, *T. oceanica*, *S. acus* and *P. tricornutum*. GO terms were predicted by running InterPro2GO [9] and eggNOG-mapper v1 [10]. GO enrichment analysis per age category was performed using hypergeometric distribution with q-value cutoff of 0.05 and minimum of two hits. More information can be found in Supplementary Note 3.

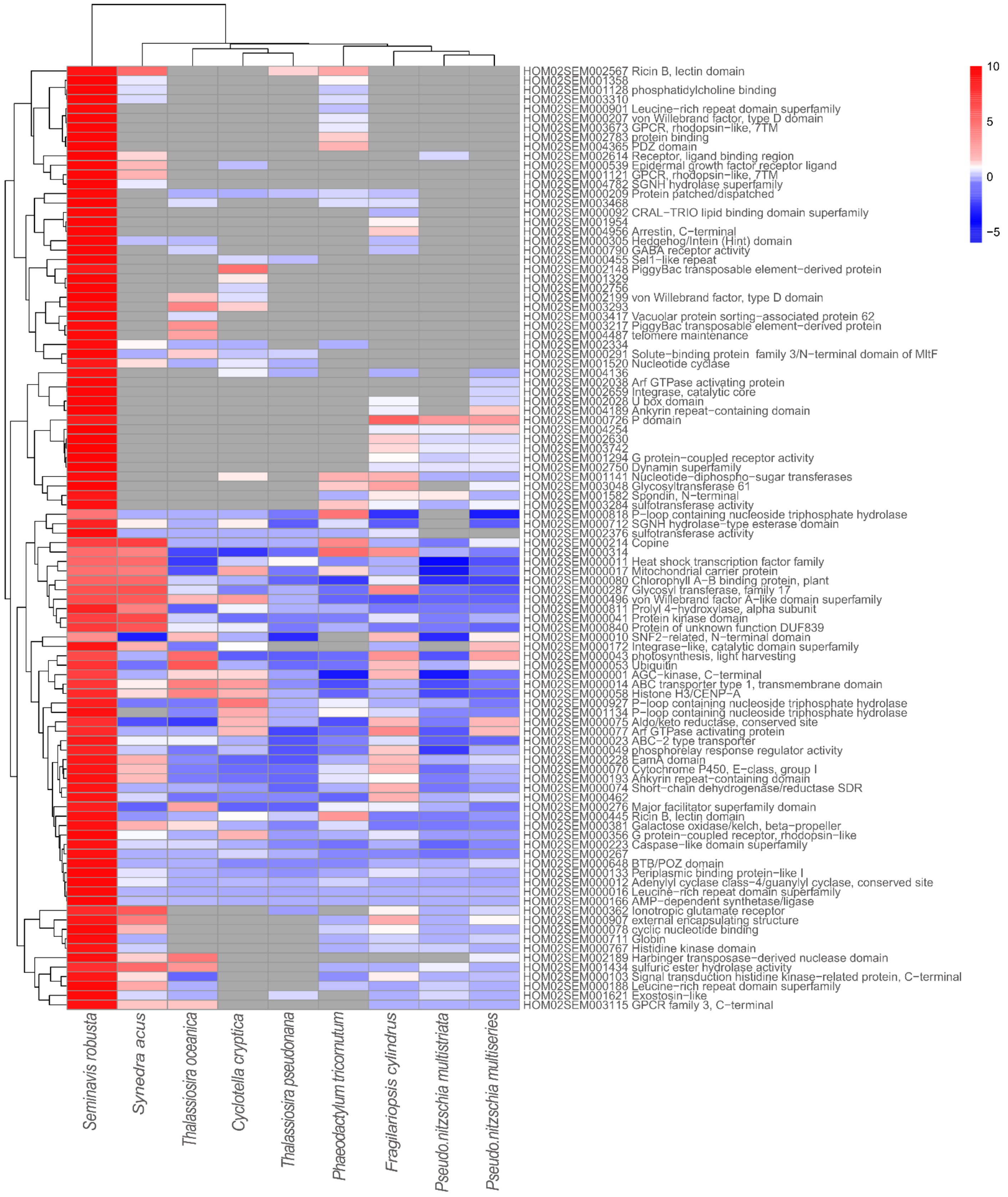

Supplementary Figure 10. Top 100 most expanded *S. robusta* gene families. Z-scores of the median gene copy number of each gene family for each species are shown in a color gradient from blue (depleted) to white (equal to the median) to red (expanded). Absence of a gene family for a species is shown as dark grey. The gene composition of each gene family can be found in PLAZA Diatoms 1.0 (https://bioinformatics.psb.ugent.be/plaza/versions/plaza_diatoms_01/). Source data are provided as a Source Data file.

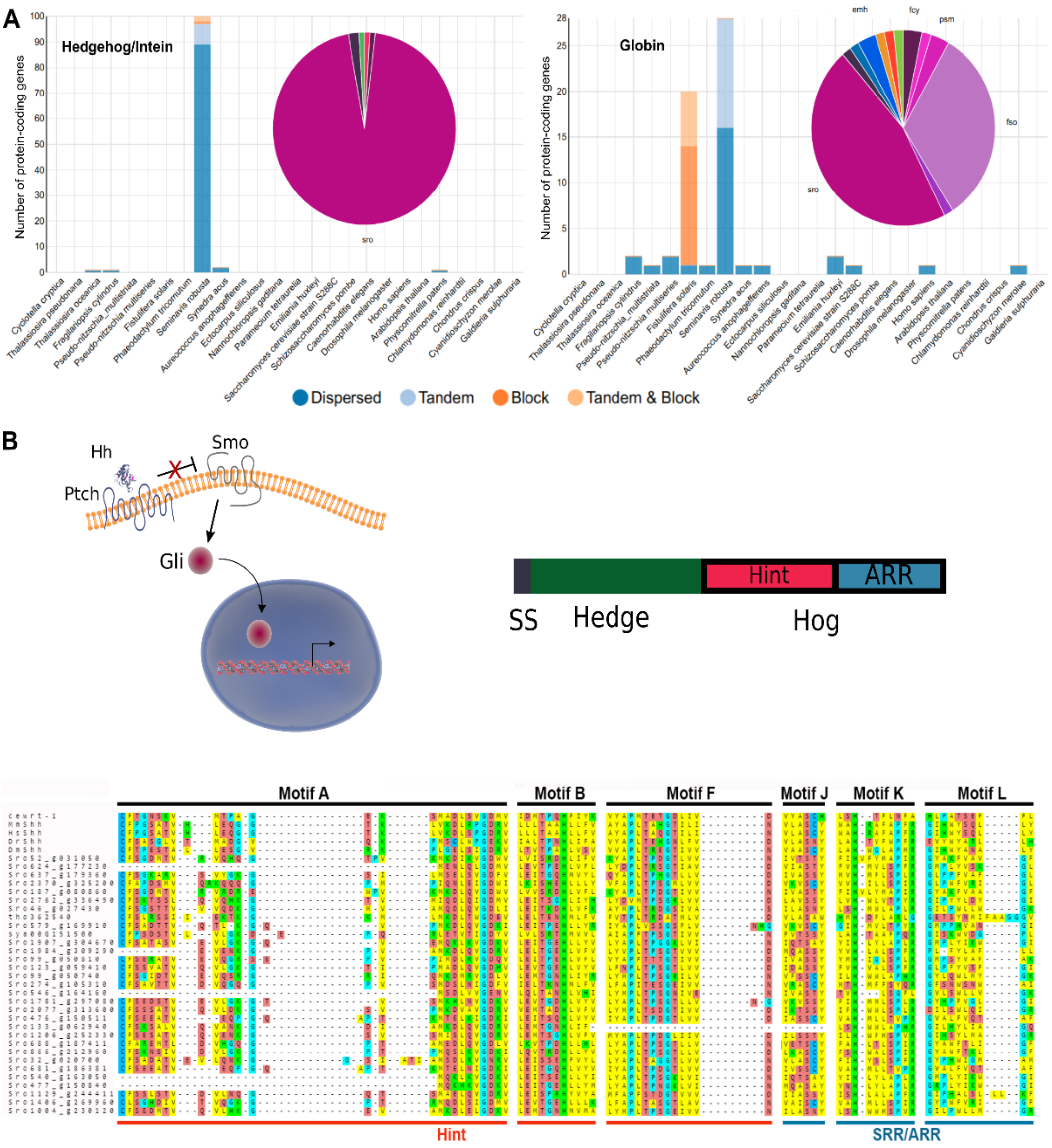

Supplementary Figure 11. Examples of species-specific *S. robusta* expanded families driven by different duplication mechanisms. (A) Screenshots from the PLAZA Diatoms 1.0 platform showing species distribution and duplication type for Hedgehog/Intein (*HOM02SEM000305*) and Globin (*HOM02SEM000711*) families. This globin family is discussed in more detail in the main text. **(B)** Top left, a simplified scheme of the Hh signaling pathway: when the Hh ligand binds to its receptor Patched (Ptch), the inhibition of Ptch on Smoothened (Smo) is relieved, this causes the translocation of the transcription factor Gli from the cytoplasm to the nucleus where it will regulate transcription of target genes. Ptch genes exist in the *S. robusta* genome (families *HOM02SEM011911* and *HOM02SEM000209*), and families *HOM02SEM003604* and *HOM02SEM003629* contain genes annotated as Dispatched, a protein required for Hh secretion, but none of the other major components of the Hh pathway are present, an indication that the pathway is incomplete in *S. robusta*. Top right, schematic representation of the Hh protein with the signal peptide for secretion (SS, gray), the N-terminal hedge domain that is secreted and acts as ligand (green) and the C-terminal hog domain that has self-processing activity (black), with its two modules Hint (related to inteins, widely conserved self-splicing proteins) and ARR (adduct recognition region). All the HOM02SEM000305 diatom proteins lack the N-terminal portion, while they retain the C-terminal Hog domain, consistently with the knowledge that Hh-related proteins in unicellular organisms only contain a Hog domain, and that the Hedge sequences seem to be unique to Metazoa [11]. Hog proteins often have a signal peptide indicating secretion, such a feature is observed in 74 out of 105 members of the HOM02SEM000305 family, as assessed by SignalP [12]. Bottom, the different conserved motifs of the Hog domain all appear to be present in diatoms. Alignment of representative members of the HOM02SEM000305 diatom proteins and *Caenorhabditidis elegans warthog* (Ce_wrt-1), *Mus musculus* (Mm_Shh), *Homo sapiens* (Hs_Shh), *Danio rerio* (Dr_Shh) and *Drosophila melanogaster* (Dm_Shh) hedgehog proteins [11] (only the motifs are shown). *S. robusta* genes in this family display a wide variety of expression profiles, making it difficult to infer a specific function.

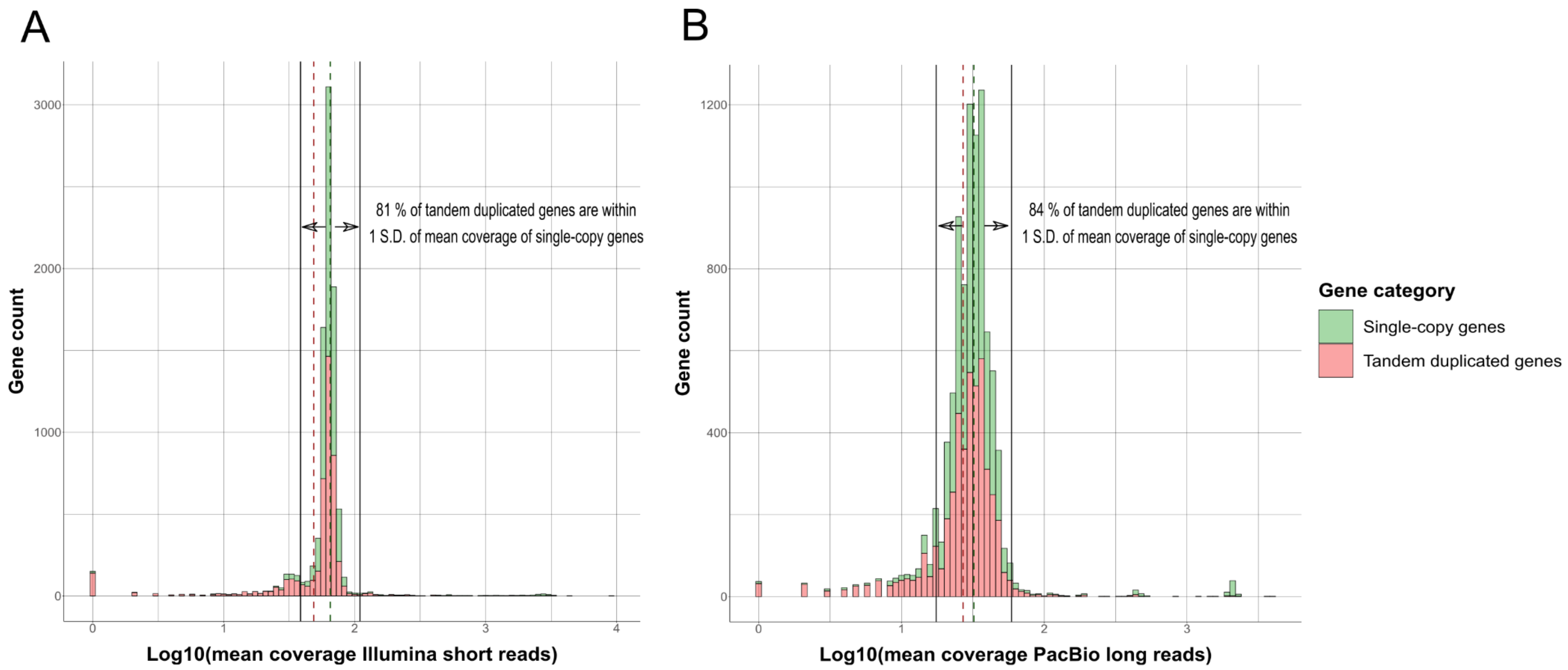

Supplementary Figure 12. Distribution of Illumina and PacBio read mean coverage for single-copy genes (green) and tandem duplicated genes (red). Illumina and PacBio reads were uniquely mapped to the assembly using BWA-MEM v0.7.5a [13] (default parameters) and BLASR v5.3.2 [14] (-bestn 2 -maxAnchorsPerPosition 100 -advanceExactMatches 10 -affineAlign -affineOpen 100 -affineExtend 0 -insertion 5 -deletion 5 -extend -maxExtendDropoff 20), respectively and BEDTools v2.26.0 [15] was executed to compute the coverage per nucleotide position and the mean coverage per gene. First, the standard deviation of the mean read coverage for a random subset of single-copy genes of the same size as the tandem gene duplicate dataset was calculated. Next, the percentage of tandem gene duplicates whose mean read coverage was within one standard deviation of the single-copy gene mean read coverage was determined, yielding 81% in case of Illumina **(A)** and 84% in case of PacBio **(B)**. The average mean read coverage is indicated by dashed lines whereas the region that covers one standard deviation of the mean read coverage of single-copy genes is highlighted by arrows.

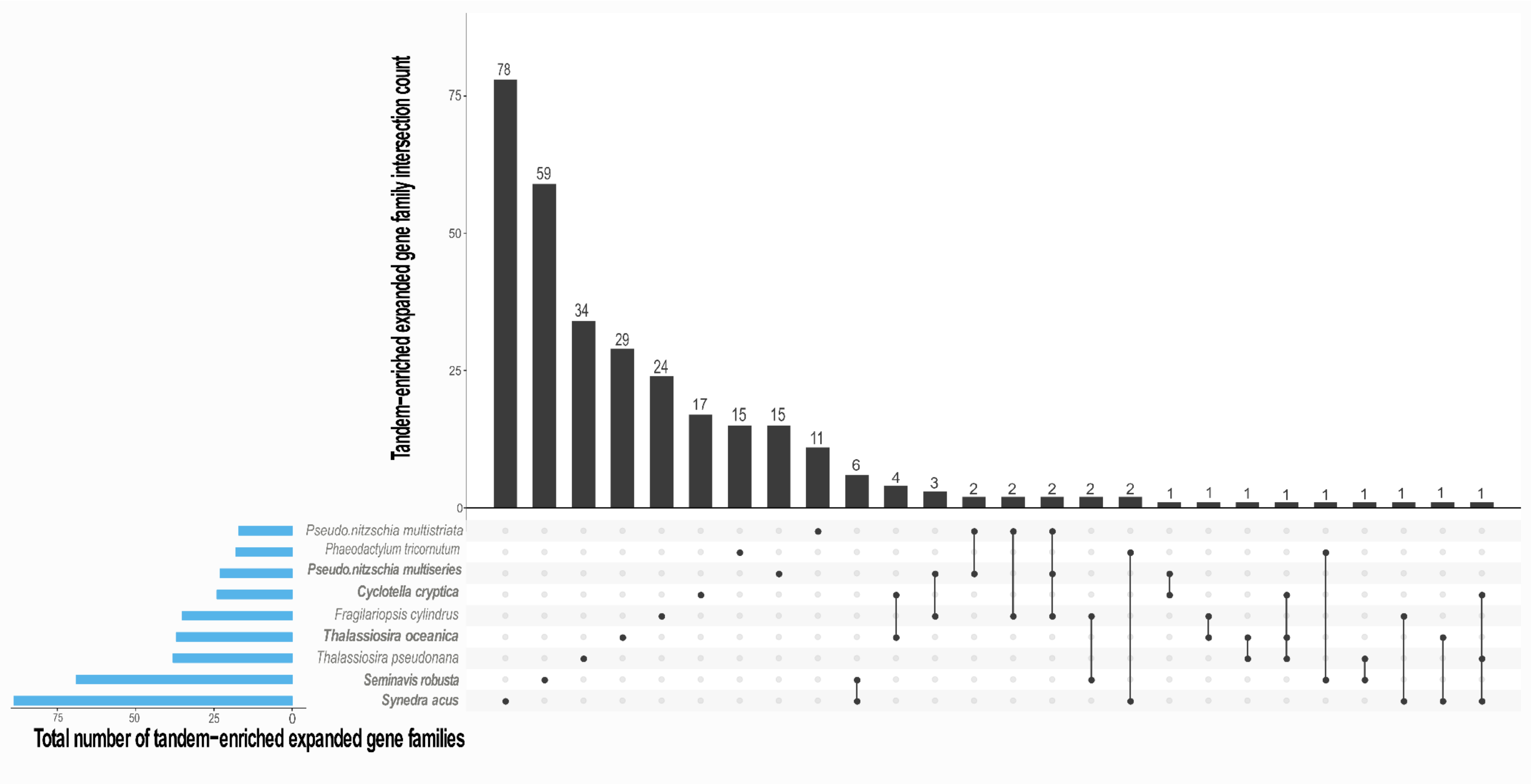

Supplementary Figure 13. Upset plot showing the intersection of tandem-enriched gene family expansions in diatoms. Each row represents a diatom species, displaying the total number of tandem-enriched expanded gene families for each diatom on the left histogram. The main bar plot indicates the total gene family count in each species intersection. Diatoms with a genome size > 90 Mb are highlighted in bold. Source data are provided as a Source Data file.

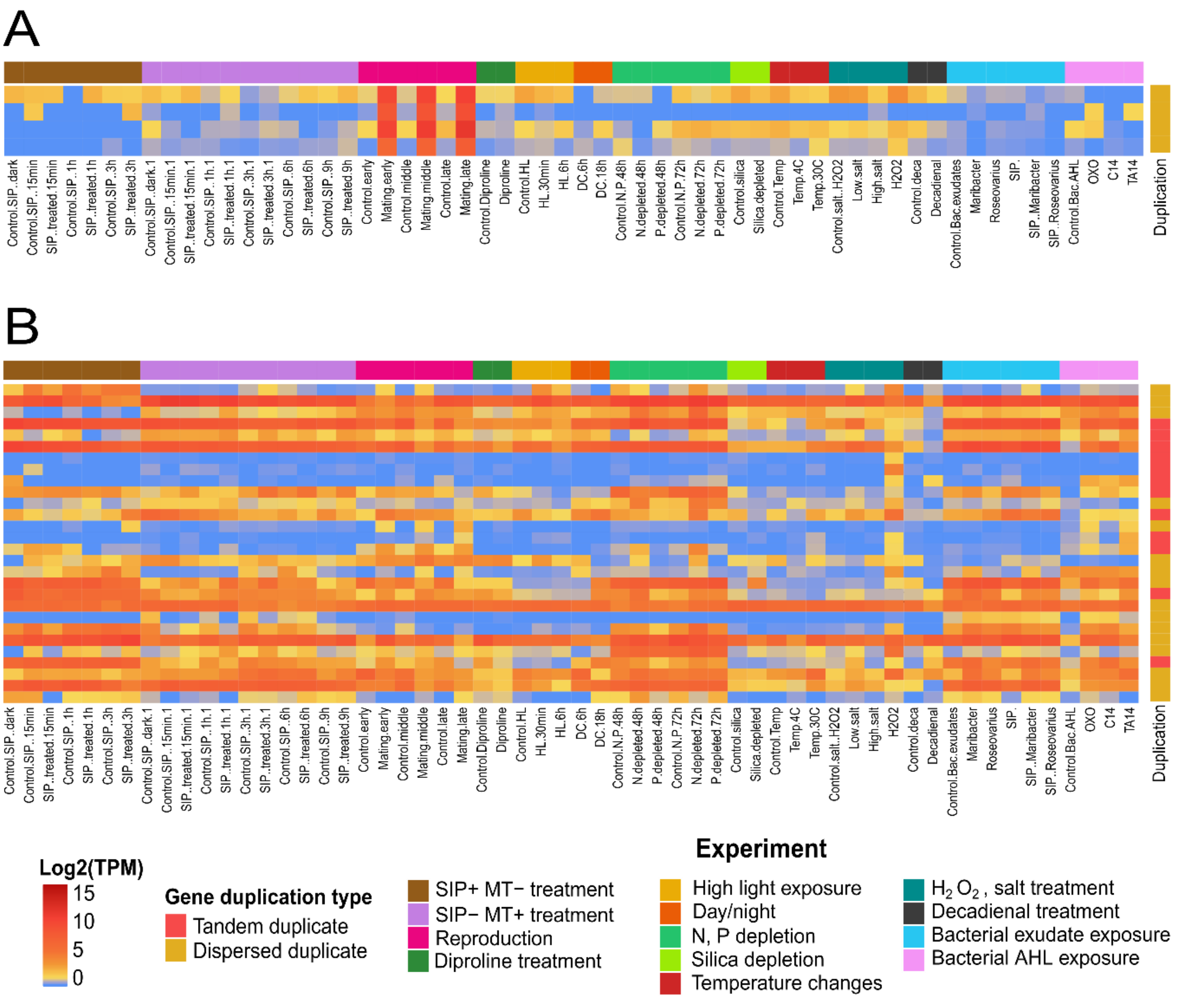

Supplementary Figure 14. Examples of families showing expression conservation (A) and expression divergence (B). **(A)** A small species-specific family with four copies in *S. robusta* (*HOM02SEM021021*) with unknown function showing strong expression conservation. This family has significant expression bias towards reproduction experiments. **(B)** A large eukaryota family expanded and tandem-enriched with 28 copies in *S. robusta* (*HOM02SEM000711*) annotated as globins and showing expression divergence. This family has a pleiotropic expression profile. Source data are provided as a Source Data file.

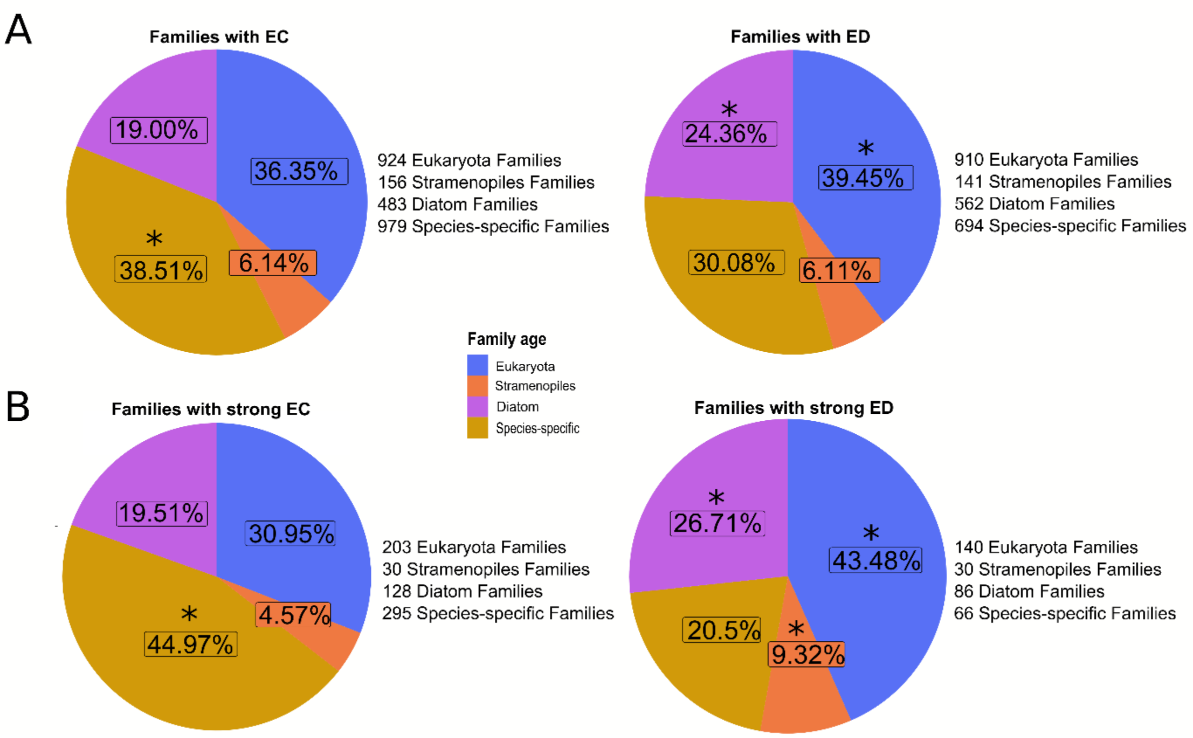

Supplementary Figure 15. Distribution of the age of families showing expression conservation (EC), expression divergence (ED), strong EC and strong ED. The ED of gene duplicates for families having a phylogenetic tree was computed by calculating the Pearson Correlation Coefficient (PCC) of all TPM values between gene duplicates for each node of the tree. If a node contained more than two gene duplicates, the average PCC of the duplication events of that node was taken [16]. Families with only two *S. robusta* genes and no phylogenetic tree were also included in the analysis by simply computing the PCC between the two gene copies. In total, this analysis covered 4,444 families (17,493 genes). The mean PCC of all studied nodes was 0.3 (corresponding with a Z score of zero), defining a node as showing EC when PCC >= 0.3 and ED when PCC < 0.3. A node was considered to display strong EC when the Z-score of the PCC was >= 1.65 and strong ED when <= -1.65. **(A)** Families having >50% of its tree nodes showing EC were considered to show EC (left) whereas families having >50% of its tree nodes showing ED were considered to show ED (right). **(B)** Likewise, families having tree nodes with strong EC but no nodes with strong ED were considered to show strong EC (left) whereas families having nodes with strong ED but no nodes with strong EC were considered to show strong ED (right). Significant age enrichments are highlighted by a star (q-value < 0.05, hypergeometric distribution). We confirmed the observed patterns are not caused by strong differences in family size for different age classes. Source data are provided as a Source Data file.

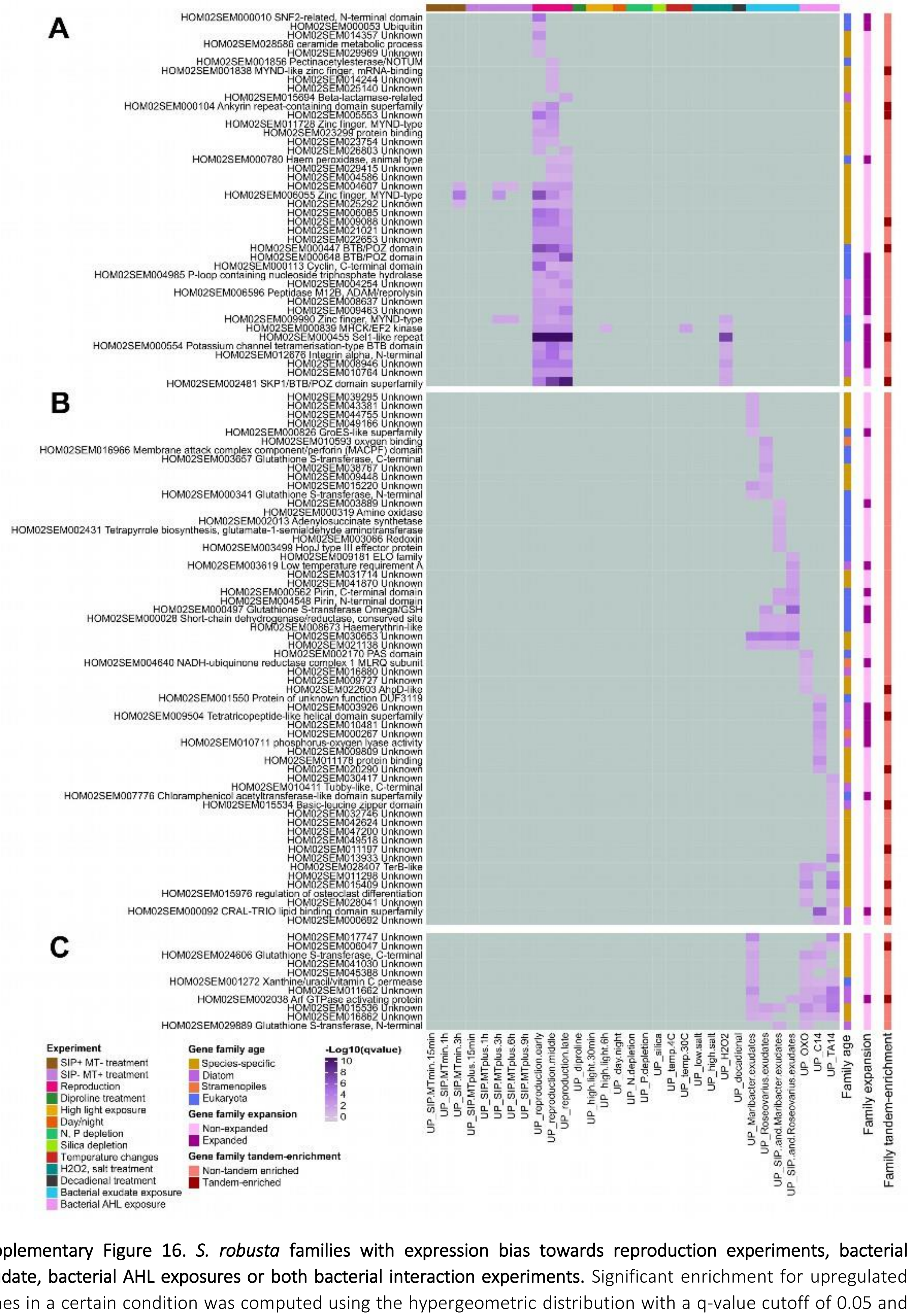

Supplementary Figure 16. ***S. robusta*** **families with expression bias towards reproduction experiments, bacterial exudate, bacterial AHL exposures or both bacterial interaction experiments.** Significant enrichment for upregulated genes in a certain condition was computed using the hypergeometric distribution with a q-value cutoff of 0.05 and minimum of two hits. Panel **(A)** shows families with expression bias towards reproduction, **(B)** towards bacterial exudate, bacterial AHL exposure and **(C)** towards both bacterial interaction experiments. Source data are provided as a Source Data file.

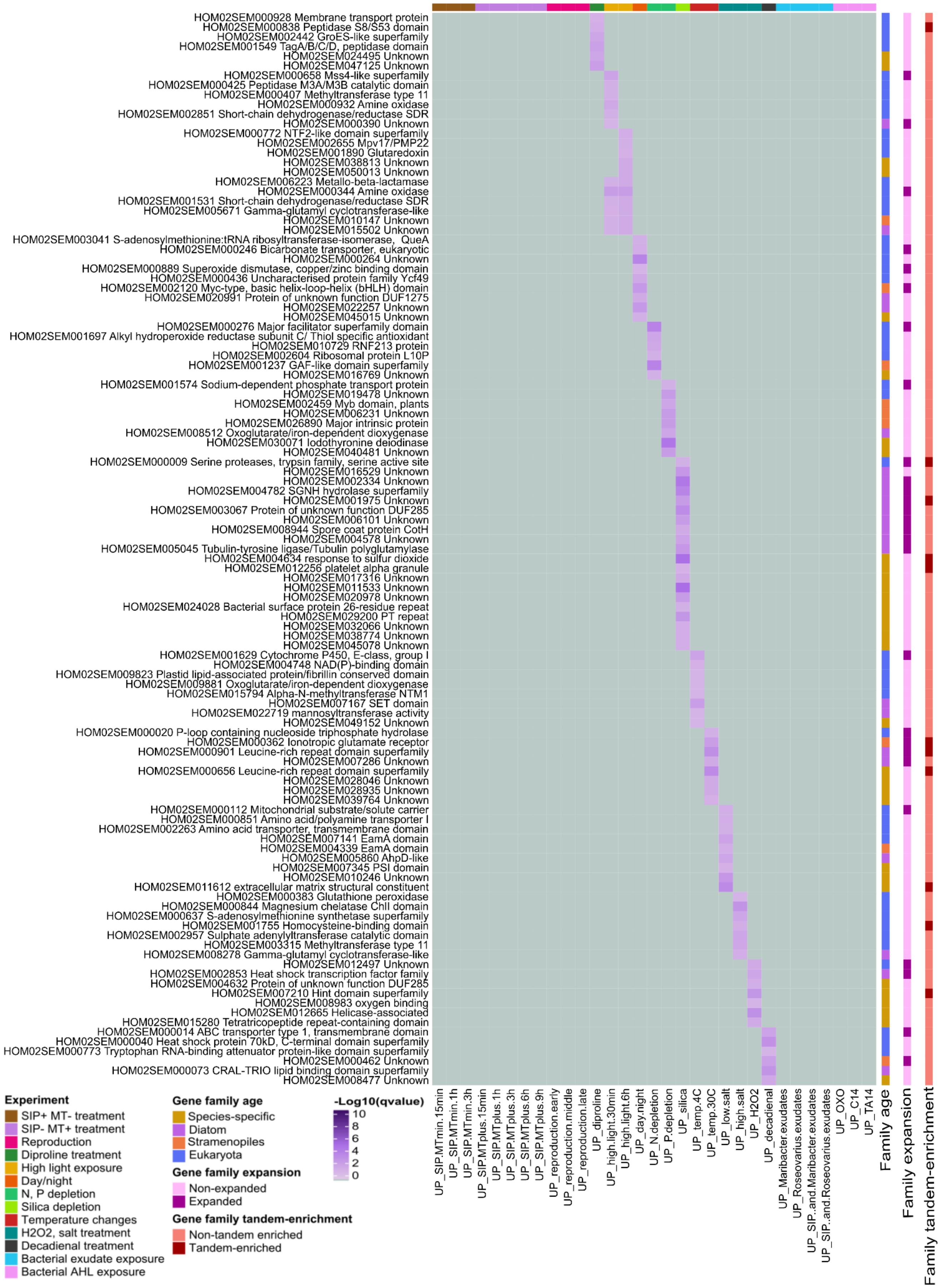

Supplementary Figure 17. *S. robusta* families with expression bias towards abiotic stresses. Significant enrichment for upregulated genes in a certain condition was computed using the hypergeometric distribution with a q-value cutoff of 0.05 and minimum of two hits. Source data are provided as a Source Data file.

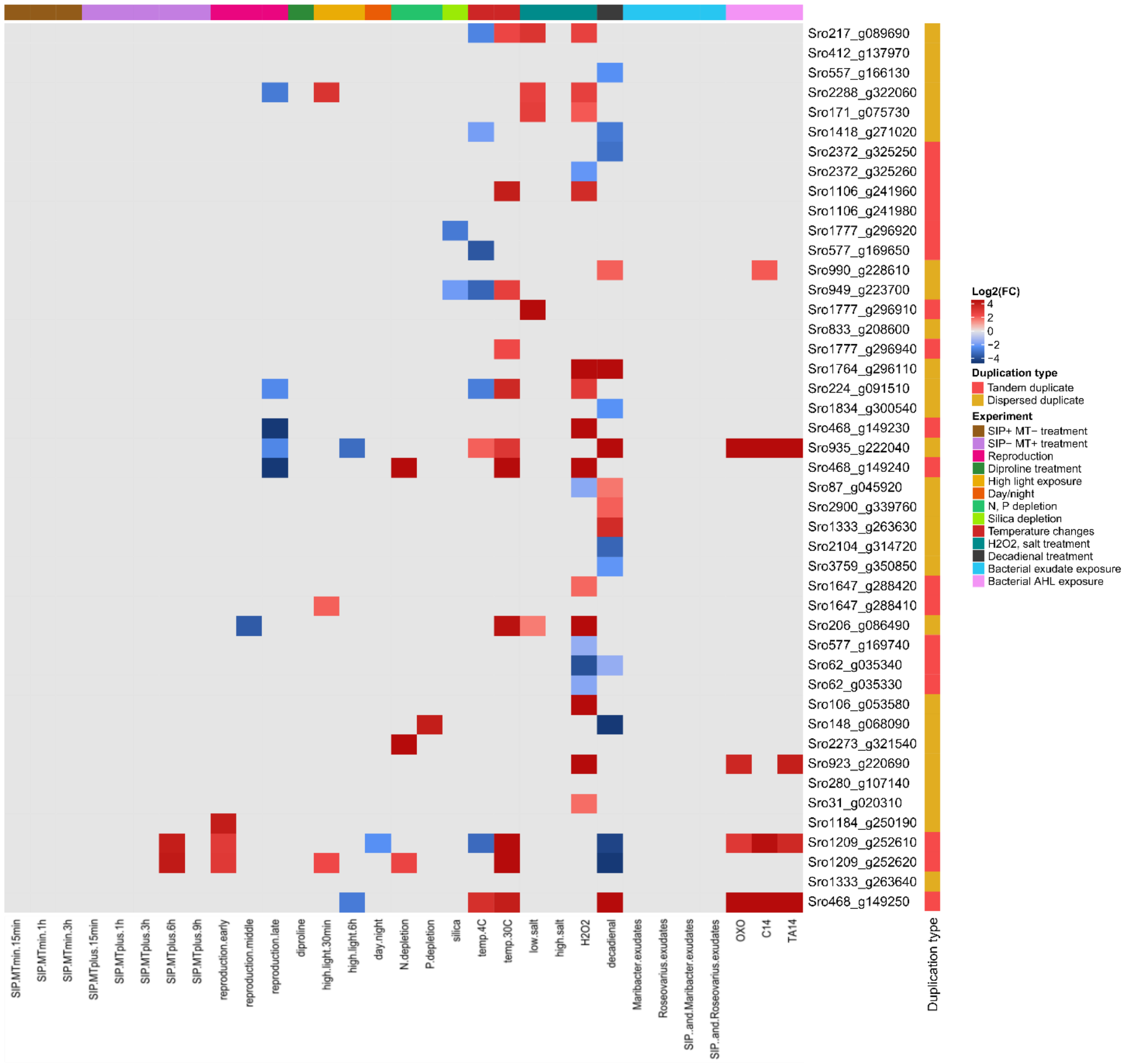

Supplementary Figure 18. Differential expression heatmap for iGluRs family (*HOM02SEM000362*). Tandem duplicated genes are highlighted in red whereas dispersed duplicated genes are highlighted in yellow.

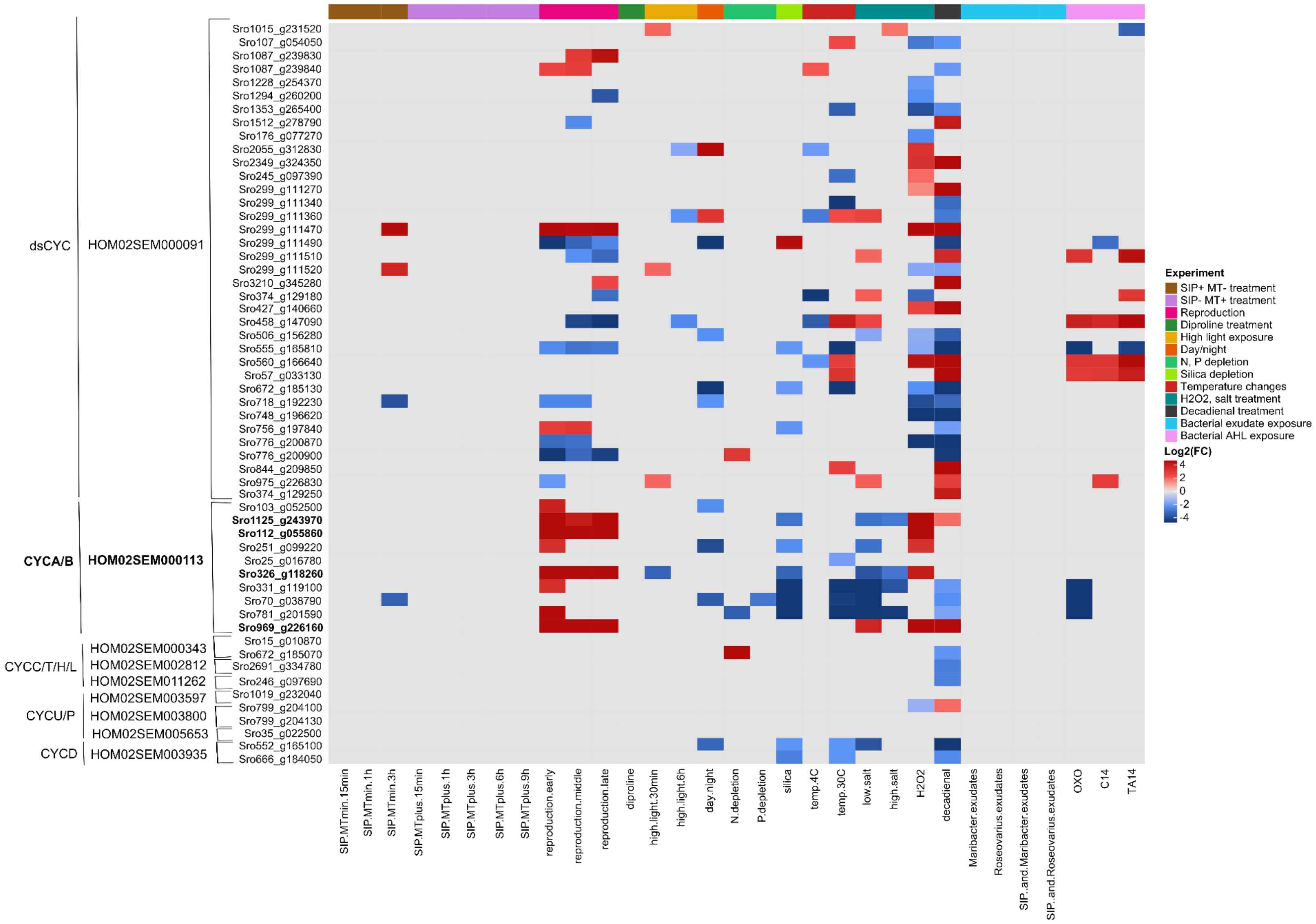

Supplementary Figure 19. Differential expression heatmap for *S. robusta* cyclins. CYCA/B family (*HOM02SEM000113*) is highlighted in bold, together with the cyclins from this family that potentially play a specific regulatory role during meiosis, since these are upregulated during the three reproduction stages but are not differentially expressed in day-night transition, which may suggests they are not involved in the traditional mitotic cell cycle.

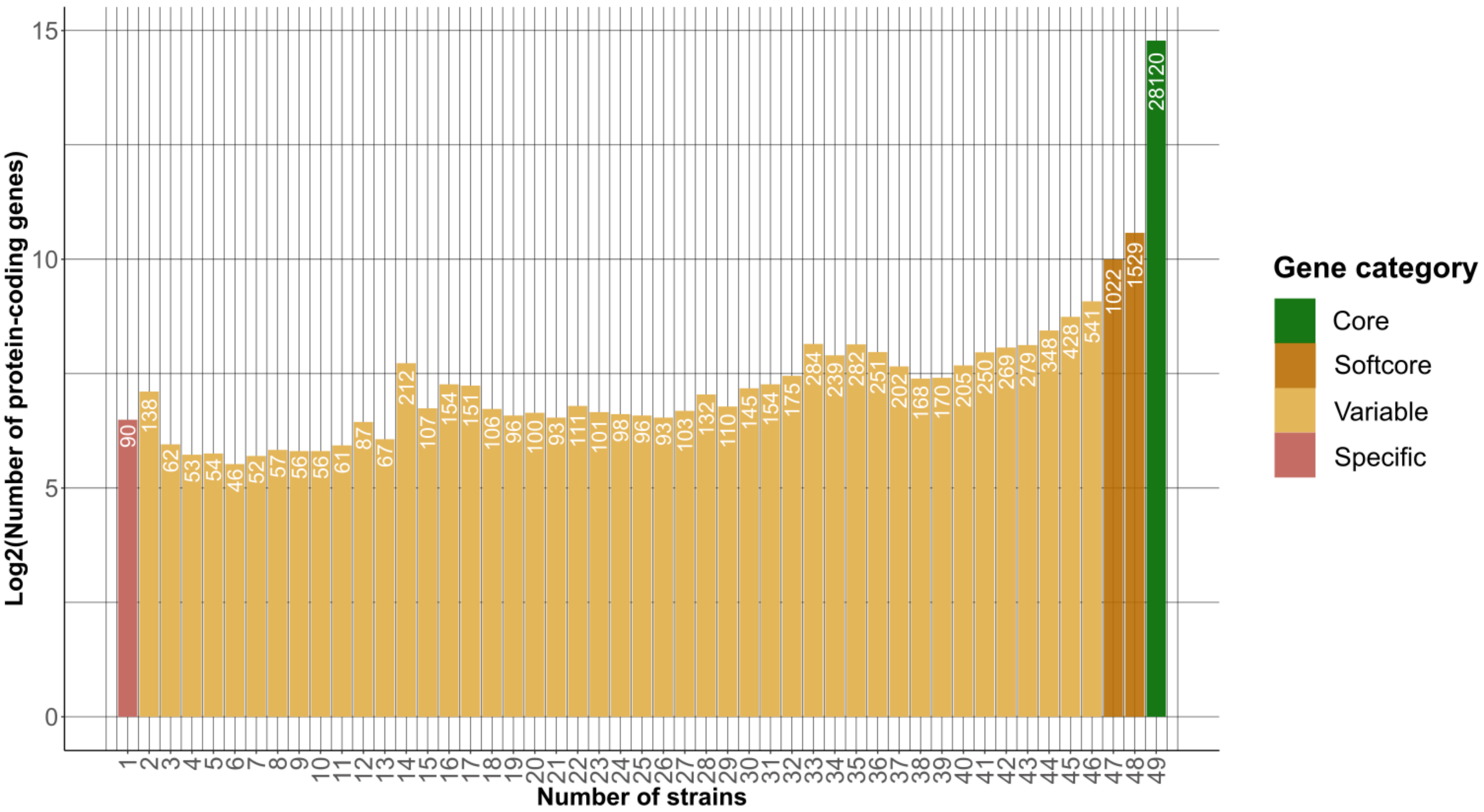

Supplementary Figure 20. Number of protein-coding genes found in a specific number of strains (1-49), colored by gene category. Core genes are genes present in all strains, softcore genes are genes present in >=95% of the strains, variable genes are genes present in <95% of the strains and specific genes are genes present only in one strain. Dispensable genes encompass these last three categories. White numbers refer to absolute number of genes. Source data are provided as a Source Data file.

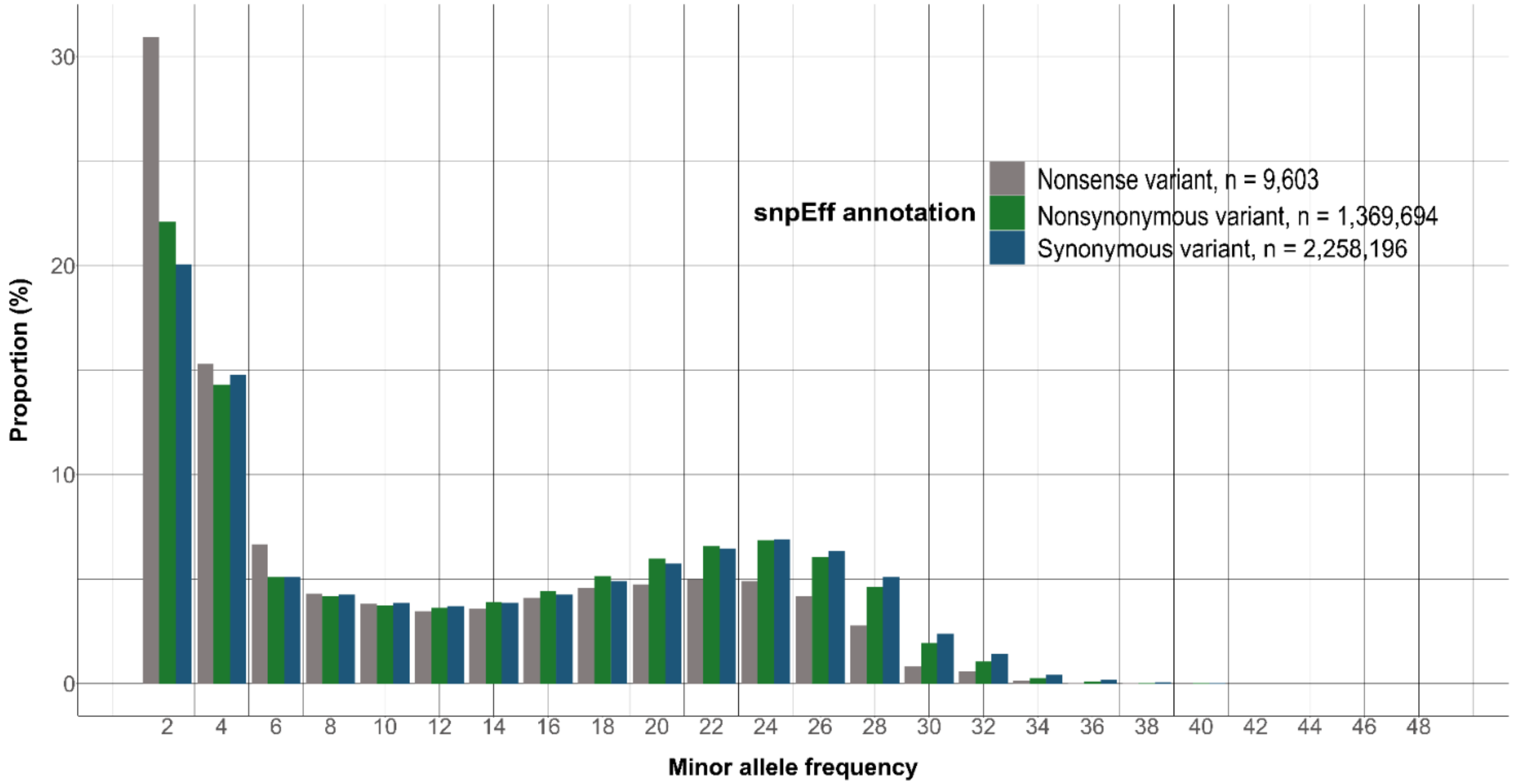

Supplementary Figure 21. Minor allele frequency proportions for synonymous, nonsynonymous and nonsense SNPs.

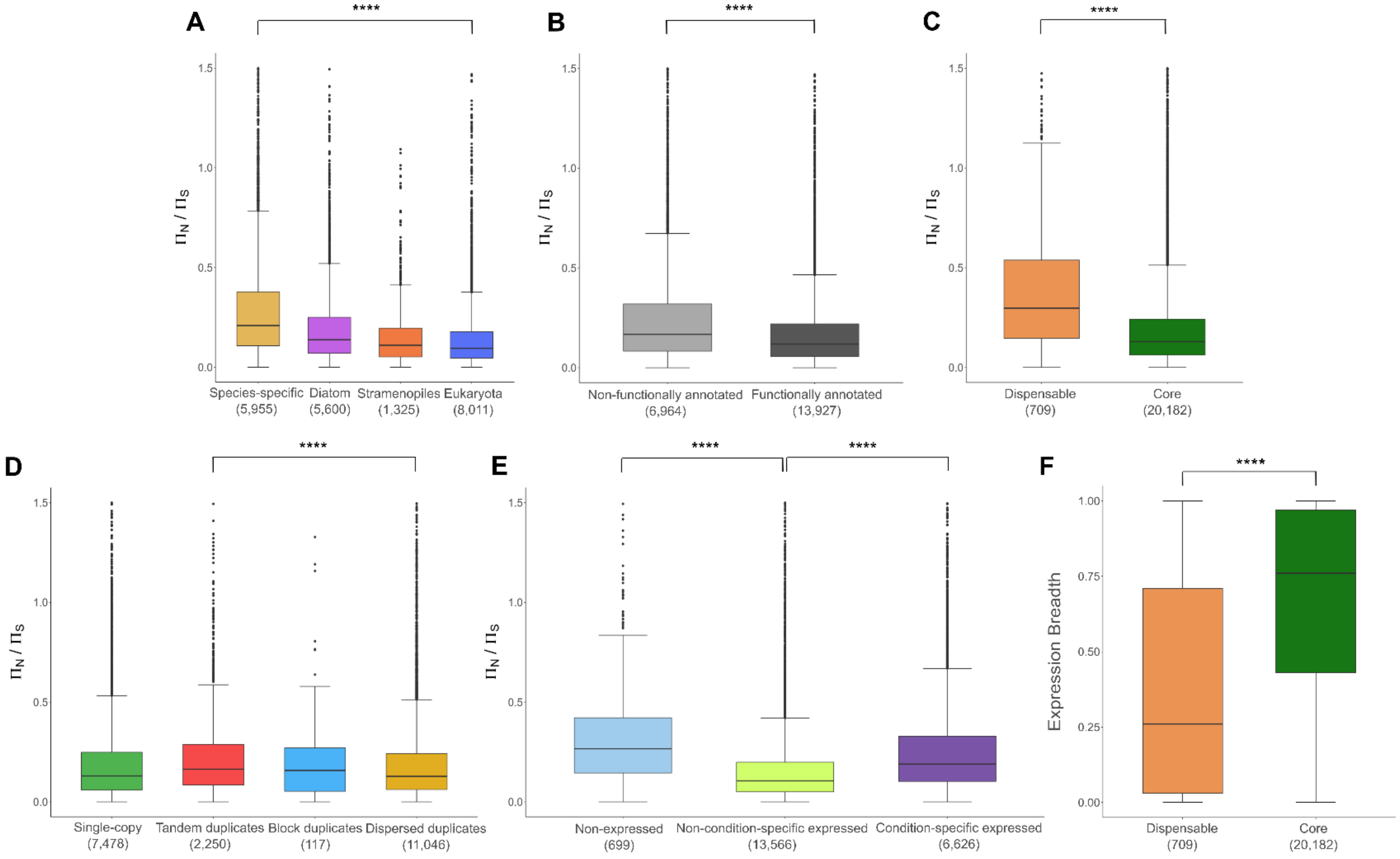

Supplementary Figure 22. Comparison of median π_N / π_S ratios between different gene groups. In A-F data are represented as boxplots. The line that divides the box into two parts indicates the median of the data. The lower end of the box shows the Q1 quartile while the upper end the Q3 quartile. The difference between Q1 and Q3 quartiles is called the interquartile range (IQR). The lower whisker extends from the hinge to Q1-1.5xIQR while the upper whisker extends from the hinge to Q3+1.5xIQR. Data beyond the whiskers are potential outliers and are plotted as individual dots. **(A)** Age category (species-specific π_N / π_S median = 0.223, eukaryota π_N / π_S median = 0.095). **(B)** Functional annotation category (non-functionally annotated π_N / π_S median = 0.175, functionally annotated π_N / π_S median = 0.119). **(C)** Pan gene category (dispensable π_N / π_S median = 0.335, core π_N / π_S median = 0.132). **(D)** Duplication category (single-copy π_N / π_S median = 0.134, all duplicates π_N / π_S median = 0.135, tandem duplicates π_N / π_S median = 0.166, dispersed duplicates π_N / π_S median = 0.130). **(E)** Expression category (non-expressed π_N / π_S median = 0.294, condition-specific expressed π_N / π_S median = 0.195, non-condition-specific expressed median = 0.107). Condition-specific expressed genes were defined as having a Tau >= 0.9 [17]. **(F)** Expression breadth of dispensable and core genes for which we have computed π_N / π_S ratios (dispensable expression breadth median = 0.26, core expression breadth median = 0.76). The total number of genes per group is reported in the x-axis by parenthesis. Significant differences in the median of π_N / π_S ratios between different gene groups discussed in the main text are highlighted by stars (pvalue<2.2e-16, Wilcoxon rank sum test, two-sided). Source data are provided as a Source Data file.

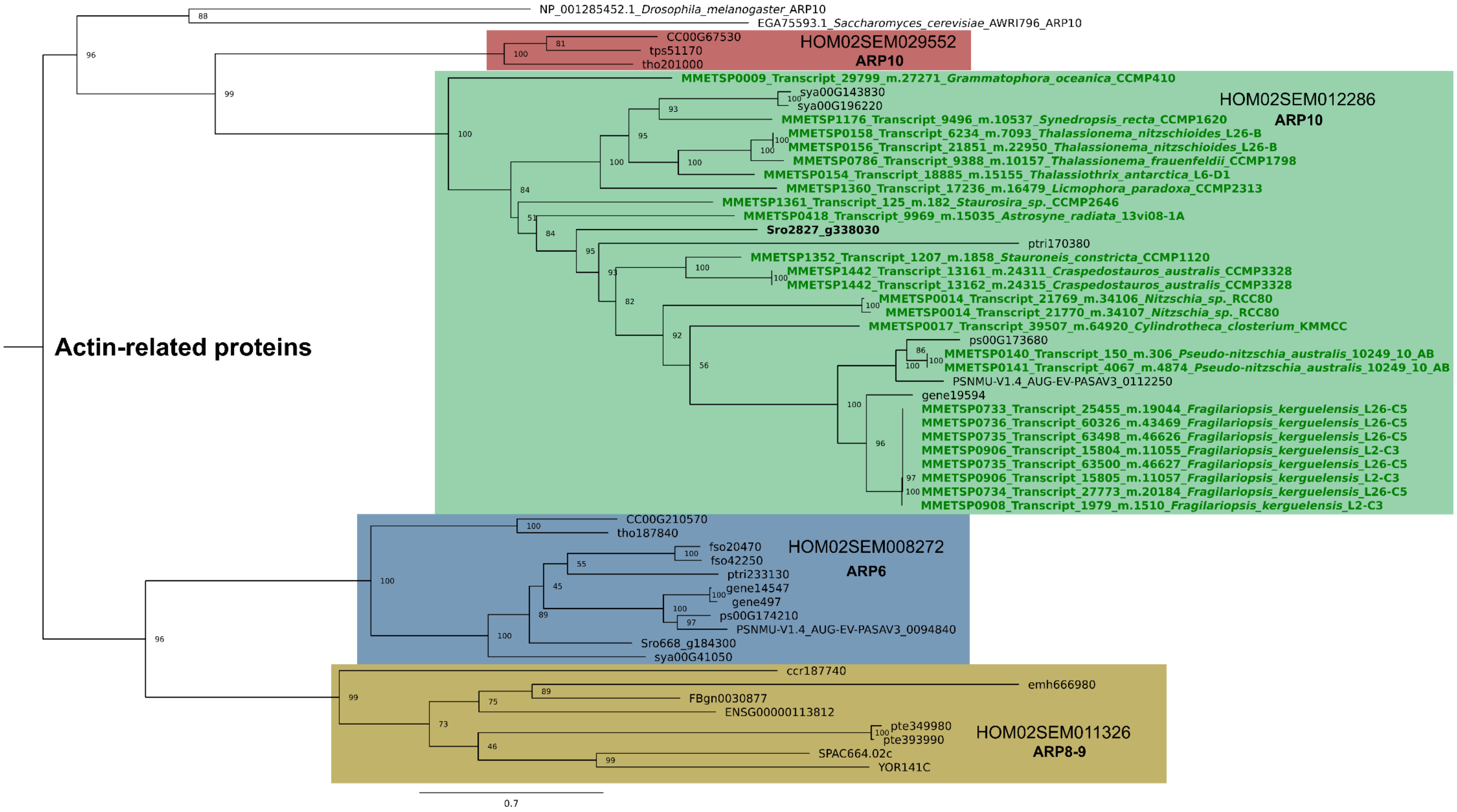

Supplementary Figure 23. Maximum likelihood phylogenetic tree of a selection of actin-related protein families and two external ARP10 sequences. Each gene family in PLAZA is highlighted in a different color. The *S. robusta* gene showing high pennate signature is highlighted in black bold while the corresponding MMETSP proteins from pennate diatoms that had hits with this gene are highlighted in green bold. Phylogenetic tree was computed using the following protocol: i) multiple sequence alignment of families of interest using MAFFT v7.187 [18], ii) automatic editing of this multiple sequence alignment using trimal v1.4.1 (–gt 0.1) [19], iii) phylogenetic tree construction using IQ-TREE v1.7 (-bb 1000 -mset JTT,LG,WAG,Blosum62,VT,Dayhoff -mfreq F -mrate R) [20] and iv) visualization and re-rooting using FigTree v1.4.4 [21].

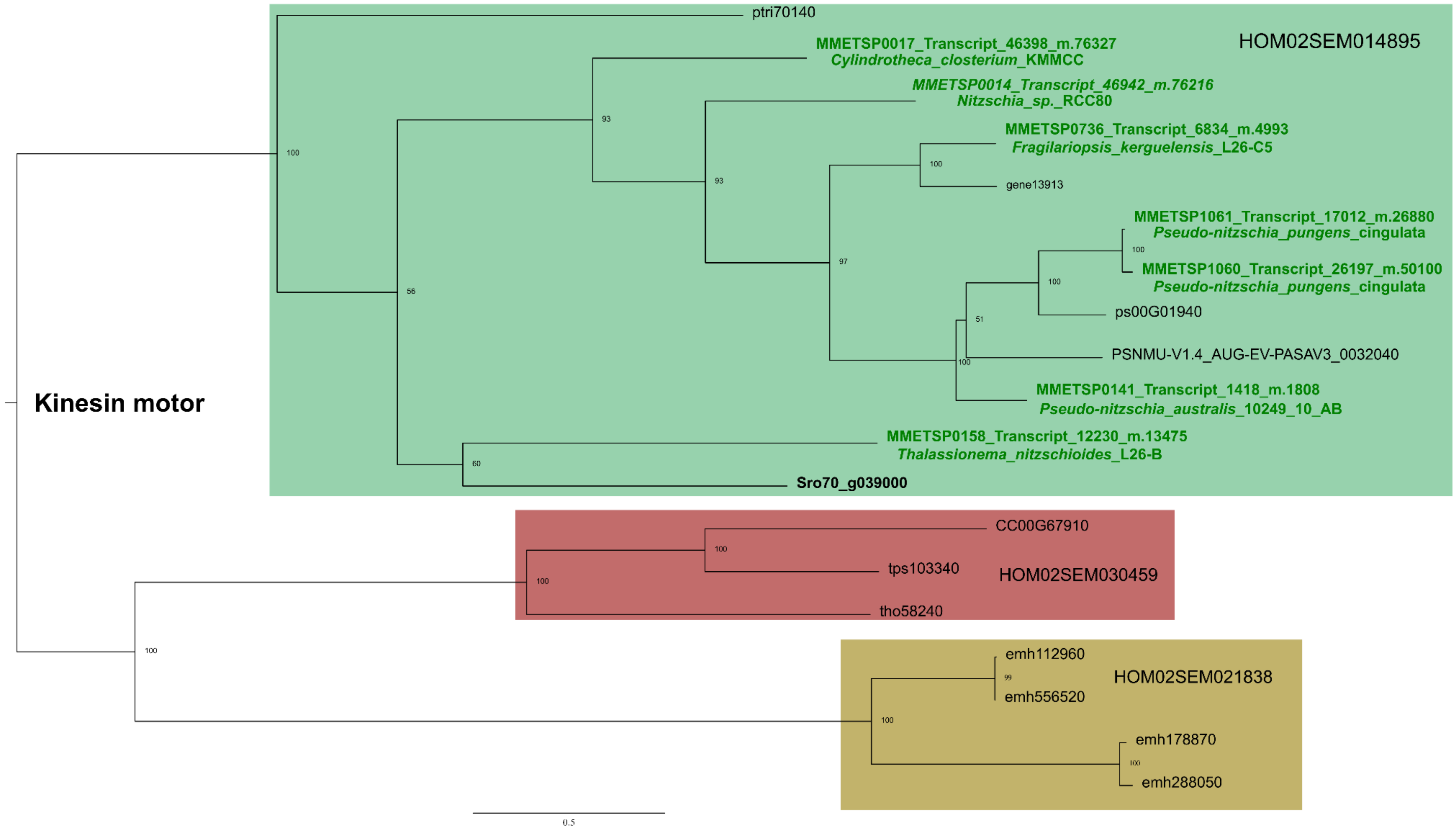

Supplementary Figure 24. Maximum likelihood phylogenetic tree of a selection of kinesin motor domain families. Each gene family in PLAZA is highlighted in a different color. The *S. robusta* gene showing high pennate signature is highlighted in black bold while the corresponding MMETSP proteins from pennate diatoms that had hits with this gene are highlighted in green bold. Phylogenetic tree was computed following the same protocol as Supplementary Figure 23.

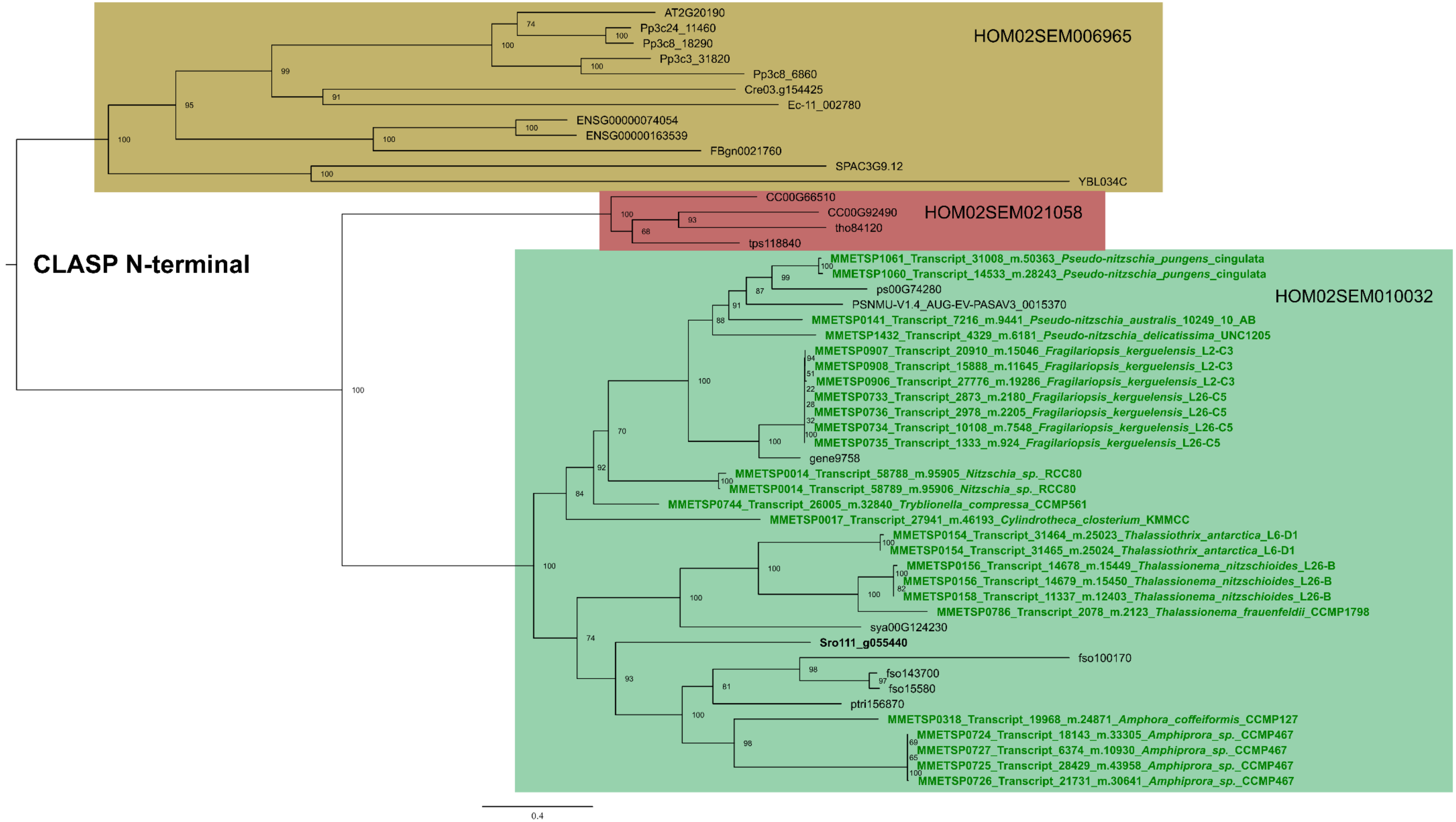

Supplementary Figure 25. Maximum likelihood phylogenetic tree of a selection of CLASP N-terminal families. Each gene family in PLAZA is highlighted in a different color. The *S. robusta* gene showing high pennate signature is highlighted in black bold while the corresponding MMETSP proteins from pennate diatoms that had hits with this gene are highlighted in green bold. Phylogenetic tree was computed following same protocol as Supplementary Figure 23.

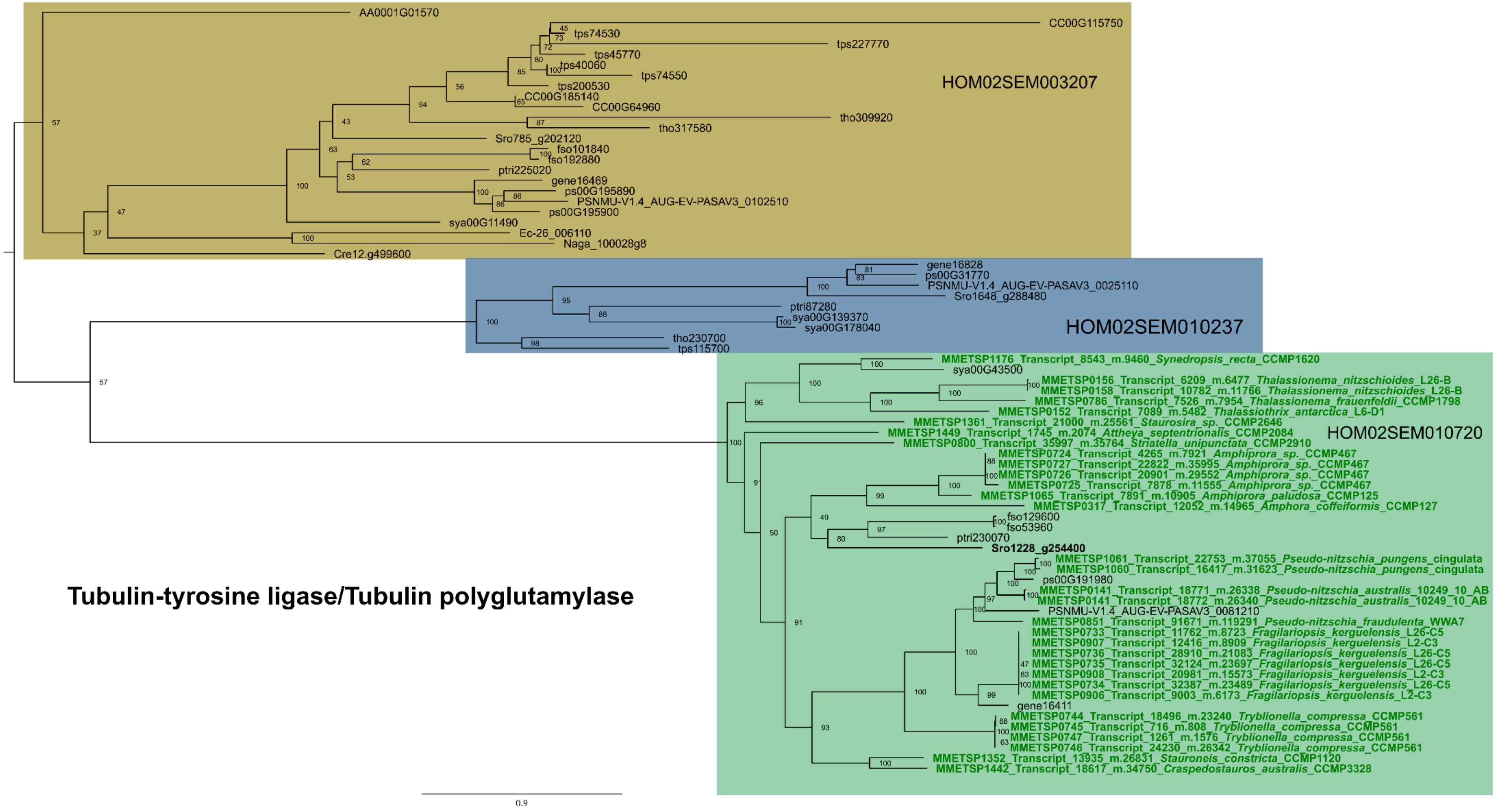

Supplementary Figure 26. Maximum likelihood phylogenetic tree of a selection of tubulin-tyrosine ligase/Tubulin polyglutamylase families. Each gene family in PLAZA is highlighted in a different color. The *S. robusta* gene showing high pennate signature is highlighted in black bold while the corresponding MMETSP proteins from pennate diatoms that had hits with this gene are highlighted in green bold. Phylogenetic tree was computed following same protocol as Supplementary Figure 23.

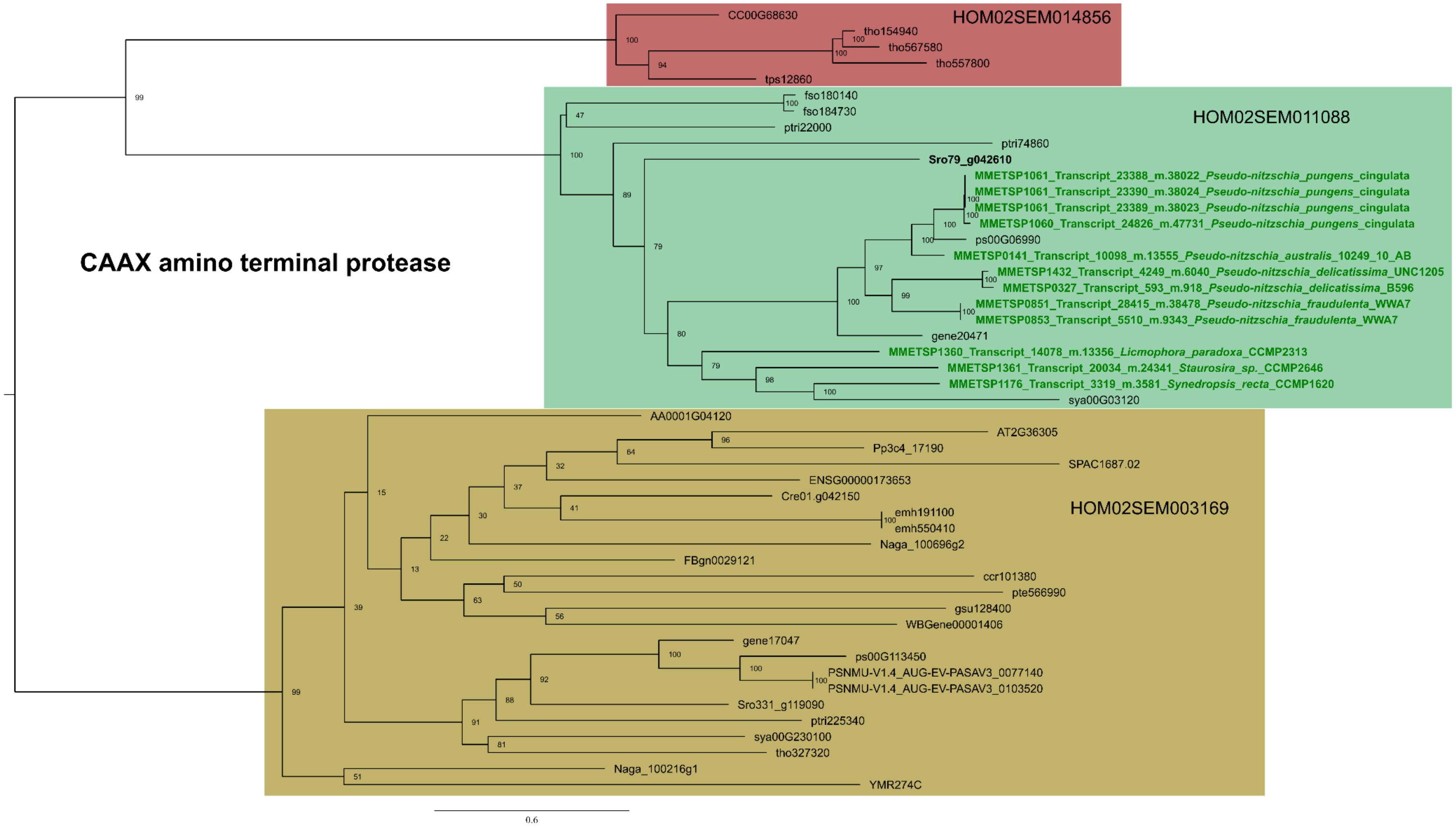

Supplementary Figure 27. Maximum likelihood phylogenetic tree of a selection of CAAX amino terminal protease families. Each gene family in PLAZA is highlighted in a different color. Each gene family is highlighted in a different color. The *S. robusta* gene showing high pennate signature is highlighted in black bold while the corresponding MMETSP proteins from pennate diatoms that had hits with this gene are highlighted in green bold. Phylogenetic tree was computed following same protocol as Supplementary Figure 23.

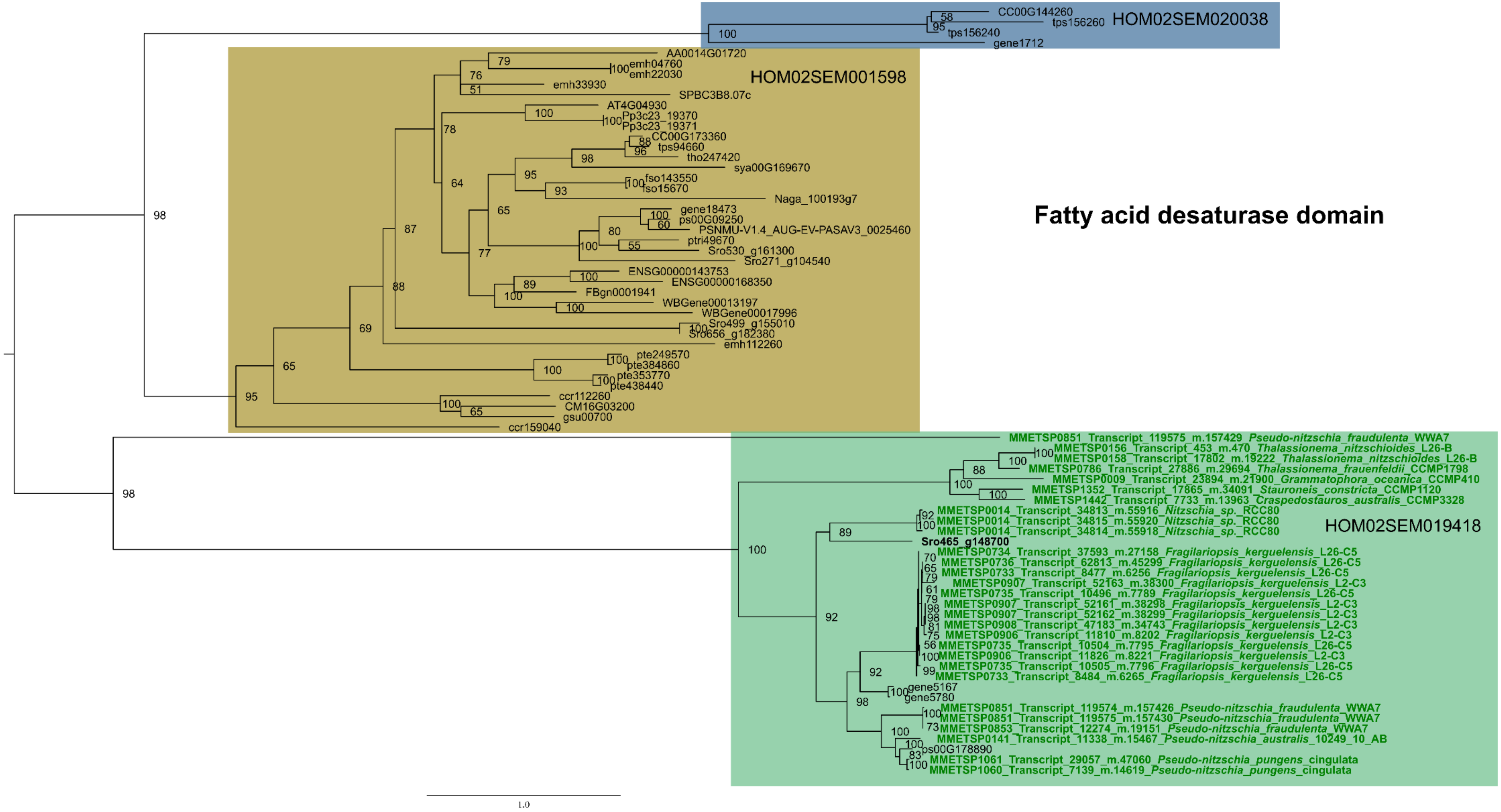

Supplementary Figure 28. Maximum likelihood phylogenetic tree of a selection of fatty acid desaturase families. Each gene family in PLAZA is highlighted in a different color. Each gene family is highlighted in a different color. The *S. robusta* gene showing high pennate signature is highlighted in black bold while the corresponding MMETSP proteins from pennate diatoms that had hits with this gene are highlighted in green bold. Phylogenetic tree was computed following same protocol as Supplementary Figure 23.

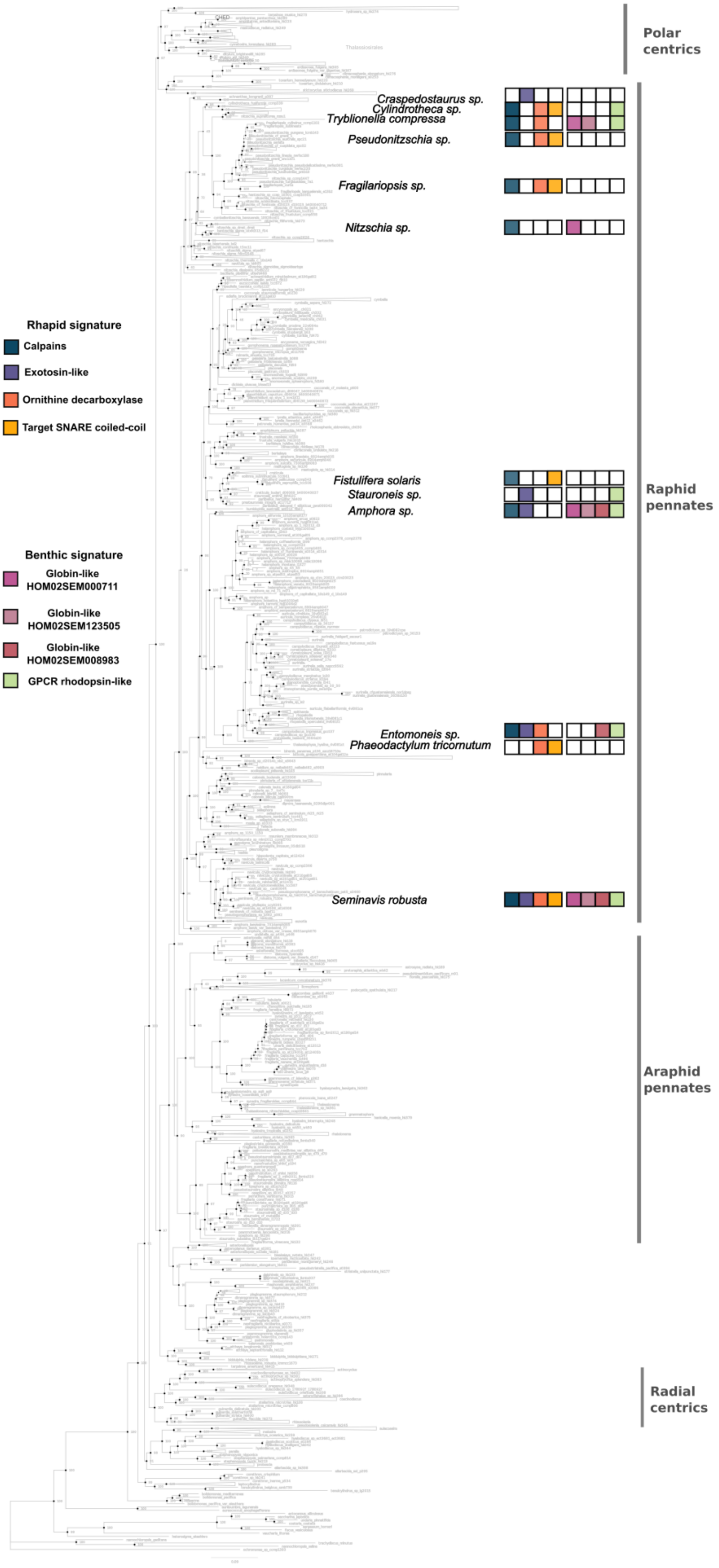

Supplementary Figure 29. Diatom species tree showing the occurrence of selected raphid-specific and benthic-specific traits described in this study. The diatom species tree is adapted from [22]. Globin-like gene families are based on Supplementary Figure 35. Source data are provided as a Source Data file.

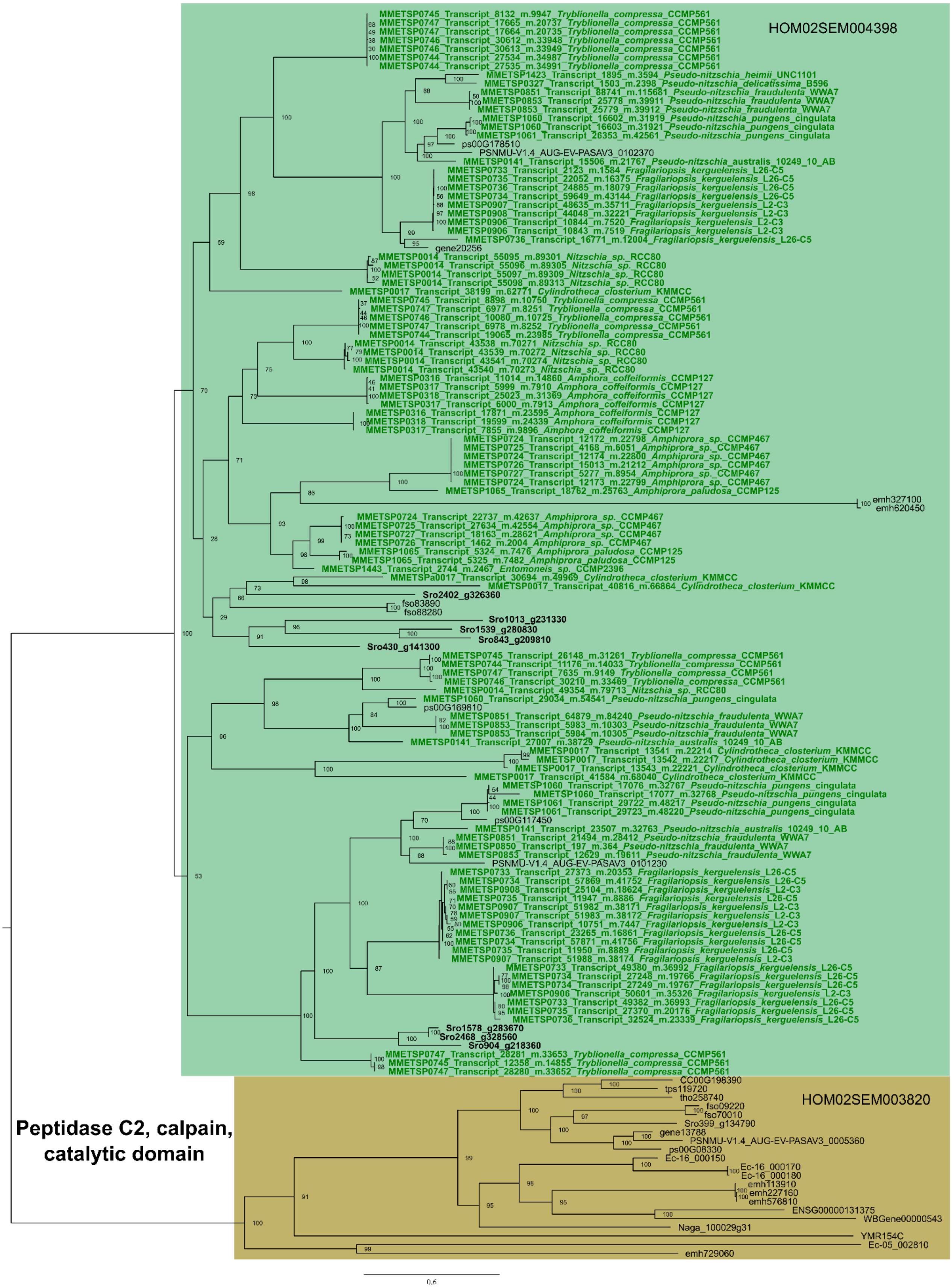

Supplementary Figure 30. Maximum likelihood phylogenetic tree of a selection of peptidase C2, calpain, catalytic domain families. Each gene family in PLAZA is highlighted in a different color. The *S. robusta* genes showing high raphid signature are highlighted in black bold while the corresponding MMETSP proteins from pennate raphid diatoms that had hits with these genes are highlighted in green bold. Phylogenetic tree was computed following same protocol as Supplementary Figure 23.

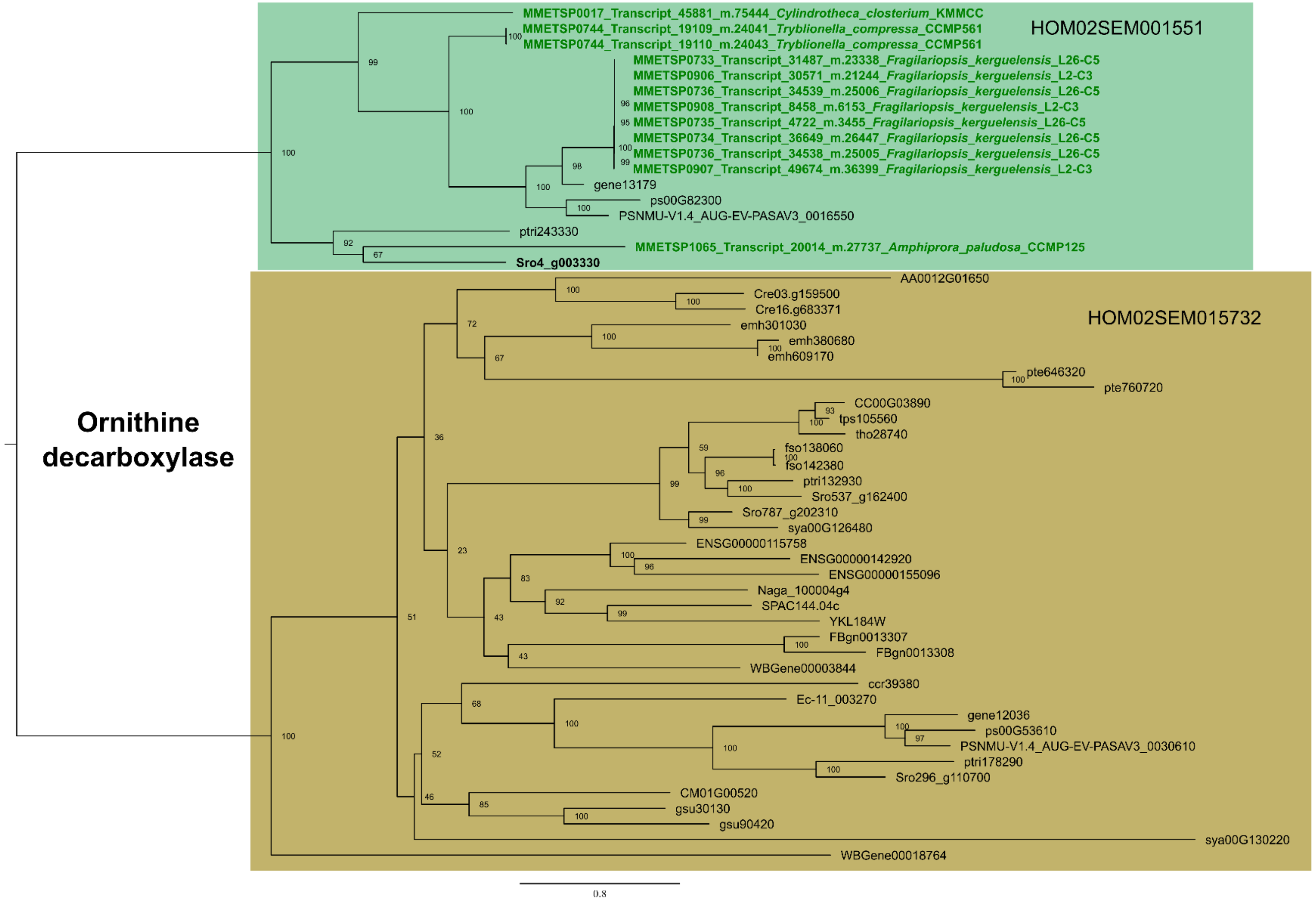

Supplementary Figure 31. Maximum likelihood phylogenetic tree of a selection of ornithine decarboxylase families. Each gene family in PLAZA is highlighted in a different color. The S. robusta genes showing high raphid signature are highlighted in black bold while the corresponding MMETSP proteins from pennate raphid diatoms that had hits with this gene are highlighted in green bold. Phylogenetic tree was computed following same protocol as Supplementary Figure 23.

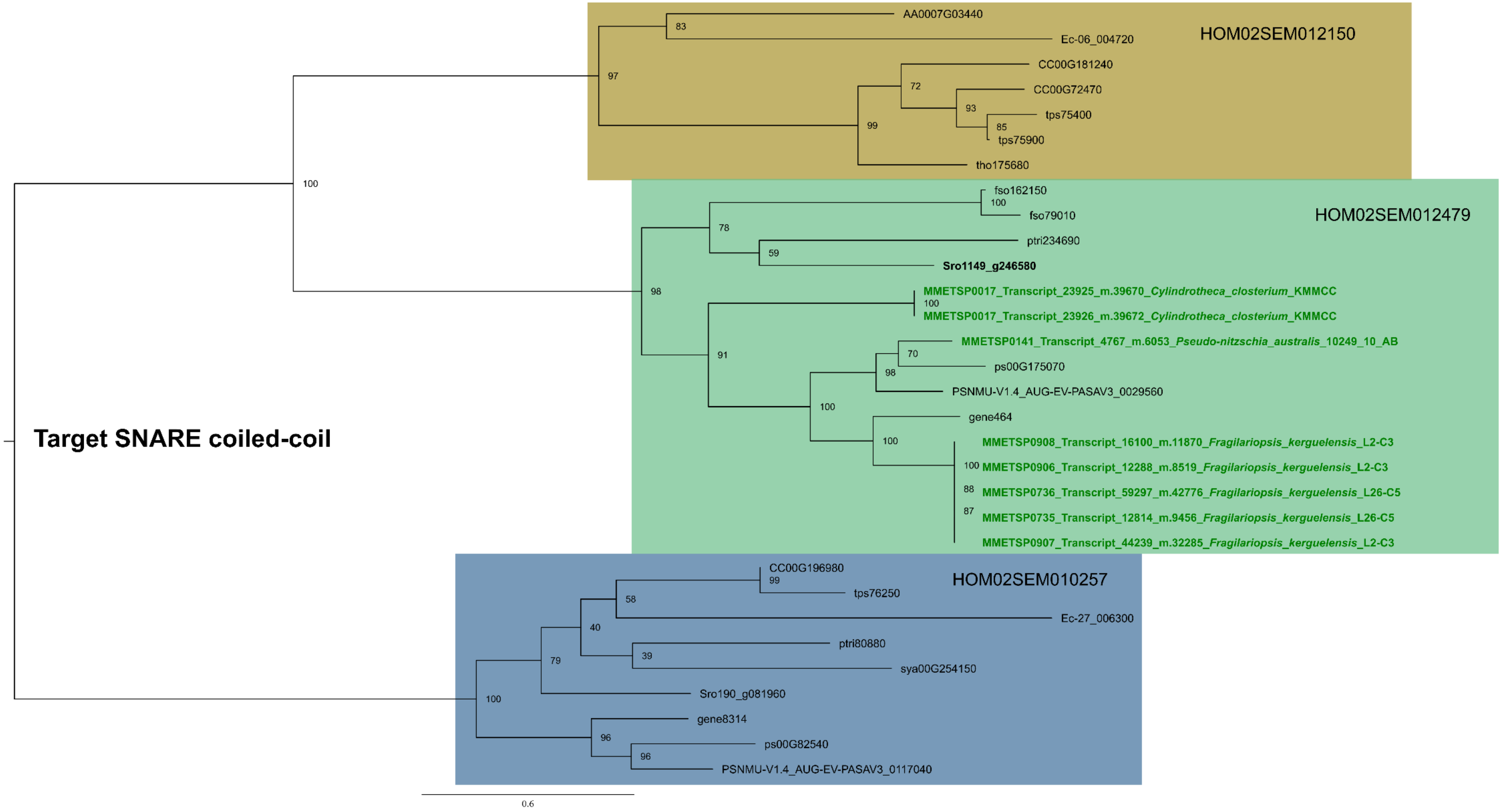

Supplementary Figure 32. Maximum likelihood phylogenetic tree of a selection of target SNARE coiled-coil homology domain families. Each gene family in PLAZA is highlighted in a different color. The *S. robusta* genes showing high raphid signature are highlighted in black bold while the corresponding MMETSP proteins from pennate raphid diatoms that had hits with this gene are highlighted in green bold. Phylogenetic tree was computed following same protocol as Supplementary Figure 23.

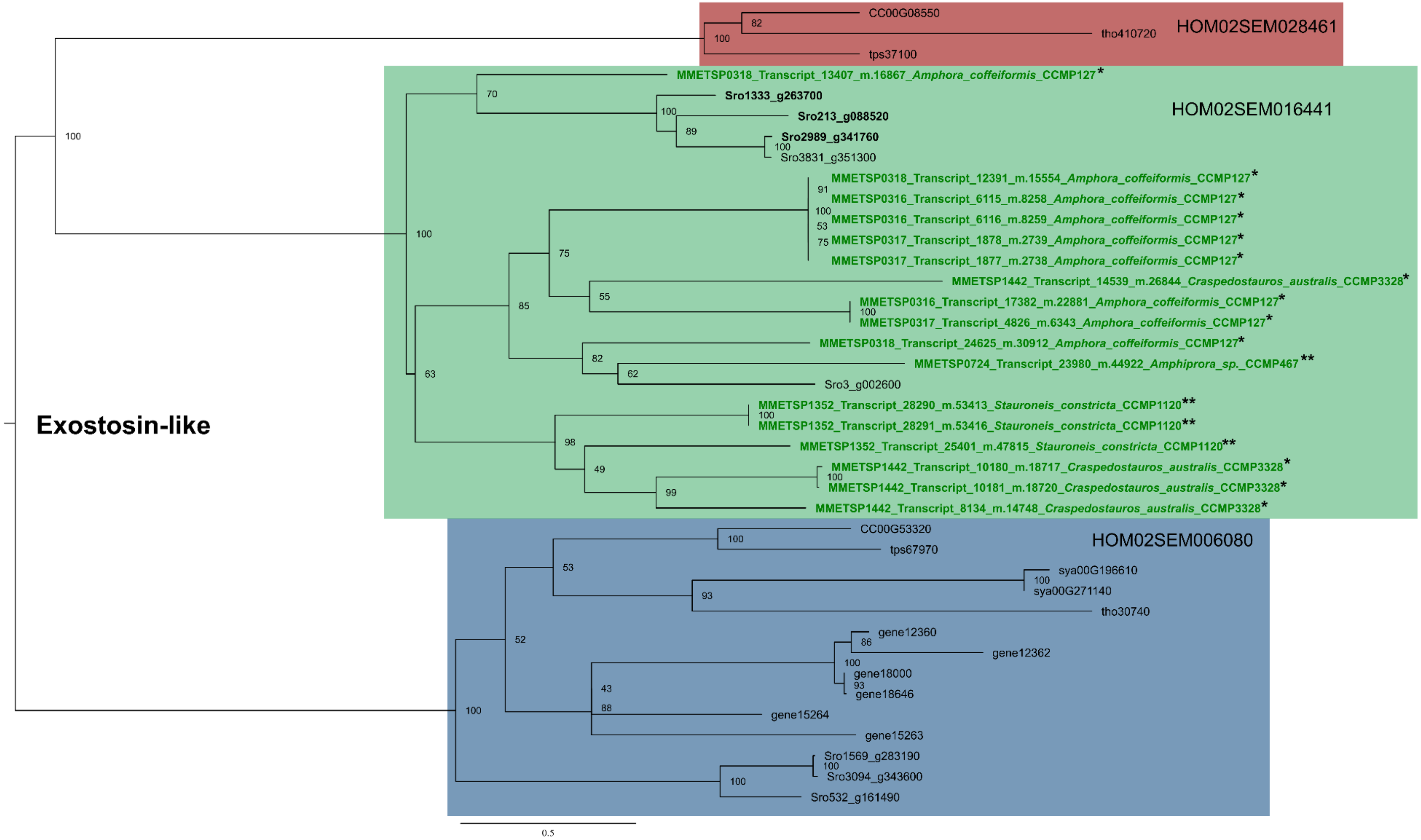

Supplementary Figure 33. Maximum likelihood phylogenetic tree of a selection of exostosin-like families. Each gene family in PLAZA is highlighted in a different color. The *S. robusta* genes showing both high raphid and benthic signature are highlighted in black bold while the corresponding MMETSP proteins from raphid species that had hits with these genes are highlighted in green bold. (*) denotes that the species strictly lives in the benthos and was used to compute the benthic signature, while (**) denotes the species can potentially live in the benthos and therefore was not used to compute the benthic signature. Phylogenetic tree was computed following same protocol as Supplementary Figure 23.

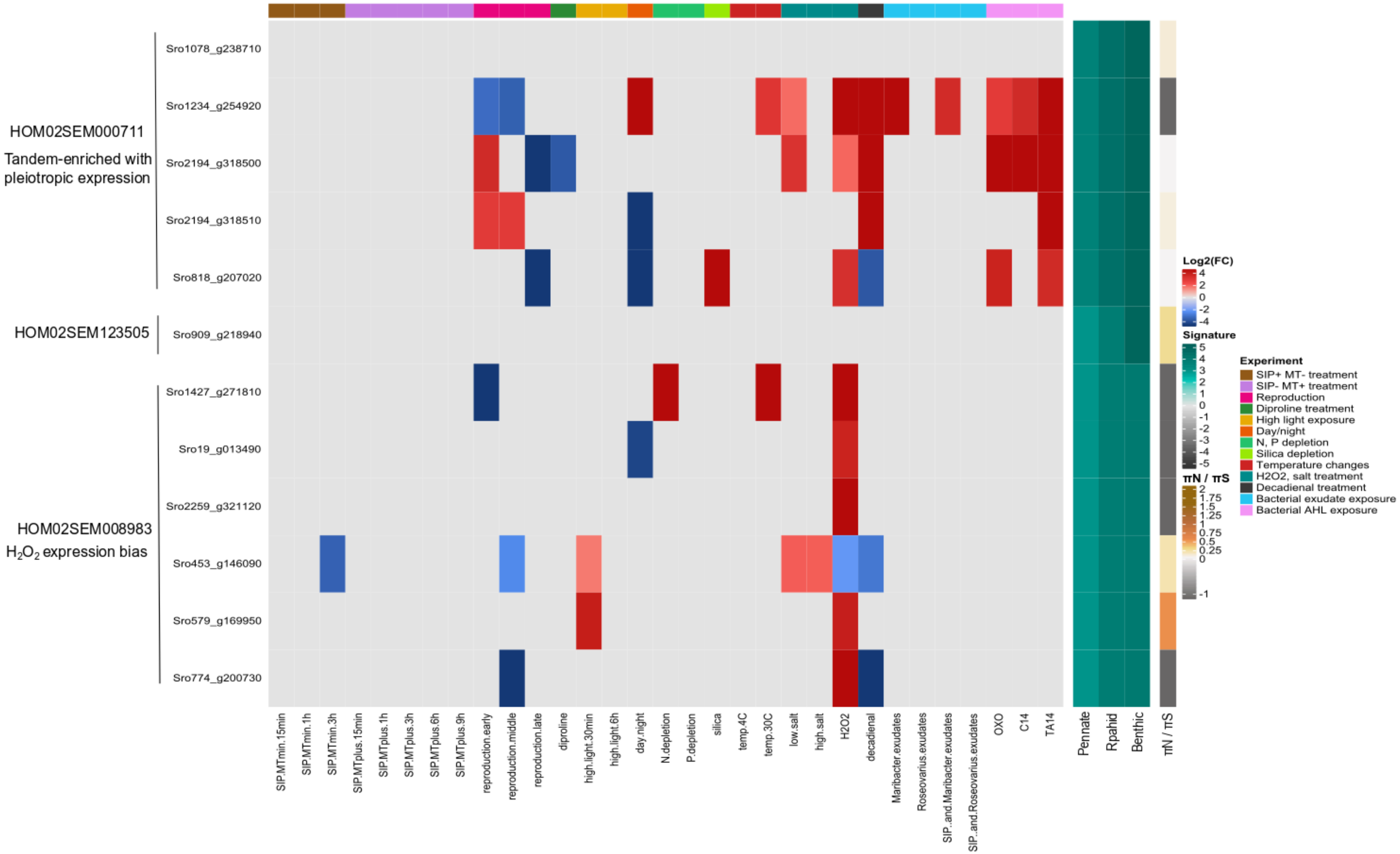

Supplementary Figure 34. Differential expression heatmap for globin-like proteins with high benthic signature. π_N / π_S values of -1 indicate no data is available for this gene. Globins from the family HOM02SEM008983 were found only in two species, *Amphora coffeiformis* (strictly benthic) and *Amphiprora sp. CCMP467* (potentially benthic). As such, these globins were considered to show high benthic signature.

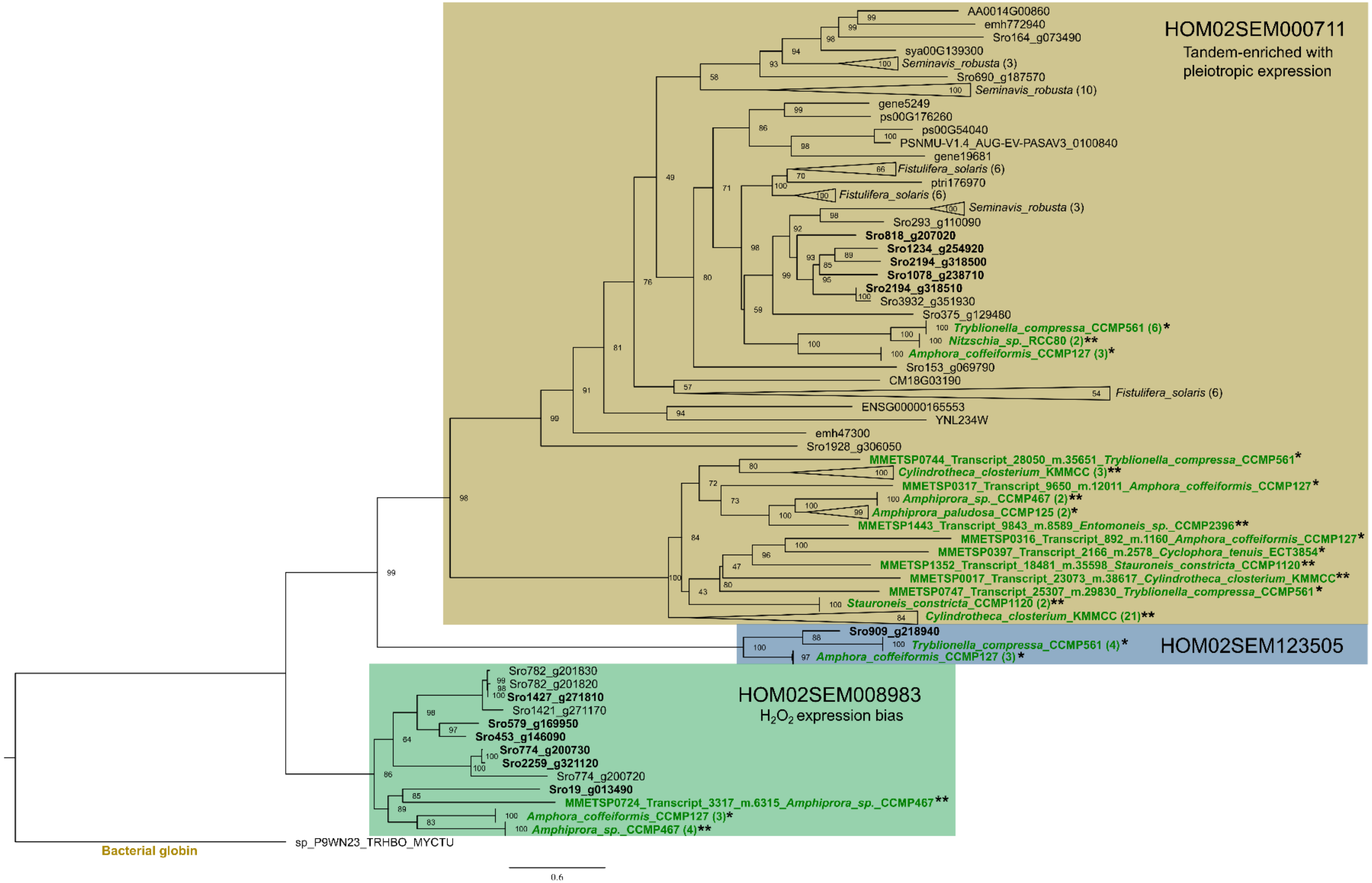

Supplementary Figure 35. Maximum likelihood phylogenetic tree of the globin-like *S. robusta* genes showing high benthic signature. Each gene family in PLAZA is highlighted in a different color. The *S. robusta* genes showing high benthic signature are highlighted in black bold while the corresponding MMETSP proteins that had hits with these genes are highlighted in green bold. (*) Denotes that the species strictly lives in the benthos and was used to compute the benthic signature, (**) denotes the species can potentially live in the benthos or is an ice benthic species, these were not used to compute the benthic signature. Globins from the family HOM02SEM008983 were found only in two species, *Amphora coffeiformis* (strictly benthic) and *Amphiprora sp. CCMP467* (potentially benthic), these globins were still considered to show high benthic signature. HOM02SEM008983 and HOM02SEM123505 represent potential benthic-specific gene families. Numbers in parenthesis refer to the number of proteins in that branch of the phylogenetic tree. Phylogenetic tree was computed following the same protocol as Supplementary Figure 23.

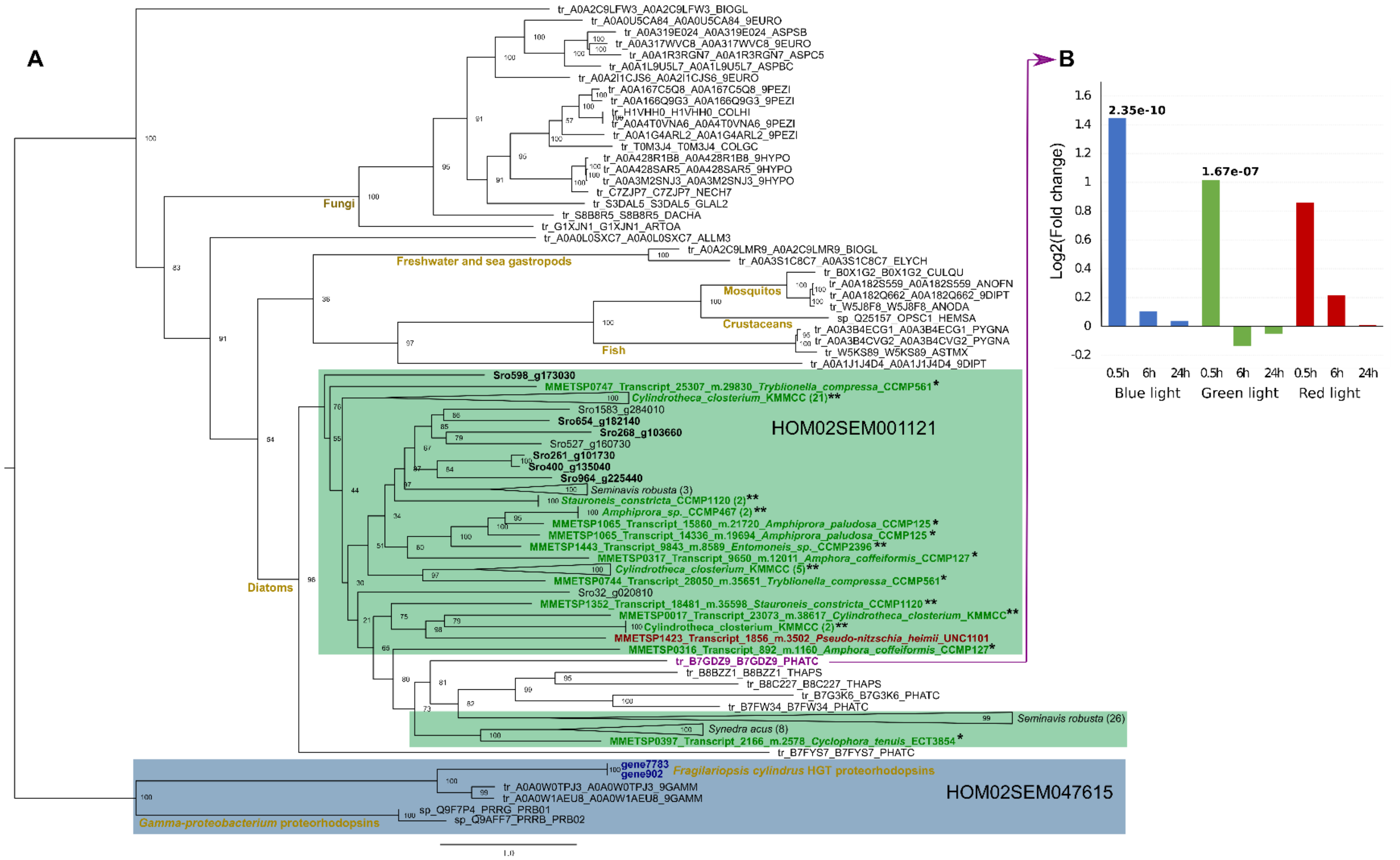

Supplementary Figure 36. Analysis of the GPCR rhodopsin-like *S. robusta* genes from HOM2SEM001121 family showing high benthic signature. (A) Maximum likelihood phylogenetic tree including proteorhodopsins from bacteria and *Fragilariopsis cylindrus* (blue bold), as well as best hits from a blastp search of *Sro964_g225440* (chosen as a representative) against the UniProt database [23] (E-value < 0.1). Each gene family in PLAZA is highlighted in a different color. The *S. robusta* genes showing high benthic signature are highlighted in black bold while the corresponding MMETSP proteins that had hits with these genes are highlighted in green bold (benthic species) or red bold (planktonic species). (*) Denotes that the species strictly lives in the benthos and was used to compute the benthic signature, (**) denotes the species can potentially live in the benthos or is an ice benthic species, these were not used to compute the benthic signature. Numbers in parenthesis refer to the number of proteins in that branch of the phylogenetic tree. Phylogenetic tree was computed following the same protocol as Supplementary Figure 23. **(B)** *Phaeodactylum tricornutum* closest ortholog expression under different light conditions [24]. Differential expression was computed using limma v3.38 [25], significant p-values are shown in bold.

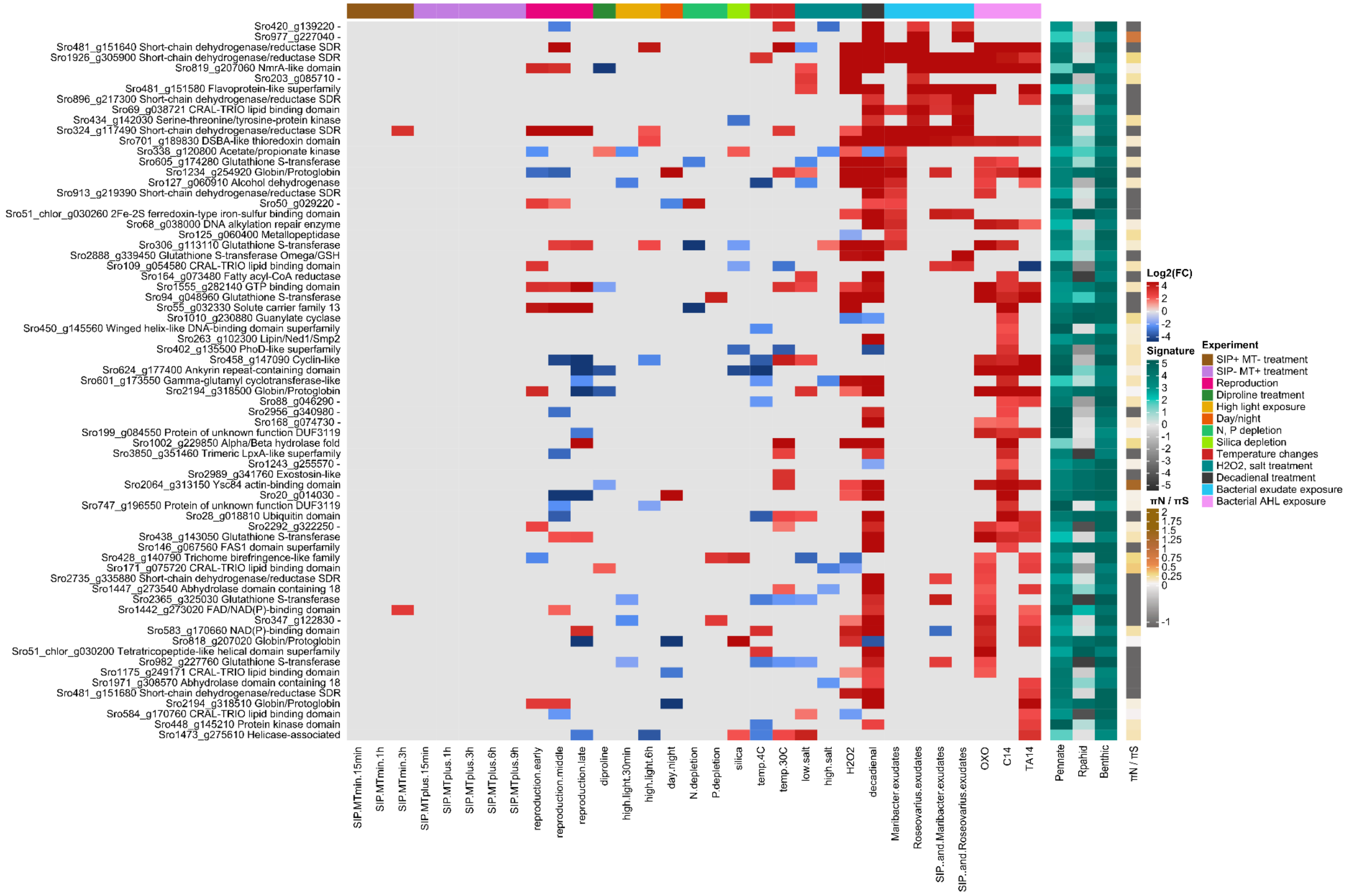

Supplementary Figure 37. Differential expression heatmap for genes with high benthic signature and differentially expressed in bacterial interaction experiments. π_N / π_S values of -1 indicate no data is available for this gene.

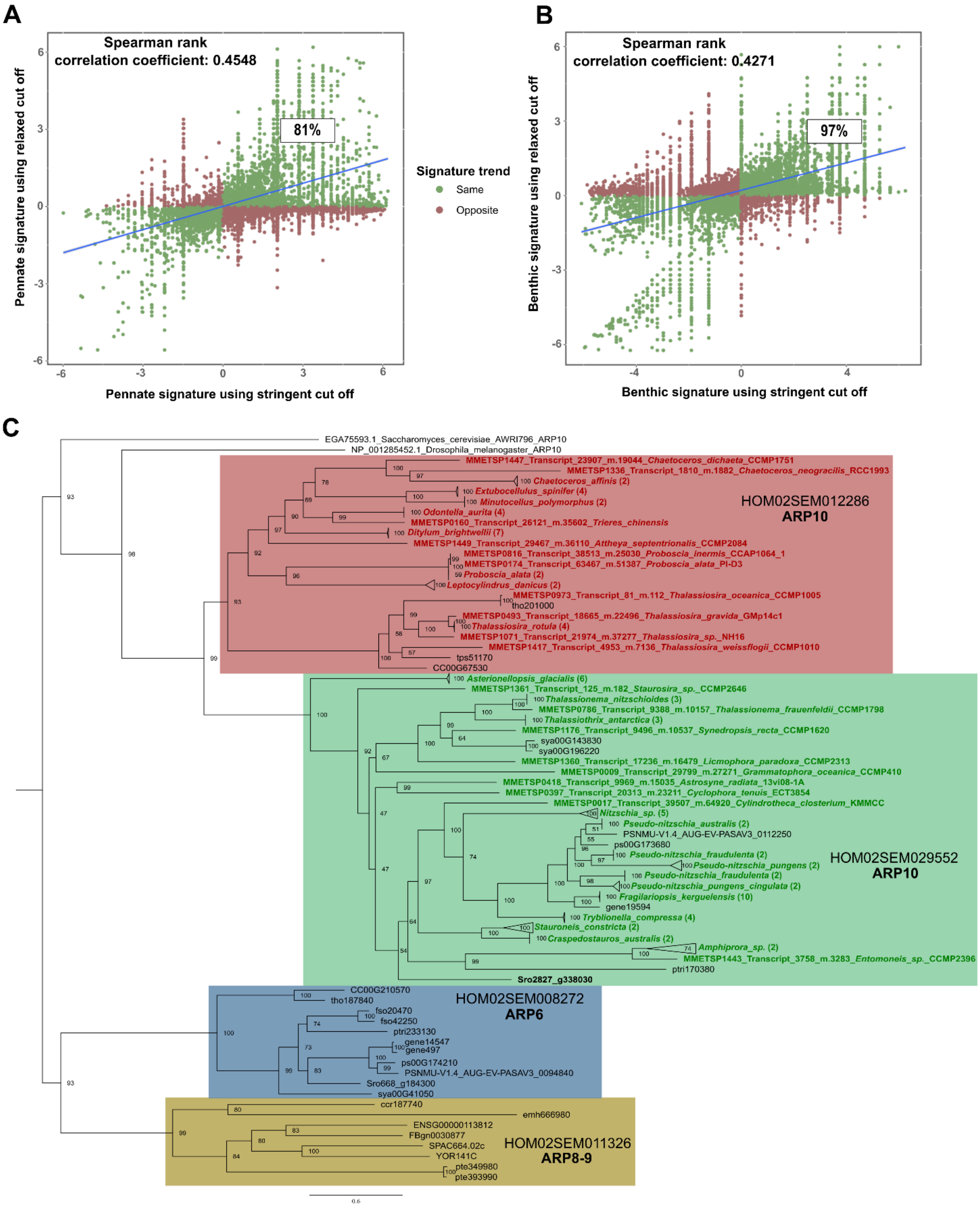

Supplementary Figure 38. Control experiments for signature gene analysis. Pennate **(A)** and benthic **(B)** signature gene correlation plot where the x-axis shows the signature values using a stringent cut off (--evalue 1e-05 --min-score 200) while the y-axis shows the signature values using a relaxed cut off (--evalue 1e-03 --min-score 0). The displayed percentage refers to the number of genes with high signature that show the same positive trend using both cutoffs. **(C)** Phylogenetic tree of actin-related families containing a gene with high pennate signature (Sro2827_g338030, in bold) using the relaxed cutoff, which recovers MMTESP proteins from additional pennate species (green bold) as well as more distant centric species (red bold). The addition of distantly related centric homologs, clustering in HOM02SEM012286, does not interfere with the pennate signature observed in the sub-tree containing Sro2827_g338030 (HOM02SEM029552). The tree generated using the stringent cutoff is shown in Supplementary Figure 23. Branches with multiple proteins from the same species are collapsed (showing in parenthesis the number of collapsed proteins).

Supplementary Table 1. Summary of the *S. robusta* genome DNA sequencing data and pre-processing steps.

Read type	No. of reads	Total size (Gb)	Average read length (bp)	Base coverage
Illumina paired-end[1]	18,761,184 x 2	24	296	79x
PacBio 11 SMRT cells[2]	1,199,840	6.1	5,203	34x

[1] BBDuk tool v34.56 [26] was used to clean the Illumina paired-end reads using k-mers with the settings k=27, ktrim=r, mink=10, minlength=80, qtrim=rl, trimq=15 and hdist=1. The remaining paired-end reads were merged using PEAR v0.9.6 [27] with the parameters p=0.01, g=2 and v=30.
[2] PacBio sequencing long reads were corrected with the Illumina short reads using LoRDEC version 0.5 [28] with the parameters k=19 and s=2.

Supplementary Table 2. Contiguity and quality metrics for different *S. robusta* genome assemblies.

Metric	Illum1[1]	PacB2[2]	Hybrid1[3]	Hybrid2[4]	Final genome assembly[5]
Number of scaffolds	17,890	4,068	1,665	6,218	4,754
Longest scaffold (bp)	181,666	426,596	538,151	295,434	318,047
Smallest scaffold (bp)	1,000	2	3,690	992	950
N50 (bp)	14,837	63,875	79,695	40,890	50,720
N90 (bp)	2,551	12,257	28,319	9,859	13,123
L90	10,029	2,082	1,168	3,261	2,608
NG50 (bp)	10,361	38,870	41,015	32,035	39,084
Assembly size (bp)	123,807,665	112,751,129	96,170,342	126,503,855	125,760,934
Computationally/experimentally estimated genome size (Mb)	117-151	117-151	117-151	117-151	117-151
GC content (%)	48.38	48.31	48.25	48.34	48.32
Illumina reads mapping (%)	88.76	65.51	73.74	91.17	91.10
Illumina read pairs mapping concordantly (%)	83.12	62.87	64.98	85.33	85.13
SANGER sequences found (Identity>=85) (%)	100	93.86	98.25	100	100
SANGER sequences found (Identity>=85 & Coverage>=95) (%)	84.21	43.86	64.91	84.21	84.21
Completeness, Bacillariophyta mapped transcripts (Identity>=85) (%)	99.44	83.62	68.82	99.70	99.68
Completeness, Bacillariophyta mapped transcripts (Identity>=85 & Coverage>=95) (%)	88.03	75.88	59.13	95.05	95.25

[1] **Illum1:** Platanus v1.2.4 tool [29] was executed with k-mer autoextension 10 and the parameters u=0.1, k=52 and a=5.0. The resulting contigs were scaffolded, gaps were filled, and the sequences were filtered by a minimum length of 1,000 pb.

[2] **PacB2:** Circular Consensus Sequencing (CCS) reads were generated from PacBio raw reads using SMRTAnalysis v2.2.0 [30] with following parameters: minFullPasses 0, minPredictedAccuracy 80%, minLength 90 bp and maxLength No Limit. Next, canu v1.4 [31] was used for self-correcting (-correct and genomeSize=136.0m errorRate=0.035 -pacbio-raw and stopOnReadQuality=false) and trimming (–trim genomeSize=136.0m errorRate=0.035 -pacbio-corrected) of these CCS reads. Lastly, FALCON-integrate v5 [32] was used to build the assembly using the following parameters: input_type = preads, length_cutoff = 50, length_cutoff_pr = 500, pa_HPCdaligner_option = -v -B128 -t16 -e0.85 -M24 -l500 -k18 -h60 -w8 -s500, ovlp_HPCdaligner_option = -v -B128 -t32 -M24 -k24 -h60 -e.90 -l1000 -s500, pa_DBsplit_option = -a -x500 -s500, ovlp_DBsplit_option = -x500 -s500, falcon_sense_option = --min_idt 0.70 --min_cov 1 --max_n_read 500 --n_core 8, falcon_sense_skip_contained = False and overlap_filtering_setting = --max_diff 400 --max_cov 400 --min_cov 1 --n_core 12.

[3] **Hybrid1:** Corrected PacBio reads were integrated in Illum1 assembly via DBG2OLC v2015-05-19 [33] program with the parameters K=23, AdaptiveTh=0.01, KmerCovTh=15, MinOverlap=75 and RemoveChimera=1. Then, heterogeneity cleaning process (see main text) was applied to remove redundant sequences.

[4] **Hybrid2:** The heterogeneity cleaning process was applied on the Illum1 assembly prior the PacBio data integration to have an Illumina assembly with less redundancy and complexity. Then, PacBio reads were integrated with PBJelly v15.8.24 [34], which uses BLASR to map the PacBio reads with the parameters minMatch=8, minPctIdentity=70, bestn=8, nCandidates=30, maxScore=500, sdpTupleSize=8 and noSplitSubreads.

[5] **Final genome assembly:** Protocol for the final genome assembly and computation of quality metrics are described in the main text and Supplementary Note 2. Platanus and PBJelly were executed with the same parameters mentioned in Illum1 and Hybrid2.

Supplementary Table 3. Product size of the *S. robusta* genome regions amplified by PCR.

Scaffold	Positions	Size (bp)	Primers	Tm	Product size (bp)
Sro_contig122	13120-14314	1,194	TGAAGAAAAGCACAAAGATCCA<br>GGTACGGTGCAGGAGGGATA	59.86<br>62.19	1,190
Sro_contig1206	7669-9246	1,577	ATTACATTTTAGCACCAAGTGGTAACG<br>GACAGCAACACGGAGGAAGA	62.53<br>61.4	1,575
Sro_contig178	21778-23221	1,443	GTTCCTGTCTTCCCCCGTTT<br>AAGCGGAACACGAAAATGAC	62.51<br>60.12	1,443
Sro_contig871	29747-30757	1,010	ACTGGCCGCGATCGTTAC<br>GCTATCTGGAAGCGTGTCAA	62.19<br>59.03	996
Sro_contig182	87022-88355	1,333	GGCAGCTGCACAAAAGGAG<br>ATTCCACAACACTCTTTTCCGTCT	62.07<br>62.4	1,326
Sro_contig764	22859-24429	1,570	CGGGCGGAATGGTAAGTG<br>GGTATTGTCTCATTTGTATTGGAGCTT	62.4<br>61.94	1,544
Sro_contig1294	21927-23133	1,206	CGGCCTTCAGTCGAGTCTT<br>TGGAGCGTTTGGTATTGATG	60.54<br>59.54	1,200
Sro_contig369	39874-40922	1,048	TACGACTTCCAAAATACGGCAAC<br>GTGCACAAGTGCCCGACTAT	62.34<br>61.13	1,032
Sro_contig456	32403-33407	1,004	tagacaaaaggaaggatcgtaaa<br>CAGCTACCTGGCGCAGTG	57.19<br>61.78	1,004
Sro_contig119	91247-92684	1,437	GTGCTGGTGATTCGATTCCT<br>AGTAAGTTGATTGACTCCAGCTTTT	60.08<br>58.99	1,435
Sro_contig1594	3590-4926	1,336	TAACTCGATCATCCAGAGTTTATCT<br>TGCGAAGCCCACTTAGGAG	57.62<br>61.46	1,336
Sro_contig937	29844-31139	1,295	CTTGTCATTGTGATTGACTCAGC<br>CACCTCAGATCTGCGACGA	59.79<br>61.17	1,283
Sro_contig157	26932-28015	1,083	ATGGTTTGGCCGATCGAA<br>CTCGGGTACCTCCCGTTG	62.38<br>61.47	1,077
Sro_contig276	68823-70352	1,529	TCCACTTTGAAAAGAACAATCC<br>ATCACGAAAGCTAGAAGGGAGGT	58.21<br>61.77	1,529
Sro_contig191	2470-3549	1,079	TCGTTTTGTACAGACGCATGA<br>GGATATTACTAGCTGTAGTCCCAGTTT	60.31<br>58.78	1,076
Sro_contig1481	14825-15929	1,104	GACTCTCAAATACGACGATTTCAAC<br>CGCGTCCAATAGCAATCAT	60.4<br>59.66	1,104
Sro_contig1351	19106-20244	1,138	AGGTCCACCAGAACCGAAAC<br>TTTTTAGGGCTGCTCAGTTCA	61.32<br>60	1,120
Sro_contig1247	14571-16075	1,504	CCAAACTGTATCCCTTGCTC<br>ATCATCCTCTCTTAGCTTTCAGC	57.27<br>58.33	1,504
Sro_contig461	50342-51414	1,072	TTGAGACGTACCGCAAGTAAG<br>CGTTTCTCCGAGCCACCT	58.12<br>61.36	1,072
Sro_contig365	7898-8911	1,013	GGTACCTGTTCAGCCAGGAG<br>ATGAGGAGGTTCGAGACGAC	59.72<br>59.26	1,010
Sro_contig289	49077-50088	1,011	CAGACGTCCCGACCACAC<br>ATGGCATGTACCGGTATGCT	61.22<br>60.24	1,000
Sro_contig1430	9212-10333	1,121	AAAACCAGCGTCAAGCAC<br>GACAGCGAGACGGCAGGT	57.37<br>62.65	1,118

Supplementary Table 4. Protocol and main types of predicted repeats in *S. robusta*.

Type	Number of elements	Length occupied (bp)	Percentage of the genome (%)
SINEs	432	177,694	0.14
LINEs	4,210	1,900,567	1.51
LTR elements	10,779	11,388,646	9.05
DNA elements	2,991	1,622,259	1.29
Unclassified	27,904	12,968,335	10.31
Total interspersed repeats	46,316	28,057,501	22.31
Satellites	186	23,967	0.02
Simple repeats	1,574	391,925	0.31
Total bases masked	-	28,469,528	22.64

RepeatModeler v1.0.8 [35] was used to create a species-specific repeat library (-engine ncbi). This species-specific repeat library was inspected to identify sequences that correspond to genuine protein-coding genes (Tera-BLASTX v7.6.1 [3], e-value < 1e-05) and which were removed from the repeat library (63/429 de novo repeats were discarded). Homology-based transposable element identification was performed by the retrieval of 100 *Bacillariophyta* repeat families stored in RepBase [36] to build a homology-based repeat library. The combination of these two repeat libraries (466 repeat sequences in total) were used by the RepeatMasker v4.0.5 [37] (-nolow -norna -no_is -gff -e ncbi) to predict and mask the repetitive DNA sequences onto the final genome assembly.

Supplementary Table 5. Scores for full-length *Bacillariophyta* transcript accuracy at nucleotide level.

Metric	nt | score (%)
True positive	1,255,329
False positive	25,937
True negative	159,115
False negative	26,448
Sensitivity	97.93
Specificity	85.98
Precision	97.97
F1-measure	97.95

More information can be found in Supplementary Note 2.

Supplementary Table 6. Detailed statistics of final *S. robusta* genome assembly and gene annotation.

Genome assembly (scaffolds >1kb)	Statistic
Computationally/experimentally estimated genome size (Mb)	117-151
Nuclear genome size (bp)	125,572,603
Number of scaffolds	4,754
Longest scaffold (bp)	318,047
N50 (bp)	50,720
GC content (%)	48.32
Repeat region (%)	22.64
Classified repeats (%)	12.33
Unclassified repeats (%)	10.31
Chloroplast genome size (bp)	150,240
Mitochondrial genome size (bp)	44,018
**Gene annotation**	
Total number of nuclear protein-coding genes	36,062
Fraction of genome occupied by nuclear protein-coding genes (%)	51.64
Fraction of nuclear genes having introns (%)	41.80
Number of nuclear exons	71,783
Number of nuclear introns	35,109
Number of nuclear UTRs	27,956
Number of nuclear protein-coding genes with introns	15,074
Number of nuclear protein-coding genes with UTRs	14,634
Average number of nuclear introns in a locus*	2.33
Average nuclear locus size (bp)	1,800.89
Average nuclear exon size (bp)	853.22
Average nuclear intron size (bp)	109.69
Average nuclear intergenic spacer size (bp)	518.55
Average nuclear protein size (amino acids)	483.80
Number of nuclear protein-coding genes with isoforms	1,279
Number of nuclear RNA genes	372
Number of chloroplast protein-coding genes	146
Number of chloroplast RNA genes	40
Number of mitochondrial protein-coding genes	46
Number of mitochondrial RNA genes	26
**Functional annotation**	
Number of annotated genes with InterProScan	20,516
Number of annotated genes with EggNOG-mapper	16,519
Number of annotated genes with AnnoMine	9,304

*Only taking into account loci that have introns

Supplementary Table 7. Overview experimental conditions for *S. robusta* expression atlas.

Condition	Control[1]	#C	Treatment[1]	#T	Strain	Dur.	Reads[3]	Source
Decadienal	0.26% MeOH	3	50µM decadienal in 0.26% MeOH	3	85A	0.5h	75bp PE	New data
H2O2	Standard conditions	3	1 mM H2O2	3	85A	1h	75bp PE	New data
High salinity	34 psu	3	45 psu	3	85A	1h	75bp PE	New data
Low salinity	34 psu	3	20 psu	3	85A	1h	75bp PE	New data
Light stress (short)	40 µmol photons m-2s-1	3	450 µmol photons m-2s-1	3	85A	0.5h	75bp PE	New data
Light stress (long)	40 µmol photons m-2s-1	3	450 µmol photons m-2s-1	3	85A	6h	75bp PE	New data
Silica depletion	0.106mM Na2SiO3.9H2O	3	0mM Na2SiO3.9H2O	3	85A	24h	75bp PE	New data
Sexual reproduction (early)	Strains grown separately	3	Strains crossed	3	PONTON36 PONTON34	11h	75bp PE	New data
Sexual reproduction (middle)	Strains grown separately	3	Strains crossed	3	PONTON36 PONTON34	14h	75bp PE	New data
Sexual reproduction (late)	Strains grown separately	3	Strains crossed	2	PONTON36 PONTON34	21h	75bp PE	New data
Heat stress	Temperature 21°C	3	Temperature 30°C	3	85A	24h	75bp PE	New data
Cold stress	Temperature 21°C	3	Temperature 4°C	3	85A	24h	75bp PE	New data
Diproline	0.1x spent SIP- medium	3	0.1x spent SIP- medium + 100nM diproline	3	85A	1h	75bp PE	New data
Day / night	6h dark in 12/12 D/N	3	6h light in 12/12 D/N	3	85A	6h	75bp PE	New data
N depletion[2,5]	Natural sea water (NSW) + complete F/2	4	Natural sea water (NSW) + F/2 without nitrate	4	112-1	48h 72h	101bp SE	New data A. Bones lab
P depletion[2,5]	Natural sea water (NSW) + complete F/2	4	Natural sea water (NSW) + F/2 without phosphate	4	112-1	48h 72h	101bp SE	New data A. Bones lab
C14 AHL	0.5% DMSO	3	0.5% DMSO + 40µM non-functional AHL (C14)	3	85A	72h	75bp PE	[38]
OXO14 AHL	0.5% DMSO	3	0.5% DMSO + 40µM 3-oxo-AHL (OXO14)	3	85A	72h	75bp PE	[38]
TA14 AHL	0.5% DMSO	3	0.5% DMSO + 5µM Tetramic acid (TA14)	2	85A	72h	75bp PE	[38]
Maribacter exudates	Standard conditions	3	ASW + F/2 + spent Maribacter medium	3	84A	10h	75bp PE	[39]
Roseovarius exudates	Standard conditions	3	ASW + F/2 + spent Roseovarius medium	3	84A	10h	75bp PE	[39]
Maribacter exudates + SIP+	0.1x SIP+	3	0.1x SIP+ + spent Maribacter medium	3	84A	10h	75bp PE	[39]
Roseovarius exudates + SIP+	0.1x SIP+	3	0.1x SIP+ + spent Roseovarius medium	3	84A	10h	75bp PE	[39]
SIP- MT+ (15min)[5]	Standard conditions	3	0.1x spent SIP- medium	3	85A	15min	75bp PE	[40]
SIP- MT+ (1h)[5]	Standard conditions	3	0.1x spent SIP- medium	3	85A	1h	75bp PE	[40]
SIP- MT+ (3h)[5]	Standard conditions	3	0.1x spent SIP- medium	3	85A	3h	75bp PE	[40]
SIP- MT+ (6h)[5]	Standard conditions	3	0.1x spent SIP- medium	3	85A	6h	75bp PE	[40]
SIP- MT+ (9h)[5]	Standard conditions	3	0.1x spent SIP- medium	3	85A	9h	75bp PE	[40]
SIP+ MT- (15min)[5]	Standard conditions	3	SIP+	3	85B	15min	75bp PE[4]	[41]
SIP+ MT- (1h)[5]	Standard conditions	3	SIP+	3	85B	1h	75bp PE[4]	[41]
SIP+ MT- (3h)[5]	Standard conditions	3	SIP+	3	85B	3h	75bp PE[4]	[41]

[1] Cultures were grown in artificial sea water (ASW) enriched with Guillard's F/2, made axenic by adding an antibiotic mix described in [39] at a light intensity of 40 µmol photons $m^{-2}s^{-1}$ and a temperature of 21°C, except stated otherwise. Sample harvesting, RNA extraction and quality control were performed according to [39] except for N- and P-depletion experiments where cells were collected by centrifugation (10 min 1500g) and RNA isolation was performed as previously described in [42].

[2] Cultures were kept at 80 µmol photons $m^{-2}s^{-1}$ and 18°C for these experiments.

[3] PE = Paired-end reads, SE = Single-end reads. All new experiments were sequenced on the Illumina® HiSeq 4000 platform except for the N- and P-depletion experiments which were sequenced on the Illumina® HiSeq 2500.

[4] Reads from these samples contained adaptor sequences and were quality trimmed using Trimmomatic v0.36 [43] (ILLUMINACLIP:adapters:2:30:10).

[5] RNA-Seq experiments that were available for the first run of gene predictions.

Supplementary Table 8. Species list and genome versions used for comparative genomics analysis.

Species	Source	PubmedID
Arabidopsis thaliana	TAIR10	[44]
Aureococcus anophagefferens	JGI 1.0	[45]
Caenorhabditis elegans	ENSEMBL, release 81	[46]
Chlamydomonas reinhardtii	JGI v5.5	[47]
Chondrus crispus	ENSEMBL protists, release 28	[48]
Cyanidioschyzon merolae	Tokyo University	[49]
Cyclotella cryptica	Scripps Institution of Oceanography	[49]
Drosophila melanogaster	ENSEMBL, release 81	[50]
Ectocarpus siliculosus	Ghent University	[51]
Emiliania huxleyi	ENSEMBL protists, release 28	[52]
Fistulifera solaris	Tokyo University of Agriculture and Technology	[53]
Fragilariopsis cylindrus	JGI 1.0	[54]
Galdieria sulphuraria	ENSEMBL protists, release 28	[55]
Homo sapiens	ENSEMBL, release 81	[56]
Nannochloropsis gaditana	ENSEMBL protists, release 28	[57]
Paramecium tetraurelia	ENSEMBL protists, release 28	[58]
Phaeodactylum tricornutum	ENSEMBL protists, release 28	[59]
Physcomitrella patens	JGI v3.3	[60]
Pseudo-nitzschia multiseries	JGI 1.0	[61]
Pseudo-nitzschia multistriata	NCBI, GCA_900005105	[62]
Saccharomyces cerevisiae strain S288C	ENSEMBL, release 81	[63]
Schizosaccharomyces pombe	ENSEMBL protists, release 28	[64]
Seminavis robusta	v1.2	This study
Synedra acus	http://lin.irk.ru/sacus/	[65]
Thalassiosira oceanica	ENSEMBL protists, release 28	[66]
Thalassiosira pseudonana	ENSEMBL protists, release 28	[67]

Supplementary Table 9. InterPro domains significantly enriched for *S. robusta* tandem gene duplicates (hypergeometric distribution, multiple hypothesis testing using Benjamini–Hochberg correction, q-value < 0.05).

InterPro_id	Description	n_genes	p-value	q-value	enr_fold
IPR032675	Leucine-rich repeat domain superfamily	446	2.0428E-62	1.4255E-58	2.19978
IPR001054	Adenylyl cyclase class-3/4/guanylyl cyclase	116	5.8517E-19	1.3611E-15	2.32933
IPR029787	Nucleotide cyclase	117	4.72121E-19	1.6472E-15	2.32574
IPR011004	Trimeric LpxA-like superfamily	51	2.70109E-18	4.7121E-15	3.6924
IPR000873	AMP-dependent synthetase/ligase	56	2.70943E-16	3.7813E-13	3.17935
IPR027725	Heat shock transcription factor family	61	1.07694E-14	1.0736E-11	2.81513
IPR000232	Heat shock factor (HSF)-type, DNA-binding	62	1.27872E-14	1.1154E-11	2.77999
IPR020845	AMP-binding, conserved site	47	1.02681E-14	1.1942E-11	3.31165
IPR036736	ACP-like superfamily	42	1.88827E-14	1.464E-11	3.52603
IPR001611	Leucine-rich repeat	95	3.50957E-13	2.449E-10	2.12983
IPR036390	Winged helix DNA-binding domain superfamily	74	1.60317E-11	1.017E-08	2.22045
IPR036388	Winged helix-like DNA-binding domain superfamily	78	3.00136E-11	1.7453E-08	2.14475
IPR036865	CRAL-TRIO lipid binding domain superfamily	76	2.45934E-09	1.3201E-06	1.98596
IPR001254	Serine proteases, trypsin domain	33	4.14166E-09	2.0643E-06	2.9261
IPR001314	Peptidase S1A, chymotrypsin family	31	4.98864E-09	2.3207E-06	3.02024
IPR033116	Serine proteases, trypsin family, serine active site	25	8.74116E-09	3.8122E-06	3.40155
IPR036971	3'5'-cyclic nucleotide phosphodiesterase, catalytic domain superfamily	54	1.83414E-08	7.5286E-06	2.17422
IPR002073	3'5'-cyclic nucleotide phosphodiesterase, catalytic domain	53	3.36462E-08	1.3044E-05	2.15595
IPR003613	U box domain	35	9.94206E-08	3.6514E-05	2.534
IPR018114	Serine proteases, trypsin family, histidine active site	27	1.52522E-07	5.3215E-05	2.87937
IPR032710	NTF2-like domain superfamily	32	2.91351E-07	9.2411E-05	2.55082
IPR009003	Peptidase S1, PA clan	33	6.46633E-07	0.00018801	2.43386
IPR021838	Protein of unknown function DUF3431	9	1.28145E-06	0.00034392	5.9187
IPR003392	Protein patched/dispatched	22	1.37902E-06	0.0003564	2.94263
IPR000731	Sterol-sensing domain	22	1.91743E-06	0.00047785	2.89359
IPR006597	Sel1-like repeat	22	2.64113E-06	0.00063551	2.84615
IPR011333	SKP1/BTB/POZ domain superfamily	43	3.46936E-06	0.00080697	2.03197
IPR003607	HD/PDEase domain	45	5.68719E-06	0.00128017	1.962
IPR009081	Phosphopantetheine binding ACP domain	18	1.12826E-05	0.00238575	2.95935
IPR001320	Ionotropic glutamate receptor	17	2.45893E-05	0.0049024	2.91646
IPR000210	BTB/POZ domain	36	3.50232E-05	0.00643136	1.9867
IPR009959	Polyketide cyclase SnoaL-like	11	4.16033E-05	0.0074438	3.77424
IPR012292	Globin/Protoglobin	19	4.47353E-05	0.00752201	2.63053
IPR009050	Globin-like superfamily	19	4.47353E-05	0.00752201	2.63053
IPR001036	Acriflavin resistance protein	7	4.41278E-05	0.00769809	5.52412
IPR035992	Ricin B-like lectins	16	9.89559E-05	0.0140921	2.7449
IPR001905	Ammonium transporter	7	0.000107995	0.0150717	5.02193
IPR000772	Ricin B, lectin domain	17	0.000145741	0.0199408	2.57995
IPR017452	GPCR, rhodopsin-like, 7TM	12	0.00015951	0.0208049	3.15664
IPR000971	Globin	12	0.00015951	0.0208049	3.15664
IPR001828	Receptor, ligand binding region	25	0.000248374	0.029882	2.07674
IPR018047	Ammonium transporter, conserved site	6	0.00024594	0.0301083	5.26107

Supplementary Table 10. A selection of young families with strong expression divergence and functional annotation.

Family id	Annotation	Age	ED%[1]	EC%[1]	SED%[1]	SEC%[1]
HOM02SEM016602	5'-Nucleotidase, C-terminal	Diatom	100	0	50	0
HOM02SEM004823	Acyl transferase/acyl hydrolase/lysophospholipase	Diatom	66.67	33.33	16.67	0
HOM02SEM005985	AdipoR/Haemolysin-III-related	Species-specific	66.67	33.33	8.33	0
HOM02SEM022603	AhpD-like	Species-specific	100	0	100	0
HOM02SEM013092	BTB/POZ domain	Diatom	75	25	25	0
HOM02SEM006207	calcium ion binding	Diatom	100	0	25	0
HOM02SEM028963	calcium ion binding	Species-specific	100	0	50	0
HOM02SEM004128	Calycin	Diatom	100	0	50	0
HOM02SEM009951	Chondroitin N-acetylgalactosaminyltransferase	Diatom	100	0	25	0
HOM02SEM000073	CRAL-TRIO lipid binding domain superfamily	Diatom	75	25	5	0
HOM02SEM004442	Cyclophilin-like domain superfamily	Diatom	100	0	50	0
HOM02SEM002750	Dynamin superfamily	Diatom	81.82	18.18	9.09	0
HOM02SEM003493	EF-hand domain	Diatom	66.67	33.33	33.33	0
HOM02SEM000539	Epidermal growth factor receptor ligand	Diatom	68.89	31.11	6.67	0
HOM02SEM008992	extracellular region	Species-Specific	83.33	16.67	16.67	0
HOM02SEM011409	Fatty acid desaturase domain	Diatom	100	0	100	0
HOM02SEM011524	Fatty acid hydroxylase	Diatom	100	0	50	0
HOM02SEM005625	Flavodoxin-like	Diatom	100	0	100	0
HOM02SEM009201	fucosyltransferase activity	Diatom	60	40	20	0
HOM02SEM020466	gamma-glutamylcyclotransferase activity	Diatom	100	0	100	0
HOM02SEM004062	Glycoside hydrolase family 20	Diatom	60	40	20	0
HOM02SEM004401	Heat shock chaperonin-binding	Diatom	100	0	100	0
HOM02SEM000767	Histidine kinase domain	Diatom	60.87	39.13	4.35	0
HOM02SEM015186	Lactonase, 7-bladed beta propeller	Diatom	100	0	100	0
HOM02SEM013935	Leucine-rich repeat domain superfamily	Species-specific	100	0	50	0
HOM02SEM001429	LRAT-like domain	Diatom	87.5	12.5	12.5	0
HOM02SEM025549	magnesium ion binding	Species-specific	100	0	100	0
HOM02SEM024069	NodB homology domain	Species-specific	100	0	100	0
HOM02SEM005732	Nucleotide-diphospho-sugar transferases	Diatom	100	0	100	0
HOM02SEM006518	P-loop containing nucleoside triphosphate hydrolase	Diatom	100	0	50	0
HOM02SEM012423	P-loop containing nucleoside triphosphate hydrolase	Species-specific	80	20	20	0
HOM02SEM004365	PDZ domain	Diatom	76.92	23.08	15.38	0
HOM02SEM005910	Pentatricopeptide repeat	Diatom	100	0	100	0
HOM02SEM000133	Periplasmic binding protein-like I	Diatom	84.78	15.22	1.09	0
HOM02SEM002552	phosphotyrosine residue binding	Diatom	66.67	33.33	16.67	0
HOM02SEM003282	Potassium channel domain	Diatom	100	0	11.11	0
HOM02SEM006796	Prenylated rab acceptor PRA1	Diatom	75	25	25	0
HOM02SEM010924	protein homooligomerization	Diatom	60	40	20	0
HOM02SEM001099	Protein phosphatase 2C family	Diatom	100	0	20	0
HOM02SEM022281	RDD	Species-specific	100	0	33.33	0
HOM02SEM004278	S-adenosyl-L-methionine-dependent methyltransferase	Diatom	100	0	50	0
HOM02SEM004782	SGNH hydrolase superfamily	Diatom	76.92	23.08	7.69	0
HOM02SEM006582	SGNH hydrolase-type esterase domain	Diatom	66.67	33.33	33.33	0
HOM02SEM008774	SGNH hydrolase-type esterase domain	Diatom	100	0	100	0
HOM02SEM009716	Signal recognition particle receptor	Diatom	100	0	100	0
HOM02SEM005193	sulfotransferase activity	Diatom	100	0	33.33	0
HOM02SEM017209	TFIIH p62 subunit, N-terminal	Species-specific	75	25	25	0
HOM02SEM001343	TLDc domain	Diatom	80	20	20	0
HOM02SEM007113	Transmembrane protein 180	Diatom	100	0	50	0
HOM02SEM016144	ubiquitin-protein transferase activity	Species-specific	100	0	50	0
HOM02SEM009237	WW domain	Diatom	100	0	100	0

[1] ED=Expression divergence, EC=Expression conservation, SED=Strong expression divergence, SEC=Strong expression conservation.
Source data are provided as a Source Data file.

Supplementary Table 11. Overview of the tools used to build PLAZA Diatoms 1.0.

Tool version	Task	Parameters	Reference
DIAMOND v0.9.18.119	all-against-all protein sequence similarity search	max #hits 4000, e-value < 10e-05	[68]
TribeMCL v10-201	large-scale detection of gene families	default	[69]
MAFFT v7.187	multiple sequence alignment of protein gene families	default	[18]
FastTree v2.1.7	approximate-maximum-likelihood phylogenetic trees of protein gene families	default	[70]
RaxML v8.2.9	diatom species tree inference	model PROTGAMMAWAG, 100 bootstraps	[71]
i-ADHoRe v3.0	detection of gene duplication types and inference collinear regions	alignment method: gg2, gap size 15, tandem gap 15, cluster gap 15, q-value 0.85, probability cut-off 0.01, anchor_points 3, level_2_only FALSE, FDR as method for multiple hypothesis correction	[72]

Supplementary Note 1. DNA isolation techniques and flow cytometry

For Illumina DNA sequencing, cell cultures of *S. robusta* D6 strain were grown in F/2 medium [73] made with filtered (GF/C grade microfiber filter; Whatman) autoclaved seawater collected from the North Sea and supplemented with 100 µg/ml penicilline, 100 µg/ml streptomycine, 100 µg/ml gentamycine and 100 µg/ml imipenem. The cultures were grown at 18°C with a 12:12-h light:dark regime and approximately 85 µmol photons m^{-2} s^{-1} from cool-white fluorescent lights. For DNA extraction, cells were collected on Versapor -3,000T membrane filter (PallLife Sciences) and washed once with 1xPBS. Cells were frozen at -80°C until processing. Cell lysis was achieved by mechanical disruption in 400 mL of AP1 DNeasy buffer (Qiagen) by beating with glass/zirconium beads (0.1 mm diameter; Biospec) on a bead mill (Retsch) with frequency 20/s for 3 x 1min. Next the DNA was extracted with DNeasy Plant Mini Kit (Qiagen) according to manufacturer's instructions. Due to a low 260/230 absorption ratio it was in some cases necessary to further purify extracted DNA with chloroform. An equal volume of chloroform was added to the DNA and mixed thoroughly. The mixture was centrifuged at 10,000 rpm for 5 minutes at 4°C. The aqueous upper phase was transferred to a new tube and DNA was precipitated by addition of 1/10 of volume of 3M NaAc pH5.2 and 0.7 volume of isopropanol. DNA was pelleted by 30 minutes centrifugation at 14 000 rpm at 4°C and washed with 70% ethanol. DNA was resuspended in 20µl of Tris-HCl pH8.5. DNA library preparation was done at VIB Nucleomics Core with Illumina True seq kit from 500 ng of genomic DNA. DNA spiked by 1.4% addition of PhiX was sequenced on a MiSeq sequencing platform using MiSeq Reagent Kit v3 (600-cycles) (Illumina).

For PacBio DNA sequencing, CTAB DNA extraction was followed by high salt wash in order to remove impurities that are frequently present in DNA extraction preparations from *S. robusta*. In brief, the cell pellet was resuspended in 400 µl of CTAB buffer (1% (w/v) CTAB, 100 mM Tris-HCl pH7.5, 10 mM EDTA pH8, 700 mM NaCl and freshly added 4 µg of RNase A) and cells were disrupted by agitation with glass/zirconium beads (0.1 mm diameter; Biospec) on a bead mill (Retsch) for 3 times during 1 minute. Samples were then incubated for 30 min at 60°C and let to cool down on ice for 15 minutes. Afterwards, 250 µl of chloroform:isoamylalcohol 24:1 was added and the samples were mixed manually for 1 minute. Phases were separated by centrifugation at 20000 x g for 10 minutes. The upper aqueous phase was transferred to a new tube and DNA was precipitated by addition of an equal volume of isopropanol followed by centrifugation for 15 minutes at 20,000 x g. The DNA pellet was washed with 70% ethanol, air-dried and resuspended in 20 µl of Tris-HCl pH8.5. If the purity of DNA was not sufficient for PacBio sequencing, samples were cleaned by high-salt wash. First, DNA sample was mixed with 378 µl Tris-HCl pH8.5, 100 µl 5M NaCl and 2 µl of EDTA. Then the DNA was extracted by 400 µl of Phenol:Chloroform:Isoamylalcohol 25:24:1. Samples were gently mixed and centrifuged for 10 minutes at 20000 x g. The aqueous phase was transferred to a new tube and mixed with an equal volume of chloroform:isoamylalcohol 24:1. Samples were again gently mixed and centrifuged for 10 minutes at 20 000 x g. The aqueous phase was transferred to a new tube and mixed with 0.3x volume of 100% ethanol. This high salt, low ethanol mixture precipitates polysaccharides while genomic DNA (gDNA) stays in solution. Samples were centrifuged for 15 minutes at 20 000 x g. Supernatant was transferred to a new tube and DNA was precipitated by addition of 1.7 x volume of 100% ethanol. After another centrifugation for 15 minutes at 20000 x g, the supernatant was removed, and the pellet was washed twice 70% ethanol. Pellet was air-dried and resuspended in

20µl of Tris-HCl pH8.5. Library preparation at Nucleomics VIB core facility was done with the "SMRTbell Template Prep Kit 1.0" (100-259-100). At the end, size selection was done with the Blue Pippin, using the "High Pass Protocol V3" protocol with a "0.75% PAC20KB" cassette. After elution, possible nicks were repaired with the "SMRTbell Damage Repair Kit" (100-465-900). Eleven SMRT cells were sequenced on the RSII with P6-C4 chemistry.

In order to estimate the genome size of *S. robusta* by flow cytometry, *S. robusta* was compared to an internal standard of *P. tricornutum* and *F. cylindrus*. Cultures were grown in NSW + Guillard's F/2 and subsequently fixated in 1% formaldehyde solution for 1h. Afterwards, cultures were suspended in ice-cold methanol for > 24h to remove pigments. Samples were stained in a 0.1µg/mL solution of 4,6-diamidino-2-phenylindole (DAPI) and analyzed on a CyFlow ML flow cytometer (Sysmex - Partec). The ratio of DAPI intensity of the *S. robusta* peak in the histogram versus the internal standard was calculated and averaged. In total, 20 comparisons using *P. tricornutum* and eight using *F. cylindrus* as a standard were performed and average value including standard deviation is reported.

Supplementary Note 2. Assessment of genome assembly and gene model quality

To polish the final genome assembly, illumina paired-end reads were aligned using BWA-MEM v0.7.5a [13] (default parameters), duplicated reads were marked using Picard v2.6.0 [74] and indels were locally re-aligned using the –IndelRealigner command from GATK v3.7.0 [75]. Then, a recalibration of per-base alignment quality was performed using -mpileup command from SAMtools [76], followed by a variant calling with bcftools v1.3 [77]. The resulting variants were filtered, keeping the SNPs/INDELs that had more than one read coverage and where the alternative allele frequency was more than 0.7. The assembly sequence correction was executed using the –FastaAlternateReferenceMaker command from GATK with this filtered variant dataset.

The contiguity of the final reference assembly was assessed using QUAST v4.4 [78]. Quality metrics used include the retrieval of 91% of Illumina reads that uniquely mapped to the assembly and 85% that mapped concordantly using SAMtools (F=4 and q=1 for unique mapping and –flagstat for concordant statistics). The mapping of 114 in-house generated Sanger-sequenced PCR fragments using BLASTn search (>=85% identity and >=95% coverage) also showed that all these sequences were present in the assembly. The gene space completeness of the assembly was assessed by mapping 10,056 *S. robusta* assembled transcripts (with *Bacillariophyta* taxonomy) using GMAP v2016-04-04 [79] with default parameters (>=85% identity and >=95% coverage), revealing that more than 99% of these assembled transcripts [18] were found, of which 95% were fully covered. A summary of the contiguity and quality metric results can be found in Supplementary Table 2. The final genome assembly was also experimentally validated by the PCR amplification of 22 regions of interest, corresponding to regions that contain transcript evidences well-supported by Illumina and/or PacBio reads but were missing from the other computed assemblies showing better contiguity (Supplementary Figure 3A-D and Supplementary Table 3). Together, these results confirmed the good quality of the obtained assembly and indicated that more aggressive assemblies, frequently yielding higher N50 values, did not offer better quality (Supplementary Table 2*)*.

The accuracy and completeness of the gene annotation v1.2 was assessed by the implementation of three extra evaluation strategies. In the first approach, the previously referred *Bacillariophyta* assembled transcripts used to validate the assembly (10,056) were uploaded into TRAPID v1 [80] to select those transcripts annotated as full-length. Next, these full-length transcripts were mapped to the *Phaeodactylum tricornutum* proteome using a Tera-BLASTX sequence similarity search [3] (maximum e-value 10e-10) to retrieve those transcripts having a similar length in this related diatom. This subset of 615 transcripts was used as a gold standard to evaluate the accuracy of the protein-coding gene predictions. Thus, these transcripts were aligned to the final genome assembly using GMAP v2016-04-04 [79] and the resulting aligned regions were compared at the nucleotide level with the coding sequence (CDS) predicted by BRAKER using Bedtools v2.26.0 [15], yielding 98.54% sensitivity and 83.82% specificity (Supplementary Table 5). In the second approach, the presence of 3,881 *Bacillariophyta* core Gene Families (coreFamilies) was assessed, as described by Van Bel and co-workers [81], recovering 3,832 (98.62%) of these highly conserved coreFamilies (Figure 1B). In short, coreFamilies were defined as all gene families present in all three well-annotated diatom species (*Phaeodactylum tricornutum*, *Thalassiosira pseudonana* and *Fragilariopsis cylindrus*) stored in the pico-PLAZA database [82] and were searched in the predicted proteins of *S. robusta* using DIAMOND [68] (e-value < 10e-5). Then, the number of represented coreFamilies was calculated and the same analysis was performed for other published diatom species (Figure 1B). Whereas CEGMA or BUSCO focus on eukaryotic single-copy genes, the gene completeness strategy used here is based on *Bacillariophyta* coreFamilies which is more specific, as it covers a set of reference genes ~5 to 10 times higher compared to the BUSCO or CEGMA gene sets, respectively, and shows less functional biases compared to e.g. BUSCO [83]. In the third approach, for 16,204 diatom gene families consisting out of at least 3 and at most 1,000 members, the protein-based multiple sequence alignment of homologous genes was evaluated to identify potential partial and merged genes in all published diatom species. In total, 26,734 *S. robusta* protein-coding genes were assessed, yielding 5.93% potentially partial and 1.00% merged gene models (Supplementary Figure 6). All the different scores reported in this Supplementary Note 2 demonstrate the high quality of the gene models and also indicate that, compared to other diatoms with large genomes, the *S. robusta* genome assembly and gene annotation is of good quality. Detailed statistics of the final *S. robusta* genome assembly and gene annotation can be found in Supplementary Table 6.

Supplementary Note 3. Gene family and gene age content comparison across several diatoms

Diatoms with the largest number of protein-coding genes (*S. robusta*, *T. oceanica* and *S. acus*, Figure 1B) have overall more families (for both small/large sizes) compared to smaller diatoms such as *P. tricornutum*, albeit they do not display a bias towards to any specific family size (Supplementary Figure 7).

For each family, the age was calculated through a phylostratification approach which determines the taxonomic scope of the homologous genes within a family. This analysis showed that diatoms with large genomes tend to have more species-specific families and the largest proportion of species-specific single-copy families (Supplementary Figure 8). More than half of the *S. robusta*

genes are relatively young (36.74% species-specific and 24.86% diatom, Figure 1C), which is comparable to the proportions found in *T. oceanica* (42.02% species-specific and 22.93% diatom) or *S. acus* (30.91% species-specific and 26.68% diatom), but it contrasts with its closest relative, *P. tricornutum*, where more than half of the genes are older (56.95% either Eukaryota or Stramenopile).

Functional Gene Ontology (GO) enrichment for different gene age categories reveals some distinct known and novel patterns (Supplementary Figure 9). Genes exclusive to diatoms displayed enrichment for diatom-life style features, such as 'silicic acid transporters' that are required to build their silica cell wall [84], 'cAMP catabolic process' linked to carbon dioxide detection in the ocean surface [85] and 'G protein-coupled GABA receptor' already previously identified in diatoms showing expression under various stress conditions [86]. *S. robusta* species-specific genes were enriched in 'establishment of meiotic spindle localization', suggesting that new families have evolved in this species related to its sexual reproduction, as well as 'polyketide metabolic process', although the role of these secondary metabolites still needs to be clarified.

Supplementary Note 4. Examples of *S. robusta* gene family expansions with a potential role in molecular sensing, light signaling and motility

The *S. robusta* genome possess a large number of G protein-coupled receptors (GPCRs), including the 'GPCR family 3, GABA-B receptor' (46/221 tandem copies) and 'GPCR, rhodopsin-like, 7TM' (12/30 tandem copies). A recent study has proposed that GABA GPCRs could help to mediate the sensing of high temperatures in diatoms [87]. Although we found 23 *S. robusta* GABA GPCRs upregulated at high temperature, many others showed upregulation in a wide range of stresses, suggesting these cell-surface receptors putatively play a more general role in environmental responses. Notably, we found a pennate-specific cysteine-rich secretory protein, antigen 5, pathogenesis-related 1 proteins (CAP) family, also expanded by tandem duplication (5/9 tandem copies), which has not been previously described in diatoms. Despite its conserved structure, the role of the CAP domain in relation to the diverse functions of these proteins is poorly understood, albeit they are most often secreted and have a role in cell-cell adhesion, signaling, or immune regulation in host-pathogen interactions [88].

Raphid benthic diatoms employ motility in response to resource availability such as light and nutrients as well as for escaping from physical disturbance or searching mating partners. We found the single-domain voltage-gated channels (EukCatAs) family expanded, which corresponds to an alternative mechanism for fast Na+/Ca2+ signaling that has been shown to modulate gliding locomotion in raphid pennate diatoms [89]. However, the presence of these proteins in non-motile diatom species suggests they may also contribute to other diverse signaling processes, which agrees with the broad expression pattern we observed. Another interesting family expansion is the red/far-red light sensing phytochrome (DPH), which was previously identified in centric diatoms as single-copy and contains up to four genes in other pennate diatoms (e.g., *S. robusta* and *A. coffeaeformis*) [90]. Our analysis shows that *S. robusta*'s DPH expansion is driven by tandem duplication (3/4 tandem copies). Although far-red wavelengths from sunlight are only detectable at the ocean surface, chlorophyll fluorescence can generate red/far-red photons, giving the

possibility that DPH proteins act as a biotic signal detector by sensing elevated chloroplast fluorescence from nearby cells [90]. Therefore, the DPH family expansion could be involved in *S. robusta*'s properties to detect density and stress status of the biofilms it inhabits.

Supplementary Note 5. Examples of *S. robusta* gene families with specific expression towards reproduction, high temperature and bacterial interaction experiments

S. robusta has been proposed as a model to study sexual reproduction in diatoms, which is an essential phase in their unique life cycle characterized by cell size reduction during vegetative growth, pheromone signaling and auxospore formation [39, 41, 91]. We found 42 reproductive responsive families, of which 23 are significantly enriched in the three sexual stages available (Figure 3D). More than half of the reproductive families is expanded and 17% is enriched in tandem duplicates. Interestingly, the majority of families are either species- or diatom-specific (70%) and many have no known functional domains, suggesting a role in some of the unique sexual traits of the life cycle of *S. robusta* and/or diatoms. Four different reproduction families encode proteins with a BTB/POZ domain, which is generally involved in protein/protein interactions in various cellular processes ranging from ion channel assembly to the targeting of proteins for ubiquitination [92]. Accordingly, we also identified U box ubiquitin ligases containing Sel1 repeats responsive to reproduction and expanded by tandem duplication. Interestingly, the *S. robusta* cyclin A/B family is also part of the reproductive responsive families. Out of 10 A/B-type cyclins, four are significantly upregulated in all stages of the mating process but, in contrast to other genes from this family, they are not differentially expressed in day-night transition, suggesting they play a specific regulatory role during sexual reproduction (Supplementary Figure 14). Finally, two reproductive families are potential candidates for cell-cell recognition of gametangia and/or fusion of gametes: genes characterized by an N-terminal domain homologous with the alpha subunit of integrins, which are in mammals involved in cell-cell and cell-matrix communication, and metallopeptidases belonging to the M12B subfamily, whose M12B domain is also present in the mammalian ADAM2 gene, a sperm surface membrane protein hypothesized to be involved in sperm-egg adhesion and fusion [93].

S. robusta is found in shallow coastal habitats that can experience extreme temperature changes, especially during heatwaves which are expected to increase in their frequency, duration and intensity due to the climate change [94]. We found eight families with strong expression bias towards high temperature that could be involved in *S. robusta*'s temperature acclimatization (Supplementary Figure 17). Although half of these families are species-specific and/or have no functional annotation, we could identify three annotated tandem-enriched families, one as ionotropic glutamate receptors (iGluRs) and two with the leucine-rich repeat (LRR) domain. iGluRs have been reported in a wide range of photosynthetic organisms, where they appear to control physiological processes such as carbon/nitrogen sensing or fungal resistance [95]. Although our expression data shows upregulation of some of these genes in a variety of abiotic stresses (Supplementary Figure 18), the strong differential expression pattern suggests they are particularly important for high temperature sensing. LRR domains are considered to mediate general protein-protein interactions and in particular, previous studies have shown that receptors containing LRR domains are involved in the interaction of *P. tricornutum* with the dinoflagellate *A.*

tamarense [96] as well as the bacterium *Roseovarius* [97]. However, the observed enrichment for upregulation during high temperature of these families indicates that LRR-like proteins could be also strongly relevant for sensing environmental abiotic stresses.

Another important ecological aspect of *S. robusta* is its abundance in subtidal biofilm communities. We identified 29 families showing an expression bias towards bacterial exudates, 31 towards bacterial acyl homoserine lactones (AHLs) exposure (Figure 3C and Supplementary Figure 16B) and 11 towards conditions from both bacterial interaction experiments (Supplementary Figure 16C). Whereas more than half of these families are species-specific and/or have no functional annotation, the other families are involved in intracellular signaling, oxygen sensing, detoxification and oxidative stress responses [39, 98]. We observed a tandem-enriched family expansion of Arf GTPase-activating (GAPs) proteins having expression bias for 5/7 of the bacterial interaction conditions (Supplementary Figure 16C). Arf GAPs function as regulators of specialized membrane surfaces implicated in cell migration [99]. Together with their strong upregulation in our bacterial interaction experiments, we suggest these proteins are potential candidates to be involved in cell adhesion and movement during biofilm formation.

Supplementary Note 6. Generation of a high confidence coding SNP dataset

GATK hard-filters were applied to the raw single nucleotide polymorphism (SNP) dataset using the following parameters (QD <235 2, FS > 60, MQ < 40, MQRankSum < -12.5, ReadPosRankSum < -8) and VCFtools v0.1.16 [100] was used to apply extra additional filters as follows. SNPs were filtered out by a minimum quality score of 30 (QUAL < 30) and regions containing transposable elements were also removed. The mean depth across all SNPs per individual was computed yielding 3-11 mean read coverage. Giving this low depth of our data, we decided to further filter our SNPs by a minimum read depth of 3 (--minDP 3) as well as a maximum read depth of 30 (--maxDP 30). Since *S. robusta* is a diploid organism, we did not have enough power to distinguish homozygous from heterozygous SNPs, hence GATK was employed to retain only biallelic 'homozygous' SNPs, obtaining 5,154,628 SNPs in total. These SNPs were functionally annotated using snpEff v4.3t [101] and minor allele frequency was computed using VCFtools. The highest proportions of nonsense mutations were found at lower allele frequency whereas synonymous variants tended to have higher proportions at higher allele frequencies, being consistent with purifying selection acting against mutations that prematurely truncate the protein (Supplementary Figure 2*1*).

Prior computing π_N / π_S ratios, we further filtered out these SNPs to have a high-confidence SNP dataset in coding regions. To do so, alignments of the 48 different *S. robusta* strains were processed using SAMtools mpileup [77] to calculate callable positions with a minimum read depth of 3 across the complete D6 reference genome. Then, *S. robusta* strains that had at least 80% of reference CDS as callable positions were selected, retaining 13 strains in total (5 from clade I and 8 from clade II according to [102] classification) that cover 31 Mb of callable CDS common for all of them. Next, for each gene present in these 31 Mb, we computed the gene length coverage and retained genes that covered >= 50% of their CDS length, accounting for 28 Mb covering 21,086 genes. Note that to compute the gene length coverage, we considered only CDS as callable positions when the complete codon was callable, since this is relevant for π_N / π_S calculation. The vast majority of the selected genes contained SNPs (20,891 in total). The SNP dataset was filtered out using VCFtools to keep only the genotype information for the selected 13 strains and the SNPs

within these 28 Mb of callable CDS, generating a final high-confidence SNP dataset of 759,006 SNPs in which synonymous variants (496,109 SNPs) were twice as numerous as the non-synonymous ones (261,685 SNPs) and nonsense variants were a vast minority (1,212 SNPs).

Supplementary Note 7. Examples of genes with high pennate and/or raphid signature potentially mediating differences in cell symmetry and motility

Actin is an abundant component of the cytoskeleton that forms filaments and plays a fundamental role in processes that include motility, membrane transport, and control of cell morphology. Most eukaryotic cells also contain a varying number of actin-related proteins (ARPs) that are either located in the cytoplasm (ARP1-3, ARP10) or nucleus (ARP4-9) [103]. Our analysis shows that ARP10 proteins cluster in separate centric and pennate families (Supplementary Figure 23). ARP10 proteins form part of the dynactin complex that allows dynein-mediated movement as well as plays an important role in for example nuclear migration during mitosis [104]. Although the majority of dynactins functions are in conjunction with cytoplasmic dynein, it has been proven that this complex also binds to and modulates kinesin proteins [104]. Thus, the additional identification of a pennate-specific microtubule motor kinesin (Supplementary Figure 24) suggests that the differences in these proteins between diatom clades could lead to specific interactions. Moreover, we also observed differences in CLASP N-terminal proteins that are implicated in the attachment of microtubules to the cell cortex, regulating their stability (Supplementary Figure 25), and in Tubulin-tyrosine ligase/Tubulin polyglutamylase (TTL / TTLL) proteins involved in the post-translational modification of tubulins which make up microtubules (Supplementary Figure 26). In the Antarctic ciliate *Euplotes focardii*, alpha-tubulin polyglutamylation is implicated in the interaction between tubulin and motor microtubule-associated proteins whereas in contrast, beta-tubulin phosphorylation may play a determinant role in the dynamic of polymerization and depolymerization at low temperatures [105]. The *S. robusta* pennate-specific TTL/TTLL protein is upregulated during low temperature, suggesting that pennate species might have specific tubulin post-translational modifications to cope with this environmental perturbation.

In addition, we also identified genes with high pennate signature intrinsically related to cell membrane composition such as a CAAX amino terminal protease (Supplementary Figure 27) and a fatty acid desaturase (Supplementary Figure 28). The former *S. robusta* gene shows strong upregulation during early sexual reproduction, indicating these proteases may be relevant during cell pairing between opposite mating types, especially since they are inserted in the bilayer structure of the membrane and shown to be potentially implicated in protein and/or peptide modification and secretion [106]. In contrast, the *S. robusta* fatty acid desaturase is upregulated during low temperatures, probably to catalyze the conversion to double bonds of fatty acyl chains so as to allow the membrane to become more fluid for cold adaptation. Apart from these cytoskeleton and membrane associated proteins, we identified genes with high pennate signature holding a ‘histidine kinase’, ‘leucine-rich repeat’, ‘P domain’ (8/13 co-occurring together with ‘kexin/furin catalytic’ domain, marking subtilisin peptidases) or ‘ubiquitin’ domain, which also suggests the presence of specific signal transduction pathways linked to pennate identity (Figure 5).

Raphid signature genes are enriched for proteins containing ‘peptidase C2, calpain’, intracellular proteases predominantly involved in cellular functions that are regulated by calcium [107]. Raphid species are responsive to intracellular calcium, playing a role in switching mechanism that leads to reversal of their locomotion machinery [108]. Interestingly, one of the possible functions of calpains includes the regulation of cell migration by controlling the dynamics of both integrin-mediated adhesion and actin-based membrane protrusion, enabling cell movement by modifying these adhesion sites [109, 110]. Although calpain proteins also appear to exist in centric and araphid species, phylogenetic analysis revealed that all the calpains showing high raphid signature cluster together in one separated raphid-specific family (Supplementary Figure 30). This suggests that these proteins contain extra motifs that differentiate them from their non-raphid counterparts and therefore are potential candidates to be implicated in raphid cell movement regulated by calcium. Another example of a gene showing high raphid signature includes an ornithine decarboxylase (Supplementary Figure 31) which is upregulated during early and middle reproduction. This suggests there are unique enzymes in raphid species related to their cell wall synthesis, since the inhibition of ornithine decarboxylase has been shown to result in dramatic alteration of diatom silica structure [111]. Next to this, several raphid-specific membrane transporters were found, as well as proteins containing the ‘Rab-GTPase-TBC’, ‘exocyst complex component EXOC3/Sec6’ or ‘target SNARE coiled-coil’ domain (Supplementary Figure 32). The latter suggests the presence of specific pathway components in raphid diatoms related to vesicle trafficking [112]. Another protein that showed a prominent raphid signature (present in 14/18 raphid species in MMETSP) being part of a specific raphid family (present in all 6 raphid genomes) was annotated with an NmrA-like domain. Whereas NmrA is a negative transcriptional regulator reported to be involved in the global response to low nitrogen [113], our expression data suggests that this raphid-specific protein could have a more general function.

Supplementary References

1. Marcais G, Kingsford C. A fast, lock-free approach for efficient parallel counting of occurrences of k-mers. *Bioinformatics* **27**, 764-770 (2011).

2. Vurture GW, *et al.* GenomeScope: fast reference-free genome profiling from short reads. *Bioinformatics* **33**, 2202-2204 (2017).

3. TimeLogic. Tera-BLAST algorithm (Active Motif Inc., Carlsbad, CA.).

4. Brembu T, Winge P, Tooming-Klunderud A, Nederbragt AJ, Jakobsen KS, Bones AM. The chloroplast genome of the diatom *Seminavis robusta*: new features introduced through multiple mechanisms of horizontal gene transfer. *Marine Genomics* **16**, 17-27 (2014).

5. Oudot-Le Secq MP, Green BR. Complex repeat structures and novel features in the mitochondrial genomes of the diatoms *Phaeodactylum tricornutum* and *Thalassiosira pseudonana*. *Gene* **476**, 20-26 (2011).

6. An SM, Noh JH, Lee HR, Choi DH, Lee JH, Yang EC. Complete mitochondrial genome of biraphid benthic diatom, *Navicula ramosissima* (Naviculales, Bacillariophyceae). *Mitochondrial DNA Part B* **1**, 549-550 (2016).

7. Tillich M, *et al.* GeSeq - versatile and accurate annotation of organelle genomes. *Nucleic Acids Research* **45**, W6-w11 (2017).

8. Greiner S, Lehwark P, Bock R. OrganellarGenomeDRAW (OGDRAW) version 1.3.1: expanded toolkit for the graphical visualization of organellar genomes. *Nucleic Acids Research*, **47**, W59-W64 (2019).

9. Burge S, *et al.* Manual GO annotation of predictive protein signatures: the InterPro approach to GO curation. *Database* **2012**, bar068 (2012).

10. Huerta-Cepas J, *et al.* Fast genome-wide functional annotation through orthology assignment by eggNOG-Mapper. *Mol Biol Evol* **34**, 2115-2122 (2017).

11. Burglin TR. The Hedgehog protein family. *Genome Biology* **9**, 241 (2008).

12. Nielsen H. Predicting secretory proteins with SignalP. *Methods Mol Biol* **1611**, 59-73 (2017).

13. Li H. Aligning sequence reads, clone sequences and assembly contigs with BWA-MEM. Preprint at http://arxiv.org/abs/1303.3997 (2013).

14. Chaisson MJ, Tesler G. Mapping single molecule sequencing reads using basic local alignment with successive refinement (BLASR): application and theory. *BMC Bioinformatics* **13**, 238 (2012).

15. Quinlan AR, Hall IM. BEDTools: a flexible suite of utilities for comparing genomic features. *Bioinformatics* **26**, 841-842 (2010).

16. Dunn CW, Zapata F, Munro C, Siebert S, Hejnol A. Pairwise comparisons across species are problematic when analyzing functional genomic data. *Proc Natl Acad Sci U S A* **115**, E409-e417 (2018).

17. Kryuchkova-Mostacci N, Robinson-Rechavi M. A benchmark of gene expression tissue-specificity metrics. *Brief Bioinform* **18**, 205-214 (2017).

18. Katoh K, Standley DM. MAFFT multiple sequence alignment software version 7: improvements in performance and usability. *Mol Biol Evol* **30**, 772-780 (2013).

19. Capella-Gutierrez S, Silla-Martinez JM, Gabaldon T. trimAl: a tool for automated alignment trimming in large-scale phylogenetic analyses. *Bioinformatics* **25**, 1972-1973 (2009).

20. Nguyen LT, Schmidt HA, von Haeseler A, Minh BQ. IQ-TREE: a fast and effective stochastic algorithm for estimating maximum-likelihood phylogenies. *Mol Biol Evol* **32**, 268-274 (2015).

21. Rambaut A. FigTree v1.4 (Tree Figure Drawing Tool.) (2009).

22. Nakov T, Beaulieu JM, Alverson AJ. Accelerated diversification is related to life history and locomotion in a hyperdiverse lineage of microbial eukaryotes (Diatoms, Bacillariophyta). *New Phytol* **219**, 462-473 (2018).

23. UniProt: a worldwide hub of protein knowledge. *Nucleic Acids Research* **47**, D506-d515 (2019).

24. Valle KC*, et al.* System responses to equal doses of photosynthetically usable radiation of blue, green, and red light in the marine diatom *Phaeodactylum tricornutum*. *PLoS ONE* **9**, e114211 (2014).

25. Ritchie ME*, et al.* limma powers differential expression analyses for RNA-sequencing and microarray studies. *Nucleic Acids Research* **43**, e47 (2015).

26. JGI. (BBTools.) (2014).

27. Zhang J, Kobert K, Flouri T, Stamatakis A. PEAR: a fast and accurate Illumina Paired-End reAd mergeR. *Bioinformatics* **30**, 614-620 (2014).

28. Salmela L, Rivals E. LoRDEC: accurate and efficient long read error correction. *Bioinformatics* **30**, 3506-3514 (2014).

29. Kajitani R*, et al.* Efficient *de novo* assembly of highly heterozygous genomes from whole-genome shotgun short reads. *Genome Res* **24**, 1384-1395 (2014).

30. Chin CS*, et al.* Nonhybrid, finished microbial genome assemblies from long-read SMRT sequencing data. *Nature Methods* **10**, 563-569 (2013).

31. Koren S, Walenz BP, Berlin K, Miller JR, Bergman NH, Phillippy AM. Canu: scalable and accurate long-read assembly via adaptive k-mer weighting and repeat separation. *Genome Res* **27**, 722-736 (2017).

32. Chin CS*, et al.* Phased diploid genome assembly with single-molecule real-time sequencing. *Nature Methods* **13**, 1050-1054 (2016).

33. Ye C, Hill CM, Wu S, Ruan J, Ma ZS. DBG2OLC: efficient asembly of large genomes using long erroneous reads of the third generation sequencing technologies. *Sci Rep* **6**, 31900 (2016).

34. English AC*, et al.* Mind the gap: upgrading genomes with Pacific Biosciences RS long-read sequencing technology. *PLoS ONE* **7**, e47768 (2012).

35. Smit A, Hubley, R. (RepeatModeler Open-1.0.) (2008–2015).

36. Bao W, Kojima KK, Kohany O. Repbase Update, a database of repetitive elements in eukaryotic genomes. *Mobile DNA* **6**, 11 (2015).

37. Tarailo-Graovac M, Chen N. Using RepeatMasker to identify repetitive elements in genomic sequences. *Curr Protoc Bioinformatics* **Chapter 4**, Unit 4.10 (2009).

38. Stock F*, et al.* Distinctive growth and transcriptional changes of the diatom *Seminavis robusta* in response to quorum sensing related compounds. *Front Microbiol*, (2020).

39. Cirri E*, et al.* Associated bacteria affect sexual reproduction by altering gene expression and metabolic processes in a biofilm inhabiting diatom. *Front Microbiol* **10**, 1790 (2019).

40. Bilcke G*, et al.* Mating type specific transcriptomic response to sex inducing pheromone in the pennate diatom *Seminavis robusta*. Preprint at https://www.biorxiv.org/content/10.1101/2020.03.16.987719v1 (2020).

41. Moeys S*, et al.* A sex-inducing pheromone triggers cell cycle arrest and mate attraction in the diatom *Seminavis robust*a. *Sci Rep* **6**, 19252 (2016).

42. Nymark M*, et al.* An integrated analysis of molecular acclimation to high light in the marine diatom *Phaeodactylum tricornutum. PLoS ONE* **4**, e7743 (2009).

43. Bolger AM, Lohse M, Usadel B. Trimmomatic: a flexible trimmer for Illumina sequence data. *Bioinformatics* **30**, 2114-2120 (2014).

44. Arabidopsis Genome I. Analysis of the genome sequence of the flowering plant *Arabidopsis thaliana*. *Nature* **408**, 796-815 (2000).

45. Gobler CJ*, et al.* Niche of harmful alga *Aureococcus anophagefferens* revealed through ecogenomics. *Proc Natl Acad Sci U S A* **108**, 4352-4357 (2011).

46. Consortium CeS. Genome sequence of the nematode *C. elegans*: a platform for investigating biology. *Science* **282**, 2012-2018 (1998).

47. Merchant SS*, et al.* The Chlamydomonas genome reveals the evolution of key animal and plant functions. *Science* **318**, 245-250 (2007).

48. Janouskovec J*, et al.* Evolution of red algal plastid genomes: ancient architectures, introns, horizontal gene transfer, and taxonomic utility of plastid markers. *PLoS ONE* **8**, e59001 (2013).

49. Matsuzaki M*, et al.* Genome sequence of the ultrasmall unicellular red alga *Cyanidioschyzon merolae* 10D. *Nature* **428**, 653-657 (2004).

50. Adams MD*, et al.* The genome sequence of *Drosophila melanogaster*. *Science* **287**, 2185-2195 (2000).

51. Cock JM, *et al.* The Ectocarpus genome and the independent evolution of multicellularity in brown algae. *Nature* **465**, 617-621 (2010).

52. Read BA, *et al.* Pan genome of the phytoplankton *Emiliania underpins* its global distribution. *Nature* **499**, 209-213 (2013).

53. Tanaka T, *et al.* Oil accumulation by the oleaginous diatom *Fistulifera solaris* as revealed by the genome and transcriptome. *Plant Cell* **27**, 162-176 (2015).

54. Mock T, *et al.* Evolutionary genomics of the cold-adapted diatom *Fragilariopsis cylindrus*. *Nature* **541**, 536-540 (2017).

55. Schonknecht G, *et al.* Gene transfer from bacteria and archaea facilitated evolution of an extremophilic eukaryote. *Science* **339**, 1207-1210 (2013).

56. Venter JC, *et al.* The sequence of the human genome. *Science* **291**, 1304-1351 (2001).

57. Corteggiani Carpinelli E, *et al.* Chromosome scale genome assembly and transcriptome profiling of *Nannochloropsis gaditana* in nitrogen depletion. *Molecular Plant* **7**, 323-335 (2014).

58. Aury JM, *et al.* Global trends of whole-genome duplications revealed by the ciliate *Paramecium tetraurelia*. *Nature* **444**, 171-178 (2006).

59. Bowler C, *et al.* The *Phaeodactylum* genome reveals the evolutionary history of diatom genomes. *Nature* **456**, 239-244 (2008).

60. Rensing SA, *et al.* The *Physcomitrella* genome reveals evolutionary insights into the conquest of land by plants. *Science* **319**, 64-69 (2008).

61. Armbrust EV. Pseudo-nitzschia multiseries genome.) (2011).

62. Basu S, *et al.* Finding a partner in the ocean: molecular and evolutionary bases of the response to sexual cues in a planktonic diatom. *New Phytol* **215**, 140-156 (2017).

63. Goffeau A, *et al.* Life with 6000 genes. *Science* **274**, 546, 563-547 (1996).

64. Wood V, *et al.* The genome sequence of *Schizosaccharomyces pombe*. *Nature* **415**, 871-880 (2002).

65. Galachyants YP, *et al.* Sequencing of the complete genome of an araphid pennate diatom *Synedra acus* subsp. *radians* from Lake Baikal. *Dokl Biochem Biophys* **461**, 84-88 (2015).

66. Lommer M, *et al.* Genome and low-iron response of an oceanic diatom adapted to chronic iron limitation. *Genome Biology* **13**, R66 (2012).

67. Armbrust EV, *et al.* The genome of the diatom *Thalassiosira pseudonana*: ecology, evolution, and metabolism. *Science* **306**, 79-86 (2004).

68. Buchfink B, Xie C, Huson DH. Fast and sensitive protein alignment using DIAMOND. *Nature Methods* **12**, 59-60 (2015).

69. Enright AJ, Van Dongen S, Ouzounis CA. An efficient algorithm for large-scale detection of protein families. *Nucleic Acids Research* **30**, 1575-1584 (2002).

70. Price MN, Dehal PS, Arkin AP. FastTree 2--approximately maximum-likelihood trees for large alignments. *PLoS ONE* **5**, e9490 (2010).

71. Stamatakis A. RAxML version 8: a tool for phylogenetic analysis and post-analysis of large phylogenies. *Bioinformatics* **30**, 1312-1313 (2014).

72. Proost S, *et al.* i-ADHoRe 3.0--fast and sensitive detection of genomic homology in extremely large data sets. *Nucleic Acids Research* **40**, e11 (2012).

73. Guillard RRL. Culture of phytoplankton for feeding marine invertebrates. In: *Culture of Marine Invertebrate Animals* (eds Smith WL, Canley MH) (1975).

74. Wysoker AaT, Kathleen and Fennell, Tim. . Picard tools version 2.6.) (2016).

75. McKenna A, *et al.* The Genome Analysis Toolkit: a MapReduce framework for analyzing next-generation DNA sequencing data. *Genome Res* **20**, 1297-1303 (2010).

76. Li H, *et al.* The Sequence Alignment/Map format and SAMtools. *Bioinformatics* **25**, 2078-2079 (2009).

77. Li H. A statistical framework for SNP calling, mutation discovery, association mapping and population genetical parameter estimation from sequencing data. *Bioinformatics* **27**, 2987-2993 (2011).

78. Gurevich A, Saveliev V, Vyahhi N, Tesler G. QUAST: quality assessment tool for genome assemblies. *Bioinformatics* **29**, 1072-1075 (2013).

79. Wu TD, Watanabe CK. GMAP: a genomic mapping and alignment program for mRNA and EST sequences. *Bioinformatics* **21**, 1859-1875 (2005).

80. Van Bel M, Proost S, Van Neste C, Deforce D, Van de Peer Y, Vandepoele K. TRAPID: an efficient online tool for the functional and comparative analysis of *de novo* RNA-Seq transcriptomes. *Genome Biology* **14**, R134 (2013).

81. Van Bel M*, et al.* Dissecting plant genomes with the PLAZA comparative genomics platform. *Plant Physiology* **158**, 590-600 (2012).

82. Vandepoele K*, et al.* pico-PLAZA, a genome database of microbial photosynthetic eukaryotes. *Environmental Microbiology* **15**, 2147-2153 (2013).

83. Veeckman E, Ruttink T, Vandepoele K. Are We There Yet? Reliably estimating the completeness of plant genome sequences. *Plant Cell* **28**, 1759-1768 (2016).

84. Thamatrakoln K, Hildebrand M. Silicon uptake in diatoms revisited: a model for saturable and nonsaturable uptake kinetics and the role of silicon transporters. *Plant Physiology* **146**, 1397-1407 (2008).

85. Harada H, Nakajima K, Sakaue K, Matsuda Y. CO2 sensing at ocean surface mediated by cAMP in a marine diatom. *Plant Physiology* **142**, 1318-1328 (2006).

86. Port JA, Parker MS, Kodner RB, Wallace JC, Armbrust EV, Faustman EM. Identification of G protein-coupled receptor signaling pathway proteins in marine diatoms using comparative genomics. *BMC Genomics* **14**, 503 (2013).

87. Johansson ON*, et al.* Phenomics reveals a novel putative chloroplast fatty acid transporter in the marine diatom *Skeletonema marinoi* involved in temperature acclimation. *Sci Rep* **9**, 15143 (2019).

88. Gibbs GM, Roelants K, O'Bryan MK. The CAP superfamily: cysteine-rich secretory proteins, antigen 5, and pathogenesis-related 1 proteins--roles in reproduction, cancer, and immune defense. *Endocr Rev* **29**, 865-897 (2008).

89. Helliwell KE, *et al.* Alternative mechanisms for fast Na(+)/Ca(2+) signaling in eukaryotes via a novel class of single-domain voltage-gated channels. *Curr Biol* **29**, 1503-1511.e1506 (2019).

90. Fortunato AE, *et al.* Diatom phytochromes reveal the existence of far-red-light-based sensing in the ocean. *Plant Cell* **28**, 616-628 (2016).

91. Gillard J, *et al.* Metabolomics enables the structure elucidation of a diatom sex pheromone. *Angew Chem Int Ed Engl* **52**, 854-857 (2013).

92. Stogios PJ, Downs GS, Jauhal JJ, Nandra SK, Prive GG. Sequence and structural analysis of BTB domain proteins. *Genome Biology* **6**, R82 (2005).

93. Ikawa M, Inoue N, Benham AM, Okabe M. Fertilization: a sperm's journey to and interaction with the oocyte. *J Clin Invest* **120**, 984-994 (2010).

94. Vinagre C, *et al.* Ecological traps in shallow coastal waters-potential effect of heat-waves in tropical and temperate organisms. *PLoS ONE* **13**, e0192700 (2018).

95. De Bortoli S, Teardo E, Szabo I, Morosinotto T, Alboresi A. Evolutionary insight into the ionotropic glutamate receptor superfamily of photosynthetic organisms. *Biophys Chem* **218**, 14-26 (2016).

96. Jian-Wei Zheng D-WL, Yang Lu, Jian Chen, Jin-Jin Liang, Lin Zhang, Wei-Dong Yang, Jie-Sheng Liu, Song-Hui Lu, Hong-Ye Li. Molecular exploration of algal interaction between the diatom *Phaeodactylum tricornutum* and the dinoflagellate *Alexandrium tamarense*. *Algal Research* **17**, 132-141 (2016).

97. Buhmann MT, Schulze B, Forderer A, Schleheck D, Kroth PG. Bacteria may induce the secretion of mucin-like proteins by the diatom *Phaeodactylum tricornutum*. *J Phycol* **52**, 463-474 (2016).

98. Stock F, *et al*. Distinctive growth and transcriptional changes of the diatom *Seminavis robusta* in response to quorum sensing related compounds. *Frontiers in Microbiology* **11**, 1240 (2020)

99. Campa F, Randazzo PA. Arf GTPase-activating proteins and their potential role in cell migration and invasion. *Cell Adh Migr* **2**, 258-262 (2008).

100. Danecek P, *et al.* The variant call format and VCFtools. *Bioinformatics* **27**, 2156-2158 (2011).

101. Cingolani P, *et al.* A program for annotating and predicting the effects of single nucleotide polymorphisms, SnpEff: SNPs in the genome of *Drosophila melanogaste*r strain w1118; iso-2; iso-3. *Fly (Austin)* **6**, 80-92 (2012).

102. De Decker S, *et al.* Incomplete reproductive isolation between genetically distinct sympatric clades of the pennate model diatom *Seminavis robusta*. *Protist* **169**, 569-583 (2018).

103. Aumeier C, Polinski E, Menzel D. Actin, actin-related proteins and profilin in diatoms: a comparative genomic analysis. *Marine genomics* **23**, 133-142 (2015).

104. Hammesfahr B, Kollmar M. Evolution of the eukaryotic dynactin complex, the activator of cytoplasmic dynein. *BMC Evol Biol* **12**, 95 (2012).

105. Pucciarelli S, Ballarini P, Miceli C. Cold-adapted microtubules: characterization of tubulin posttranslational modifications in the Antarctic ciliate *Euplotes focardii*. *Cell Motil Cytoskeleton* **38**, 329-340 (1997).

106. Pei J, Grishin NV. Type II CAAX prenyl endopeptidases belong to a novel superfamily of putative membrane-bound metalloproteases. *Trends Biochem Sci* **26**, 275-277 (2001).

107. Villalobo A, Gonzalez-Munoz M, Berchtold MW. Proteins with calmodulin-like domains: structures and functional roles. *Cell Mol Life Sci* **76**, 2299-2328 (2019).

108. McLachlan DH, Underwood GJ, Taylor AR, Brownlee C. Calcium release from intracellular stores is necessary for the photophobic response in the benthic diatom *Navicula perminuta* (bacillariophyceae). *J Phycol* **48**, 675-681 (2012).

109. Glading A, Lauffenburger DA, Wells A. Cutting to the chase: calpain proteases in cell motility. *Trends Cell Biol* **12**, 46-54 (2002).

110. Franco SJ, Huttenlocher A. Regulating cell migration: calpains make the cut. *J Cell Sci* **118**, 3829-3838 (2005).

111. Frigeri LG, Radabaugh TR, Haynes PA, Hildebrand M. Identification of proteins from a cell wall fraction of the diatom *Thalassiosira pseudonana*: insights into silica structure formation. *Mol Cell Proteomics* **5**, 182-193 (2006).

112. Hutagalung AH, Novick PJ. Role of Rab GTPases in membrane traffic and cell physiology. *Physiol Rev* **91**, 119-149 (2011).

113. Smith SR, *et al.* Evolution and regulation of nitrogen flux through compartmentalized metabolic networks in a marine diatom. *Nat Commun* **10**, 4552 (2019).
