# Supplementary material for: The Seminavis robusta genome provides insights into the evolutionary adaptations of benthic diatoms
Source: Nat Commun. 2020 Jul 3;11:3320. doi: 10.1038/s41467-020-17191-8 (PMC7335047; doi:10.1038/s41467-020-17191-8)
Supplement: Supplementary file 3 — Reporting Summary [file 41467_2020_17191_MOESM3_ESM.pdf]

nature research

Corresponding author(s): Klaas Vandepoele

Last updated by author(s): June 4, 2020

Reporting Summary

Nature Research wishes to improve the reproducibility of the work that we publish. This form provides structure for consistency and transparency in reporting. For further information on Nature Research policies, see Authors & Referees and the Editorial Policy Checklist .

Statistics

For all statistical analyses, confirm that the following items are present in the figure legend, table legend, main text, or Methods section.

n/a	Confirmed	
☐	☒	The exact sample size (*n*) for each experimental group/condition, given as a discrete number and unit of measurement
☐	☒	A statement on whether measurements were taken from distinct samples or whether the same sample was measured repeatedly
☐	☒	The statistical test(s) used AND whether they are one- or two-sided<br>*Only common tests should be described solely by name; describe more complex techniques in the Methods section.*
☒	☐	A description of all covariates tested
☐	☒	A description of any assumptions or corrections, such as tests of normality and adjustment for multiple comparisons
☐	☒	A full description of the statistical parameters including central tendency (e.g. means) or other basic estimates (e.g. regression coefficient) AND variation (e.g. standard deviation) or associated estimates of uncertainty (e.g. confidence intervals)
☐	☒	For null hypothesis testing, the test statistic (e.g. *F*, *t*, *r*) with confidence intervals, effect sizes, degrees of freedom and *P* value noted<br>*Give P values as exact values whenever suitable.*
☒	☐	For Bayesian analysis, information on the choice of priors and Markov chain Monte Carlo settings
☒	☐	For hierarchical and complex designs, identification of the appropriate level for tests and full reporting of outcomes
☐	☒	Estimates of effect sizes (e.g. Cohen's *d*, Pearson's *r*), indicating how they were calculated

Our web collection on statistics for biologists contains articles on many of the points above.

Software and code

Policy information about availability of computer code

Data collection

All S. robusta strains used in this study are available in the BCCM/DCG diatom culture collection (http://bccm.belspo.be/about-us/bccm-dcg). Specifically, the reference strain for DNA sequencing "D6" (DCG 0498) and strain "85A" (DCG 0105) used for most RNA-seq experiments are progeny laboratory cultures retrieved from BCCM/DCG. PONTON34 (DCG 0460) and PONTON36 (DCG 0462) used in sexual reproduction RNA-seq experiments are natural strains originally collected at the Veerse Meer in the Netherlands (51°32'32.45"N 3°47'54.76"E) retrieved from BCCM/DCG.

Data analysis

Tools used in this article (further details about command lines are found in Methods and/or Supplementary Information):
- Jellyfish v2.2.6 (DOI: 10.1093/bioinformatics/btr011)
- GenomeScope v1 (DOI: doi: 10.1093/bioinformatics/btx153)
- BBDuk v34.56 (http://jgi.doe.gov/data-and-tools/bbtools/bb-tools-user-guide/installation-guide/)
- PEAR v0.9.6 (DOI: 10.1093/bioinformatics/btt593)
- LoRDEC v0.5 (DOI: 10.1093/bioinformatics/btu538)
- Platanus v1.2.4 (DOI: 10.1101/gr.170720.113)
- PBJelly v15.8.24 (DOI: 10.1371/journal.pone.0047768)
- BLASR v5.3.2 (DOI: 10.1186/1471-2105-13-238)
- SMRTAnalysis v2.2.0 (DOI: 10.1038/nmeth.2474)
- Canu v1.4 (DOI: 10.1101/gr.215087.116)
- FALCON-integrate v5 (DOI: 10.1038/nmeth.4035)
- DBG2OLC v2015-05-19 (DOI: 10.1038/srep31900)
- QUAST v4.4 (DOI: 10.1093/bioinformatics/btt086)
- GMAP v2016-04-04 (DOI: 10.1093/bioinformatics/bti310)
- TRAPID v1 (DOI: 10.1186/gb-2013-14-12-r134)
- BLASTn v2.3.0 (DOI: 10.1016/S0022-2836(05)80360-2)
- RepeatMasker v4.0.5 (DOI: 10.1002/0471250953.bi0410s25)

- RepeatModeler v1.0.8 (http://www.repeatmasker.org)
- BRAKER v1 (DOI: 10.1093/bioinformatics/btv661)
- STAR v2.5.2 (DOI: 10.1093/bioinformatics/bts635)
- Genome-guided Trinity De novo Transcriptome v2.6.6 (https://github.com/trinityrnaseq/trinityrnaseq/wiki/Genome-Guided-Trinity-Transcriptome-Assembly)
- PASA v2.3.3 (DOI: 10.1093/nar/gkg770)
- Tera-BLASTX v7.6.1 (http://www.timelogic.com/catalog/757/tera-blast)
- InterProScan v5.31 (DOI: 10.1093/bioinformatics/btu031)
- EggNOG-mapper v1 (DOI: 10.1093/molbev/msx148)
- AnnoMine v1 (DOI: 10.1111/1462-2920.12174)
- Infernal v1.1.2 (DOI: 10.1093/bioinformatics/btp157)
- tRNAscan-SE v1.31 (DOI: 10.1093/nar/25.5.955)
- GeSeq v1 (DOI: 10.1093/nar/gkx391)
- OGDraw v1.3.1 (DOI: 10.1093/nar/gkz238)
- Trimmomatic v0.36 (DOI: 10.1093/bioinformatics/btu170)
- Salmon v0.9.1 (DOI: 10.1038/nmeth.4197)
- tximport package v1.8.0 ()
- EdgeR v3.10 (DOI: 10.1093/bioinformatics/btp616)
- FastQC v0.11.4 (DOI: 10.12688/f1000research.15931.2)
- BWA-MEM v0.7.5a (https://github.com/lh3/bwa)
- Samtools v1.6 (DOI: 10.1093/bioinformatics/btp352)
- VelvetOptimizer (http://bioinformatics.net.au/software.velvetoptimiser.shtml)
- Bedtools v2.27 (DOI: 10.1093/bioinformatics/btq033)
- CD-HIT v4.8.1(DOI: 10.1093/bioinformatics/bts565)
- SGSGeneLoss v0.1(http://www.appliedbioinformatics.com.au/index.php/SGSGeneLoss)
- Picard v1.94 (https://broadinstitute.github.io/picard/)
- GATK v3.7-0 (DOI: 10.1101/gr.107524.110)
- VCFtools v0.1.16 (DOI: 10.1093/bioinformatics/btr330)
- Bcftools v1.3 (DOI: 10.1093/bioinformatics/btr509)
- snpEff v4.3t (DOI: 10.4161/fly.19695)
- Diamond v0.9.18 (DOI: 10.1038/nmeth.3176)
- MAFFT v7.187 (DOI: 10.1093/molbev/mst010)
- trimal v1.4.1 (DOI: 10.1093/bioinformatics/btp348)
- IQ-TREE v1.7 (DOI: 10.1093/molbev/msu300)
- FigTree v1.4.4 (https://github.com/rambaut/figtree/releases)
- TribeMCL v10-201 (DOI: 10.1093/nar/30.7.1575)
- FastTree v2.1.7 (DOI: 10.1371/journal.pone.0009490)
- RaxML v8.2.9 (DOI: 10.1093/bioinformatics/btu033)
- i-ADHoRe v3.0 (DOI: 10.1093/nar/gkr955)

For manuscripts utilizing custom algorithms or software that are central to the research but not yet described in published literature, software must be made available to editors/reviewers. We strongly encourage code deposition in a community repository (e.g. GitHub). See the Nature Research guidelines for submitting code & software for further information.

Data

Policy information about availability of data

All manuscripts must include a data availability statement. This statement should provide the following information, where applicable:

- Accession codes, unique identifiers, or web links for publicly available datasets
- A list of figures that have associated raw data
- A description of any restrictions on data availability

Data supporting the findings of this work are available within the paper and its Supplementary Information files. The genome assembly and gene model annotation can be downloaded from the ORCAE platform [https://bioinformatics.psb.ugent.be/orcae/overview/Semro]. DNA sequencing data that support the findings of this study have been deposited in the European Nucleotide Archive with the accession code of PRJEB36614 [https://www.ebi.ac.uk/ena/browser/view/PRJEB36614] while the fastq files representing the raw RNA sequencing data have been deposited in the EBI Array Express under accession number E-MTAB-8685 [https://www.ebi.ac.uk/arrayexpress/experiments/E-MTAB-8685/]. Source for comparative genomics analysis of this study is accessible in the PLAZA Diatoms 1.0 [https://bioinformatics.psb.ugent.be/plaza/versions/plaza_diatoms_01/], including additionally on each gene family page a direct link to the TPM and the differential gene expression matrix of all S. robusta genes. The Supplementary Data 1–7 underlying Figures 2B, 3A-D, 4A-D and 5A-C, as well as Supplementary Figures 10, 13, 15, 16, 17, 20 and 22 are provided as a Source Data file.

Field-specific reporting

Please select the one below that is the best fit for your research. If you are not sure, read the appropriate sections before making your selection.

☒ Life sciences ☐ Behavioural & social sciences ☐ Ecological, evolutionary & environmental sciences

For a reference copy of the document with all sections, see nature.com/documents/nr-reporting-summary-flat.pdf

Life sciences study design

All studies must disclose on these points even when the disclosure is negative.

Sample size	No sample size estimation was required in our work.
Data exclusions	No data were excluded from the analysis.
Replication	For the new RNA-Seq experiments generated in this study, three biological replicates were generated to ensure robustness and valid biological interpretation of the differential gene expression analysis. All attempts at replication were successful. A detailed overview of experimental conditions is given in Supplementary Table 7.
Randomization	No randomization was performed because the study-design did not require such procedure.
Blinding	No blinding was applied to this study as this technique does not apply to the work reported here.

Reporting for specific materials, systems and methods

We require information from authors about some types of materials, experimental systems and methods used in many studies. Here, indicate whether each material, system or method listed is relevant to your study. If you are not sure if a list item applies to your research, read the appropriate section before selecting a response.

Materials & experimental systems

n/a	Involved in the study	
☒	☐	Antibodies
☒	☐	Eukaryotic cell lines
☒	☐	Palaeontology
☒	☐	Animals and other organisms
☒	☐	Human research participants
☒	☐	Clinical data

Methods

n/a	Involved in the study	
☒	☐	ChIP-seq
☒	☐	Flow cytometry
☒	☐	MRI-based neuroimaging
