# Supplementary material for: The Seminavis robusta genome provides insights into the evolutionary adaptations of benthic diatoms
Source: Nat Commun. 2020 Jul 3;11:3320. doi: 10.1038/s41467-020-17191-8 (PMC7335047; doi:10.1038/s41467-020-17191-8)
Supplement: Supplementary file 2 — Peer Review [file 41467_2020_17191_MOESM2_ESM.pdf]

Reviewers' comments:

Reviewer #1 (Remarks to the Author):

The authors report the high quality genome data with numerous analyses of the benthic diatom Seminavis robusta. The addition of this benthic diatom to previously reported diatoms genomes provides the authors with an interesting framework to study the evolution of the different diatome types. The authors report a very thorough analysis presented in a clear and intelligible way.
The authors notably show that the gene repository of the benthic diatom was enriched by tandem duplications from genes having functions related to benthic environments, such as biofilm, interactions with bacteria.
Overall, I do not see any major issue with the analysis; the manuscript was as I said thorough and clear. The discussion was balanced and convincing. I also do not see any further analysis that could enrich the manuscript as it covers really well the objectives set by the authors as it is. The only thing one I might recommend is to move some of the analysis to the supplementary material, particularly I was not entirely convinced by the resequencing analysis, so that it might make the manuscript a more focused on a particular and interesting topic(s).

Reviewer #2 (Remarks to the Author):

This is an interesting manuscript reporting the genome sequence of a benthic diatom, Seminavis robusta, and other genomic resources, allowing evolutionary and functional insights on how diatoms adapt to and structure benthic habitats. Besides a new genome sequence, this paper also presents useful new resources, like a huge transcriptomic effort with many conditions of interest for understanding Seminavis ecology, and re-sequencing of 48 strains with potential to unravel selective pressures. The analyses on gene family expansion, and particularly the role of tandem gene duplications, are particularly convincing in general. There are however many other aspects that should be improved, including the quality of the genome assembly, as well as analyses on variable gene composition, selection, and evolution of the gene repertoire according to lifestyles versus phylogeny.

Main points
The quality of the genome assembly appears relatively low in terms of contiguity compared to current standards. The use of a high coverage of long PacBio reads supports the expectation of a more continuous sequence. Although this does not impact strongly the main conclusions of the paper, the usefulness of the resource may be lessened. Regarding the annotation, it is unclear how the repeat detection has affected gene prediction, particularly in light of the conclusions that the S.r. genome has large family expansions (see page 3). Can the authors provide an assessment on how this was tested?
Page 6, the authors noted that “We observed that families with an increasing number of genes tended to display higher expression divergence than smaller families (Figure 3A)”. Looking at Fig3A does not lead so obviously to the conclusions, it seems that gene families with >4 members have no clear trend. Subsequently, it is written that “families showing expression divergence are significantly enriched in eukaryote and diatom age classes. Reversely, families showing expression conservation are significantly enriched in the species-specific age class”. This would not be coherent with the idea that gene duplications have played a role in adaptation in this species. Can the authors explain better how these observations can be reconciled?
From page 10, a long paragraph based on resequencing of multiple strains develops ideas on how the Seminavis pan-genome is shaped. This is based mostly on short-read data and assemblies. It is well known that these methods lead to incomplete genomes, with the consequence that the variable gene complement is inflated. As seen in Figure 4A, the core genome is diminishing to an unrealistic point because of this bias. A correct analysis of pan-genome can only be built on high-quality sequences, so I suggest that the authors remove most of this and concentrate on the gene novelties appearing in the re-sequenced genomes, as those might have been expected in the better quality reference. In addition

it is written page 11 “many dispensable genes lack functional annotation, the remaining genes are enriched in DNA metabolism, zinc fingers, transposable element-derived proteins, and telomeric proteins”. These annotations suggest potential retentions of repetitive sequences in the gene set, which have to be checked.
Page 12, an analysis of the purifying and positive selections led to the conclusion that dispensable genes are less constrained. This is also hypothesized for genes of unknown functions. In both cases, the set is enriched for poorly conserved genes, which may be difficult to annotate, and potentially could create cases of poor alignments interpreted as cases of positive selections. The authors should show more convincingly that this is not the case, testing for biases potentially introduced in this analysis.
From page 13 onwards, an interesting conservation analysis is made using 88 transcriptomes of very diverse diatoms from the MMETSP. The authors found interesting cases of apparent correlation between gene presence and some traits, and provide explanations like “benthic signatures” or raphid-specific traits. This would be reinforced by a clear comparison on how the traits and gene amplifications are present in the diatom phylogeny. This would be more insightful than some of the present conclusions on rhodopsins and ROS which are expected, and not experimentally shown to be linked to the hypotheses.

Minor point
Page 5, the diatoms with larger genomes are shown to have more gene families than those with smaller genomes. Is this conclusion robust with the way the heterozygosity was treated in the other studies?

Reviewer #3 (Remarks to the Author):

The manuscript by Osuna-Cruz et al. ostensibly presents the genome of the benthic diatom Seminavis robusta. However, the manuscript goes well beyond a genome “announcement” and includes results from re-sequencing of 48 S. robusta strains from three clades to identify core genes and strain-specific genes, analysis of dozens of transcriptomes to enhance gene-model predictions and identify gene families induced under specific or multiple conditions, and incorporates comparative analyses (including the open Diatom Plaza) to identify genes essential to life in a benthic environment. This is a stunning body of work that raises the bar very high for other genome papers and will undoubtedly be emulated by others. Therefore, I provide here some detailed suggestions to ensure that others can reproduce the results and analyses. I also suggest that the authors discuss their results in a bit more detail and provide more comparisons to what is known from other organisms. The discussion of the purifying selection is a good example of comparative discussion that should be enhanced in some other sections.

1) Figure 2. Panel A. The authors should not assume that the reader will know what an “upset plot” is and should provide more details. For example, what are the black vs gray circles in the rows and what do the vertical lines connote?
2) Figure 3. Panel A. Axis label descriptions in legend are reversed. Panel D. This panel uses a lot of symbols/colors. Please make sure all are described. For example, what do the colored boxes indicate? The color legend in the center of the figure seems to relate to panels B, C and D, but this is hard to understand.
3) Figure 5. The experimental design was not entirely clear. It was not immediately clear to me that the MMETSP data was used only to define the different categories of genes that would be analyzed in the Seminavis transcriptomes and that the up/down arrows were derived from these latter analyses.
4) The authors provide citations for all the software used in their analyses. Please also include all settings used to allow others to reproduce the results.
5) The authors generate additional transcriptomes for this manuscript. They need to provide additional details about the growth and harvest conditions.

6) The authors should provide details about the flow cytometry methods used to estimate genome size and possible explanations for the discrepancy in size estimates.
7) The authors should provide more details on the manual checking used to remove only the 4 scaffolds out of 33 scaffolds that were designated as potential bacterial contamination.
8) Figure S3. This figure/legend needs more detail to be interpretable. What are the different lanes for each labeled contig? Although expected product sizes are provided in Table S8, it would be helpful to include the expected product size in this figure.
9) Detection of reference-specific genes deserves more discussion. What is the likelihood that these genes are truly strain-specific? What sequencing depth of the re-sequenced strains would be required to address this question?
10) The sentence on page 10 beginning with "The six times higher fraction of dispensable genes…" requires a reference citation. Also, it is not clear whether the sentence on page 15 beginning with "Expression analysis of the GPCR rhodpson-like protein ortholog…" is part of this study or is a reference to other work. Please clarify.
11) Please explain how reference strain chosen and add specificity to DNA isolation techniques. Clarify if this strain was used for transcriptome studies and if not used (as it appears), please explain why.
12) Please note that diatoms have metacaspases, not caspases (page 11). Please correct.
13) Page 4. The authors state "Newly generated expression data combined with existing data…" Please provide references for existing data.

Response letter NCOMMS-20-04487A - The *Seminavis robusta* genome provides insights into the evolutionary adaptations of benthic diatoms

Reviewer #1 (Remarks to the Author):

The authors report the high quality genome data with numerous analyses of the benthic diatom Seminavis robusta. The addition of this benthic diatom to previously reported diatoms genomes provides the authors with an interesting framework to study the evolution of the different diatom types. The authors report a very thorough analysis presented in a clear and intelligible way. The authors notably show that the gene repository of the benthic diatom was enriched by tandem duplications from genes having functions related to benthic environments, such as biofilm, interactions with bacteria. Overall, I do not see any major issue with the analysis; the manuscript was as I said thorough and clear. The discussion was balanced and convincing. I also do not see any further analysis that could enrich the manuscript as it covers really well the objectives set by the authors as it is. The only thing one I might recommend is to move some of the analysis to the supplementary material, particularly I was not entirely convinced by the resequencing analysis, so that it might make the manuscript a more focused on a particular and interesting topic(s).

We thank the reviewer for the positive comments on our manuscript. As requested, we have reduced the section describing the resequencing analysis (see also Reviewer 2 - comment 4).

Reviewer #2 (Remarks to the Author):

This is an interesting manuscript reporting the genome sequence of a benthic diatom, Seminavis robusta, and other genomic resources, allowing evolutionary and functional insights on how diatoms adapt to and structure benthic habitats. Besides a new genome sequence, this paper also presents useful new resources, like a huge transcriptomic effort with many conditions of interest for understanding Seminavis ecology, and re-sequencing of 48 strains with potential to unravel selective pressures. The analyses on gene family expansion, and particularly the role of tandem gene duplications, are particularly convincing in general. There are however many other aspects that should be improved, including the quality of the genome assembly, as well as analyses on variable gene composition, selection, and evolution of the gene repertoire according to lifestyles versus phylogeny.

We thank the reviewer for the positive comments on our manuscript and his/her appreciation for the comparative gene family analyses we performed. Below we report different arguments explaining why the analyses questioned by the reviewer are not suffering from low quality.

Main points

1) The quality of the genome assembly appears relatively low in terms of contiguity compared to current standards. The use of a high coverage of long PacBio reads supports the expectation of a more continuous sequence. Although this does not impact strongly the main conclusions of the paper, the usefulness of the resource may be lessened.

In contrast to what intuitively could be expected, generating chromosome-level assemblies for diatoms is not as easy as the current standards applicable for plant, animal and fungal genomes.

A. Diatoms have silicified cell walls, which means that the high molecular weight (HMW) DNA extraction is not trivial. Harsh conditions are needed to break the wall, which also affects the integrity of the chromosomal DNA. Our current assembly is based on a hybrid dataset combining 18M Illumina paired-end reads as well as >1M PacBio long reads. For the latter, we tested different HMW DNA extraction protocols, applied Blue Pippin size selection to enrich for long fragments, and used two different service providers. In all scenarios the obtained average read length was around 5kb (*Supplementary Table S1*), which is lower compared to standard PacBio read lengths applied on other non-diatom species. A comparison with the *Fragilariopsis cylindrus* genome (Mock et al., Nature, 2017) where the average PacBio read length was 2.4kb reveals our long reads are of excellent quality. In addition, checking all the diatom genomes published during the last 5 years revealed none have reported long read data of superior quality (Tanaka et al., 2015, Traller et al., 2016, Basu et al., 2017, Rastogi et al., 2018).

B. As depicted in the *Figure 1* below (plotting genome size against the number of scaffolds; a revised version of *Figure 1B* in the manuscript) and focusing on diatoms with complex large genomes (>90Mb, containing a substantial fraction of repeats), the contiguity of our assembly is perfectly in line with the current state-of-the-art and is one of the best diatom assemblies produced so far. Although we agree that having a fragmented genome might impede a number of specific bio-informatics analysis, the obtained "Completeness score" reveals that our gene space is more complete compared to other diatoms with large genomes, and thus that our data represents a valuable genomic resource.

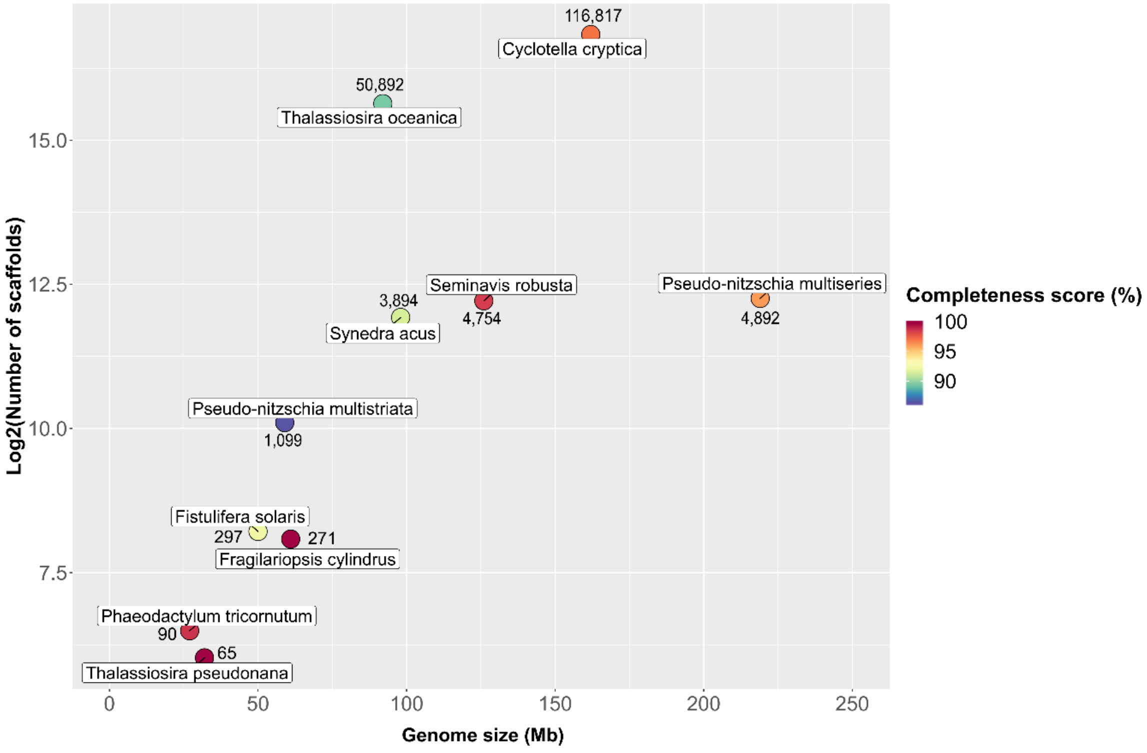

Figure 1. Scatter plot showing genome assembly contiguity (number of scaffolds) in function of genome size. Genome assemblies are colored according to the gene family completeness score in a rainbow scale from blue to red. Black numbers indicate number of scaffolds.

C. For the last 5 months, we have been in contact with Dovetail Genomics (US), a service provider with extensive expertise in Hi-C scaffolding of complex genomes. After many discussions, it turned out that they so far never successfully applied their technology on diatoms (see https://dovetailgenomics.com/dovetail-tree-of-life/), underscoring that building chromosome-level assemblies for diatom species is much more challenging than for plants/animals.

Taken together, we argue that the diatom assembly presented in this paper meets the current standard for genome contiguity, while its gene repertoire completeness is among the best of available diatom genome assemblies.

References:

Mock, T., et al. (2017). Evolutionary genomics of the cold-adapted diatom Fragilariopsis cylindrus. Nature 541, 536-540.

Basu, S., Patil, S., Mapleson, D., Russo, M.T., Vitale, L., Fevola, C., Maumus, F., Casotti, R., Mock, T., Caccamo, M., Montresor, M., Sanges, R., and Ferrante, M.I. (2017). Finding a partner in the ocean: molecular and evolutionary bases of the response to sexual cues in a planktonic diatom. New Phytol 215, 140-156.

Rastogi, A., Maheswari, U., Dorrell, R.G., Vieira, F.R.J., Maumus, F., Kustka, A., McCarthy, J., Allen, A.E., Kersey, P., Bowler, C., and Tirichine, L. (2018). Integrative analysis of large scale transcriptome data draws a comprehensive landscape of Phaeodactylum tricornutum genome and evolutionary origin of diatoms. Sci Rep 8, 4834.

Tanaka, T., Maeda, Y., Veluchamy, A., Tanaka, M., Abida, H., Marechal, E., Bowler, C., Muto, M., Sunaga, Y., Tanaka, M., Yoshino, T., Taniguchi, T., Fukuda, Y., Nemoto, M., Matsumoto, M., Wong, P.S., Aburatani, S., and Fujibuchi, W. (2015). Oil accumulation by the oleaginous diatom Fistulifera solaris as revealed by the genome and transcriptome. Plant Cell 27, 162-176.

Traller, J.C., Cokus, S.J., Lopez, D.A., Gaidarenko, O., Smith, S.R., McCrow, J.P., Gallaher, S.D., Podell, S., Thompson, M., Cook, O., Morselli, M., Jaroszewicz, A., Allen, E.E., Allen, A.E., Merchant, S.S., Pellegrini, M., and Hildebrand, M. (2016). Genome and methylome of the oleaginous diatom Cyclotella cryptica reveal genetic flexibility toward a high lipid phenotype. Biotechnol Biofuels 9, 258.

2) Regarding the annotation, it is unclear how the repeat detection has affected gene prediction, particularly in light of the conclusions that the S.r. genome has large family expansions (see page 3). Can the authors provide an assessment on how this was tested?

As outlined in *Supplementary Table* S3, special care was taken when performing repeat analysis not to mask protein-coding genes, for example organized in tandem duplications, which might have repeat-like features. To do so, the species-specific repeat library built using RepeatModeler was screened for genuine protein-coding genes and these were removed from the repeat library (63/429 *de novo* repeats were discarded). We revised the footnotes in *Supplementary Table* S3.

“RepeatModeler v1.0.8 [31] was used to create a species-specific repeat library (-engine ncbi). This species-specific repeat library was inspected to identify sequences that correspond to genuine protein-coding genes (Tera-BLASTX v7.6.1 [3], e-value < 1e-05) and which were removed from the repeat library **(63/429 *de novo* repeats were discarded)**.”

To further assess if our analysis might suffer from overmasking repeats, we performed a control experiment where we mapped 10 randomly selected RNA-Seq samples to the unmasked *S. robusta* genome and quantified if annotated repeat regions correspond to expressed genuine protein-coding genes. None of the ~559 million evaluated reads mapped to the annotated repeat regions, showing we are not suffering from overmasking our genome.

Finally, we also assessed the potential undermasking of our genome by identifying the fraction of protein-coding genes that are annotated with functional descriptions containing “transposon”, “transposable”, “transposase”, “reverse transcriptase” or “integrase". In total, 260 protein-coding genes (0.7% of all predicted protein-coding genes) were annotated with these terms, which belong to 54 different gene families of which only 12 are included in our list of 594 expanded gene families.

In conclusion, all additional analyses confirm our repeat detection does not strongly affect our gene prediction and/or gene family expansion analysis.

3) Page 6, the authors noted that “We observed that families with an increasing number of genes tended to display higher expression divergence than smaller families (Figure 3A)”. Looking at Fig3A does not lead so obviously to the conclusions, it seems that gene families with >4 members have no clear trend. Subsequently, it is written that “families showing expression divergence are significantly enriched in eukaryote and diatom age classes. Reversely, families showing expression conservation are significantly enriched in the species-specific age class”. This would not be coherent with the idea that gene duplications have played a role in adaptation in this species. Can the authors explain better how these observations can be reconciled?

We thank the reviewer for spotting this apparent contradiction. The reported results in the manuscript are correct and are based on statistically significant patterns. In Figure 3A we showed that for gene families with more than five gene copies (with the exception of families with 9 gene copies) the median expression divergence is significantly higher than the median of all nodes (see legend). Alongside, as reported in *Supplementary Figure S11*, we observed a significant enrichment of older age categories for families showing expression divergence. However, as also shown in this figure, there are 86 diatom-specific and 66 species-specific gene families showing strong expression divergence. Consequently, it is wrong to assume that exclusively old gene families show expression divergence, potentially contributing to adaptive evolution. Furthermore, our results also reveal that old gene families can play an adaptive role through recent gene family expansions. To indicate the fact that we also observed younger gene families showing expression divergence, we revised the text.

“These results indicate that **globally** gene duplication is associated with expression divergence and that expression divergence increases with the age of gene duplicates. **However, 152 cases of diatom-specific or species-specific families showing strong expression divergence were found (*Supplementary Figure S11, Supplementary Table S8*), revealing that rapid expression evolution may also occurs for specific gene functions encoded by young gene families.**”

4) From page 10, a long paragraph based on resequencing of multiple strains develops ideas on how the Seminavis pan-genome is shaped. This is based mostly on short-read data and assemblies. It is well known that these methods lead to incomplete genomes, with the consequence that the variable gene complement is inflated. As seen in Figure 4A, the core genome is diminishing to an unrealistic point because of this bias. A correct analysis of pan-genome can only be built on high-quality sequences, so I suggest that the authors remove most of this and concentrate on the gene novelties appearing in the re-sequenced genomes, as those might have been expected in the better quality reference.

We understand this concern. We would like to clarify the applied methodology and show new results from some extra control experiments.

A. While it is true that low coverage data can lead to incomplete genomes, the strategy applied in this study to detect presence/absence variation of the reference genes in the re-sequencing strains is based only on read mapping, using the same strategy used in other pan genome studies (Golicz, A.A. et al., 2016, Montenegro J. D. et al., 2017, Gao L. et al., 2019). In particular, and as explained in the main methods, we executed the SGSGeneLoss package (Golicz, A.A. et al., 2015) and define a gene to be absent when the horizontal coverage across all its exons is < 5%. Hence, this approach is very relaxed and therefore we believe that our presence/absence variation results are not suffering from using low coverage data. To demonstrate that SGSGeneLoss can deal well with both high and low coverage data, we have performed a control experiment using the resequencing data of 10 *Phaeodactylum tricornutum* strains (Rastogi, A., et al., 2019) and the *S. robusta* resequencing data from 48 strains presented in this study. While the original resequencing data of *P. tricornutum* has a 72.9x average coverage, the resequencing data of *Seminavis robusta* has only a 8.26x average coverage. A random subsampling procedure was applied to reduce the coverage in both dataset and subsequently, SGSGeneLoss was executed to assess the variation on the number of core genes.

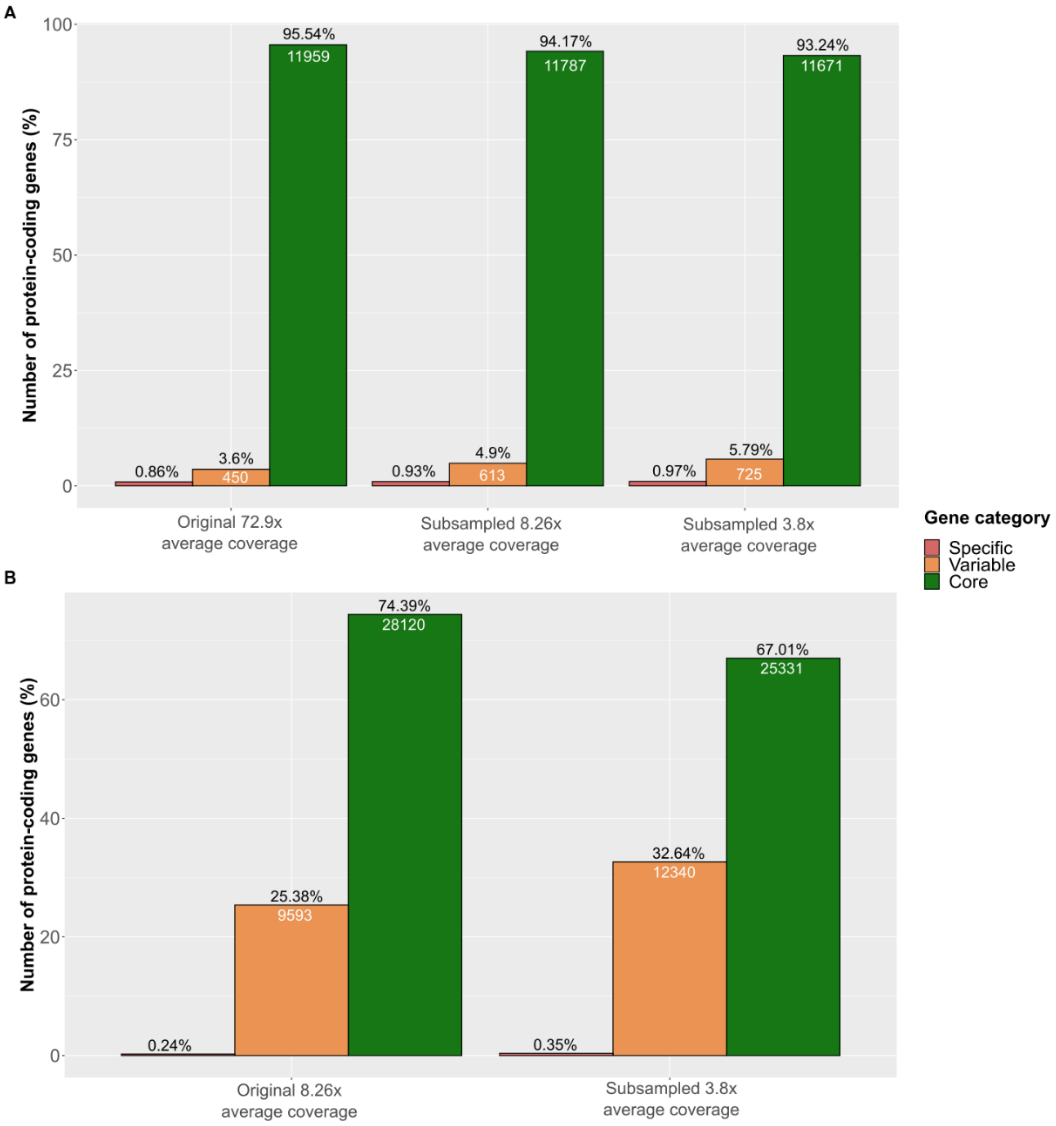

Figure 2. Fraction of dispensable (variable + specific) and core genes in *Phaeodactylum tricornutum* (A) and *Seminavis robusta* (B) using the original re-sequencing data and extra rounds of subsampling to lower sequencing coverages. White numbers refer to absolute number of genes while black numbers indicate fractions.

As depicted in *Figure 2*, SGSGeneLoss yielded highly similar results using the original and sub-sampled sequencing coverages, as the variation in the core gene content goes from ~1% in *P. tricornutum* to ~7% in *S. robusta*. The larger variation in *S. robusta* can most likely be attributed to the more complex genome (e.g. number of repeats and multi-gene families) compared to *P. tricornutum*. The results from our subsampling control experiment are in line with previously reported results (Montenegro J. D. et al., 2017 reported a 0.05% error rate using 10x read coverage) and demonstrate that the *Seminavis robusta* dispensable gene content presented in this study is unlikely to be highly inflated by the 8.26x average coverage data. We also want to point out that the apparently low fraction of 74% core genes in *S. robusta* is in line with *Emiliania huxleyi*, where it was reported that the 'core genome' contains about two-thirds (66%) of the genes predicted in the reference genome (Read, B. A. et al., 2013).

B. Related to newly detected pan genes, we do agree with the reviewer that the low coverage data is suboptimal to have a complete view on variable genes missing in the reference genome. However, it is important to note that we only added *de novo* assembled pan genes if these did not map to the reference genome with sequence identity >= 75% (reported in main Methods of the manuscript). In other words, we would wrongly label missed core genes as new pan genes if these genes showed more than 25% divergence with the reference genome, which is very unlikely for a core gene in the same species.

In conclusion, we have revised/reduced this section, as also requested by reviewer 1, and have included in the main text the concept of softcore genes (*Figure 3*, *Supplementary Figure S15* in the manuscript), which has been used in other pan genome studies (Inglin, R. C., et al. 2018, Gordon, S. P., et al., 2017, Gao L. et al., 2019) to reduce the potential impact of poor quality sequences on the core gene set:

"Assessing the gene content diversity across all 49 S. robusta strains revealed that 28,120 (74%) genes are core while the remaining 9,683 (26%) genes represent the potential dispensable fraction (*Figure 4B*, *Supplementary Data S4*). **From this dispensable fraction, we found 2,551 (6.7%) softcore genes (present in >=95% but not all of the strains), indicating the fraction of core genes probably ranges between 74-81% (*Supplementary Figure S15*)**."

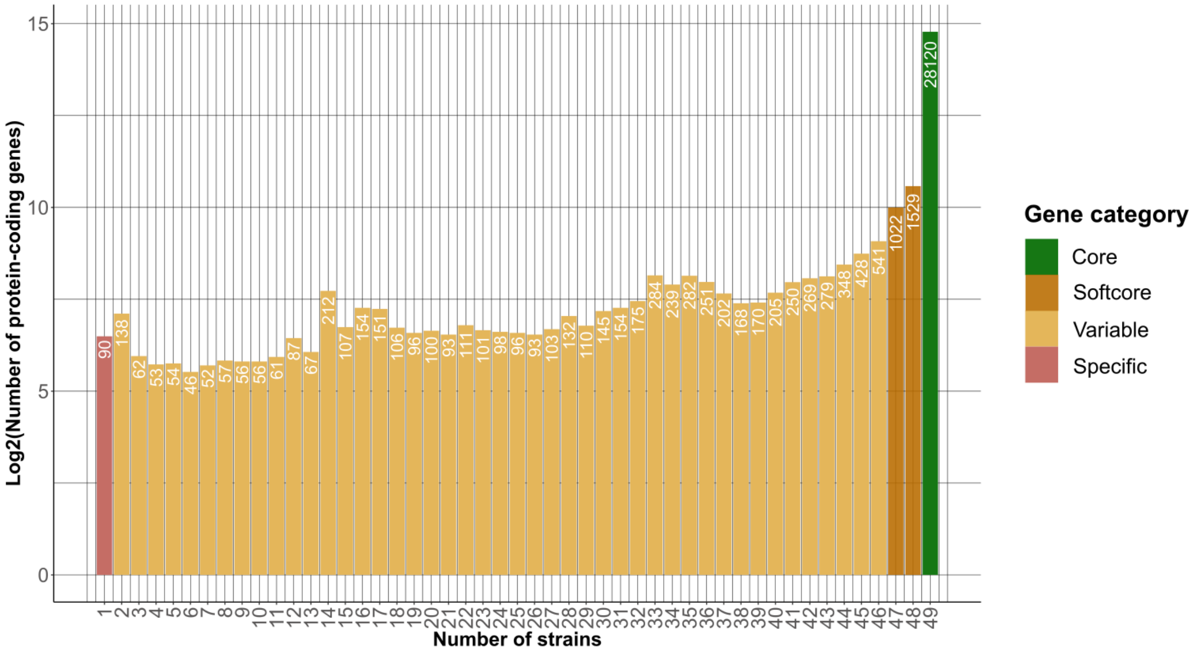

Figure 3. Number of protein-coding genes found in a specific number of strains (1-49), colored by gene category. Core genes are genes present in all strains, softcore genes are genes present in >=95% of the strains, variable genes are genes present in <95% of the strains and specific genes are genes present only in one strain. Dispensable genes encompass these last three categories. White numbers refer to absolute number of genes.

In addition it is written page 11 "many dispensable genes lack functional annotation, the remaining genes are enriched in DNA metabolism, zinc fingers, transposable element-derived

proteins, and telomeric proteins". These annotations suggest potential retentions of repetitive sequences in the gene set, which have to be checked.

With regards to this comment, we would like to refer to our reply to comment 2). In addition, we determined the fraction of repeat-like proteins in the dispensable gene set, finding only 174 genes annotated as potential repeats in the reference genome (1.6% of dispensable reference genes). The main challenge is that of the 151 families significantly enriched in dispensable genes, 122 lack functional annotation. In spite of this high fraction of genes without functional description, 5,125 dispensable reference genes (63%) showed expression (TPM >= 2 in at least one experimental condition), indicating these are truly transcriptionally active genes. We revised the text to clarify this and avoid misinterpretation of these results:

"**In contrast to these, 122/151 families significantly enriched in dispensable genes lack functional annotation. Among the remaining annotated dispensable genes, we find telomeric proteins**, which are known to undergo rapid evolution. In particular, it has been shown that gene duplicates create telomere paralogs with novel functions in telomere replication and chromosome end-protection [31]. The observation that 63% of the *S. robusta* dispensable genes in the reference genome are expressed and several of these encode for transporters and metabolic proteins indicates this genomic variation represents transcriptionally active genes, potentially offering a template for phenotypic or physiological evolution in this diatom."

References:

Golicz, A.A., et al. (2016). The Pangenome of an Agronomically Important Crop Plant Brassica Oleracea. Nat Commun, 7, 13390.

Montenegro J. D. et al. (2017). The Pangenome of Hexaploid Bread Wheat. Plant J, 90 (5), 1007-1013.

Gao L., et al. (2019). The tomato pan-genome uncovers new genes and a rare allele regulating fruit flavor. Nat Genet, 51 (6), 1044-1051.

Golicz, A.A., et al. (2015). Gene loss in the fungal canola pathogen Leptosphaeria maculans. Funct. Integr. Genomics, 15, 189–196.

Rastogi, A., et al. (2019). A genomics approach reveals the global genetic polymorphism, structure, and functional diversity of ten accessions of the marine model diatom *Phaeodactylum tricornutum*. ISME J, 14 (2), 347-363.

Read, B. A. et al. (2013). Pan Genome of the Phytoplankton Emiliania Underpins Its Global Distribution. Nature, 499 (7457), 209-13.

Inglin, R. C., et al. (2018). Clustering of Pan- and Core-genome of Lactobacillus provides Novel Evolutionary Insights for Differentiation. BMC Genomic, 19: 284.

Gordon, S. P., et al. (2017). Extensive Gene Content Variation in the Brachypodium Distachyon Pan-Genome Correlates With Population Structure. Nat Commun, 8 (1), 2184

5) Page 12, an analysis of the purifying and positive selections led to the conclusion that dispensable genes are less constrained. This is also hypothesized for genes of unknown functions. In both cases, the set is enriched for poorly conserved genes, which may be difficult

to annotate, and potentially could create cases of poor alignments interpreted as cases of positive selections. The authors should show more convincingly that this is not the case, testing for biases potentially introduced in this analysis.

This technical concern raised by the reviewer is correct, and it was taken into account during our initial analysis. As explained in more details in *Supplementary Note S6*, we tackled this by performing the πN / πS ratios only in the coding regions of a high-confidence SNP dataset. Specifically, we selected a subset of resequencing strains with callable positions in 80% of the reference coding regions (no multimapping allowed + minimum read depth of 3, as we were only looking at homozygous SNPs) and a subset of protein-coding genes with a sufficient horizontal coverage (i.e. >50% of the gene length was callable). Therefore, although a fraction of genes was indeed discarded in this analysis due to low coverage (15,168/36,254 genes were excluded in this analysis), the reported results are all based on genes with good coverage not suffering from poor alignments.

6) From page 13 onwards, an interesting conservation analysis is made using 88 transcriptomes of very diverse diatoms from the MMETSP. The authors found interesting cases of apparent correlation between gene presence and some traits, and provide explanations like "benthic signatures" or raphid-specific traits. This would be reinforced by a clear comparison on how the traits and gene amplifications are present in the diatom phylogeny. This would be more insightful than some of the present conclusions on rhodopsins and ROS which are expected, and not experimentally shown to be linked to the hypotheses.

We thank the reviewer for this useful suggestion. After extensive discussions with all senior authors on how to implement this, we have created a new figure depicting the diatom species tree (Nakov et al., 2018) together with the occurrence of some raphid-specific and benthic-specific traits described in this study (see *Supplementary Figure S17* in the manuscript, *Figure 4* below). Note that our signature analysis is based on the MMETSP transcriptomes, meaning we cannot accurately address the gene copy number (gene amplifications). We have additionally revised the text:

"**In addition to these pennate signature genes, we also searched for raphid-specific and benthic-specific traits in the diatom phylogeny (*Supplementary Figure S17*), finding proteins widely conserved in most raphid species (e.g. 'peptidase C2, calpain' proteins) and others more restricted to certain benthic species**. In particular, within the 241 genes showing a high raphid signature […]"

"[…] **Apart from *S. robusta*, *Amphora* and *Trybionella* are other diatom genera containing the globin-like and GPCR-rhodopsin-like signature genes (*Supplementary Figure S17*), confirming that these traits are present in different clades of benthic diatoms.**"

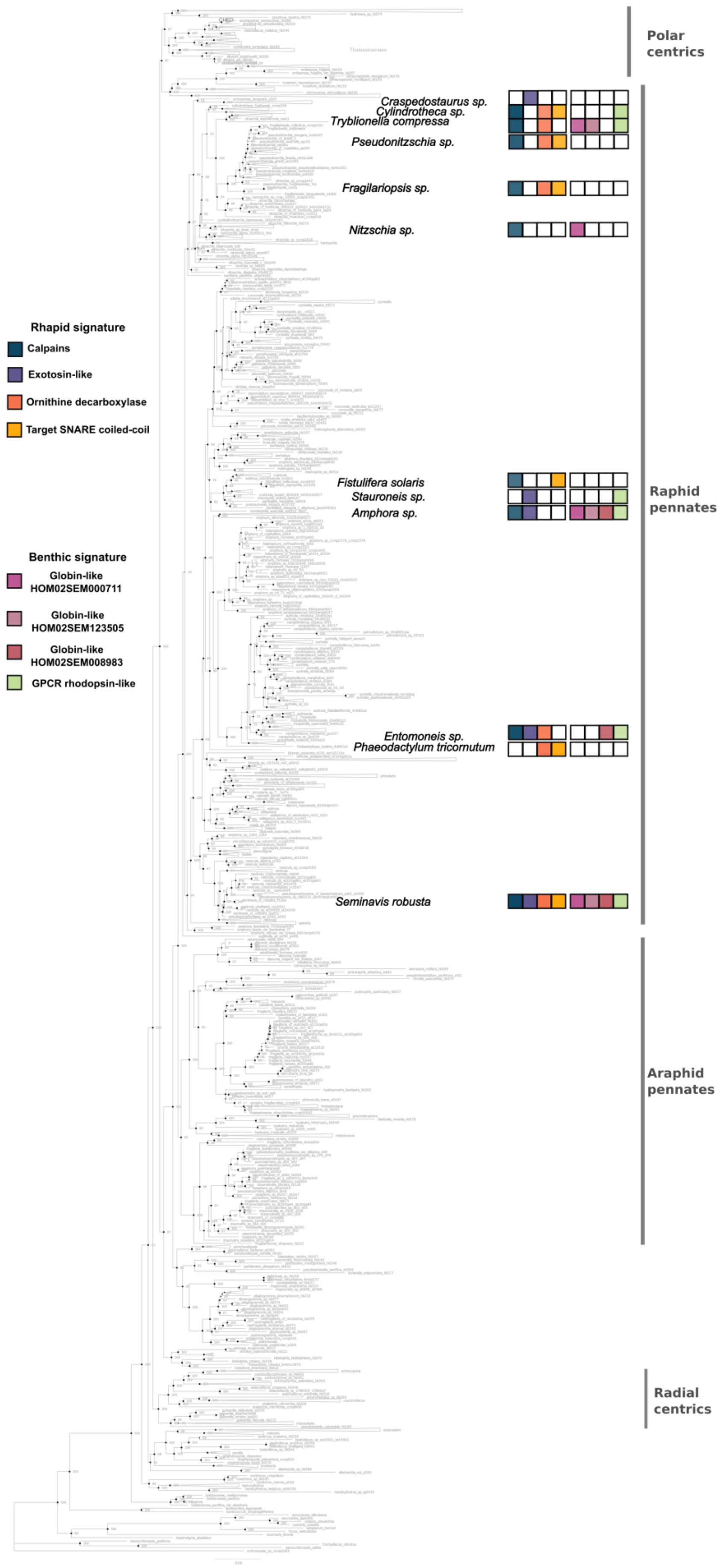

Figure 4. Diatom species tree showing the presence/absence in the diatom phylogeny of some of the raphid-specific and benthic-specific traits described in this study.

Reference:

Nakov et al., (2018). Accelerated Diversification Is Related to Life History and Locomotion in a Hyperdiverse Lineage of Microbial Eukaryotes (Diatoms, *Bacillariophyta*). New Phytology. 219 (1), 462-473.

Minor point

7) Page 5, the diatoms with larger genomes are shown to have more gene families than those with smaller genomes. Is this conclusion robust with the way the heterozygosity was treated in the other studies?

To address this comment, we have determined the genome-wide collinearity within all diatom species (i.e. intra-species synteny) and counted the number of gene pairs in duplicated regions (anchorpoints) with a Ks < 0.1, as these are potential candidates to contain haplotype redundancy due to heterozygosity (see *Figure 5*).

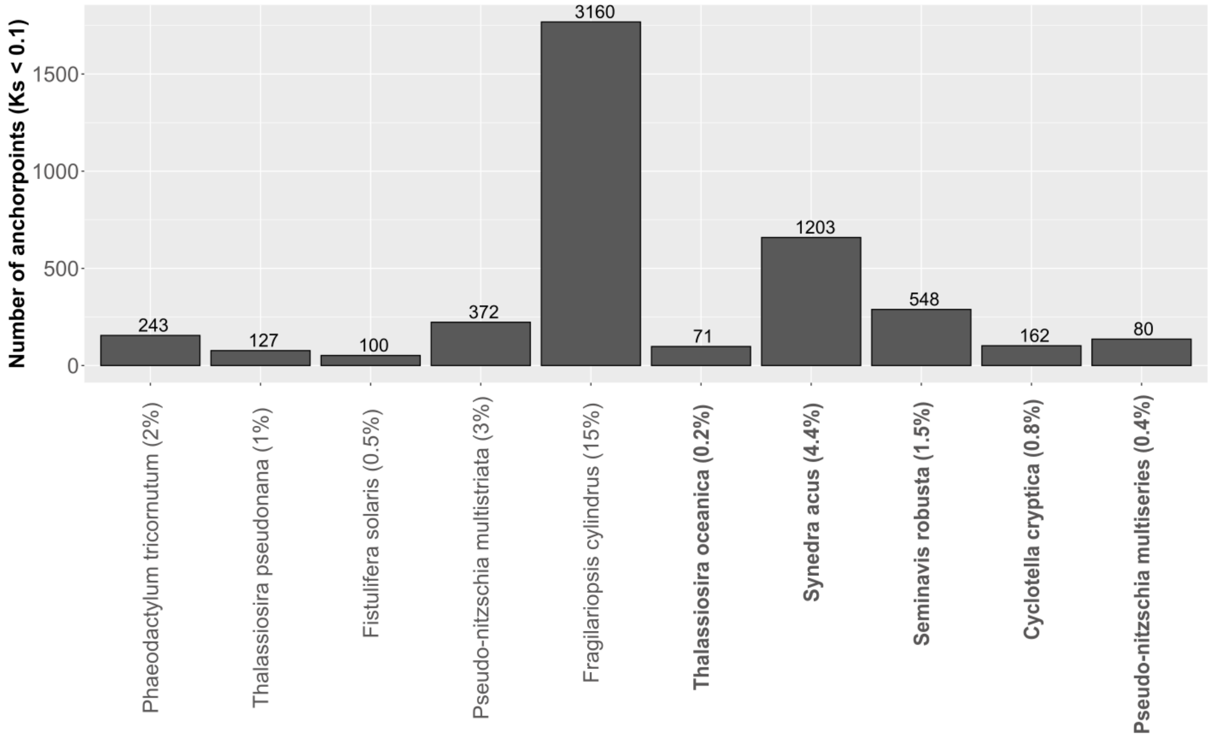

Figure 5. Number of anchorpoints with Ks < 0.1 for all diatom species. Black numbers refer to the absolute number of genes belonging to the anchorpoints. Species are sorted by genome size in the x-axis while species with genome size >90 Mb are in bold. The percentage next to the species names refers to the total fraction of genes that might be suffering from heterozygosity.

Apart from *F. cylindrus*, where 3,160 genes might be different alleles rather than distinct gene copies (15% of all genes, 3,160/21,063 genes), the values for all diatoms with large genomes (in bold) are all below 5%, indicating that heterozygosity is not a major factor affecting gene counts and gene family sizes.

Reviewer #3 (Remarks to the Author):

The manuscript by Osuna-Cruz et al. ostensibly presents the genome of the benthic diatom Seminavis robusta. However, the manuscript goes well beyond a genome "announcement" and includes results from re-sequencing of 48 S. robusta strains from three clades to identify core genes and strain-specific genes, analysis of dozens of transcriptomes to enhance gene-model predictions and identify gene families induced under specific or multiple conditions, and incorporates comparative analyses (including the open Diatom Plaza) to identify genes essential to life in a benthic environment. This is a stunning body of work that raises the bar very high for other genome papers and will undoubtedly be emulated by others. Therefore, I provide here some detailed suggestions to ensure that others can reproduce the results and analyses. I also suggest that the authors discuss their results in a bit more detail and provide more comparisons to what is known from other organisms. The discussion of the purifying selection is a good example of comparative discussion that should be enhanced in some other sections.

We thank the reviewer for the positive and constructive comments. As reported in our response to reviewer 2, comment 6, we have added a new figure discussing some of the reported signature genes/traits in the context of the diatom phylogeny.

1) Figure 2. Panel A. The authors should not assume that the reader will know what an "upset plot" is and should provide more details. For example, what are the black vs gray circles in the rows and what do the vertical lines connote?

We agree with the reviewer and revised the text of the legend in Figure 2A.

"(A) Upset plot showing the intersection of gene family expansions in diatoms. Each row represents a diatom species with in parenthesis reporting the total number of expanded gene families. **Black circles and vertical lines between the rows represent the intersection of expanded families between species.** The barplot indicates the total gene family count in each intersection, displaying only intersections that contain at least ten gene families. Diatoms with a genome size > 90 Mb are highlighted in bold."

2) Figure 3. Panel A. Axis label descriptions in legend are reversed. Panel D. This panel uses a lot of symbols/colors. Please make sure all are described. For example, what do the colored boxes indicate? The color legend in the center of the figure seems to relate to panels B, C and D, but this is hard to understand.

We agree with the reviewer and revised Figure 3 and the text of its legend. We removed the age categories from Figure 3B to simplify the figure since these are not discussed anywhere in the text, and moved the color legend accordantly to improve the clarity. Below are highlighted (in bold) changes in the legend:

"(A) Expression divergence trend for multi-copy *S. robusta* families. The **y-axis** denotes the percentage of nodes showing expression divergence in the phylogenetic tree of the family, while the **x-axis** represents the number of *S. robusta* gene copies in the family. Average expression divergence percentages are indicated by red dots. Median expression divergence values significantly higher than the median of all nodes are highlighted with a star (pvalue<0.05, Wilcoxon rank sum test).

[…]

(C) Barplot showing family counts with significant condition-specific expression. The x-axis represents the different conditions/experiments whereas the y-axis represents the number of families having significant expression bias for that condition. **The color of the bars denotes the family age distribution.**

(D) Network showing families with significant specific expression in the three reproduction stages available. Families are represented with circles while conditions are represented with diamonds. **The color of the circles denotes the family age following the same color code as panel C.** The edge's width denotes the fraction of genes in the family that shows upregulation for the given condition, **while the edge's color represents the significance of the enrichment, following the same color code as panel B from grey to dark purple. Expansion and tandem enrichment of each family are indicated by the squares next to the gene family circles, also following the same color code as panel B.** Numbers in superscript refer to families annotated in *Supplementary Data S1*."

3) Figure 5. The experimental design was not entirely clear. It was not immediately clear to me that the MMETSP data was used only to define the different categories of genes that would be analyzed in the Seminavis transcriptomes and that the up/down arrows were derived from these latter analyses.

We agree with the reviewer and revised the text on this result's section:

"The Marine Microbial Eukaryote Transcriptome Sequencing Project (MMETSP) database [37] was used as a reference dataset to find *S. robusta* signature genes that might be implicated in specific morphological or ecological properties. These genes were identified by first determining, for each *S. robusta* gene, homologs in a set of 88 diatoms and subsequently selecting those genes predominantly present in pennate, raphid and benthic species, compared to centric, araphid and planktonic species, respectively (see Material and methods and *Supplementary Data S5-6*). **The differential expression of these *S. robusta* signature genes was further inspected to give more insight about the biological roles of these genes.**"

We also revised the text in the legend of *Figure 5*:

"Figure 5: Selection of signature genes showing strong pennate (A), raphid (B) or benthic (C) conservation. Significant enrichment for differential expression **in the *S. robusta* transcriptome** of genes showing the specified signature are highlighted with downward (for downregulation) or upward (for upregulation) arrows colored by experiment."

4) The authors provide citations for all the software used in their analyses. Please also include all settings used to allow others to reproduce the results.

For each analysis, we have revised the text in both main Methods and Supplementary Information to ensure we include all necessary tools and parameters:

- DNA extraction, library preparation and genome size estimation: details about DNA isolation techniques and flow cytometry are given now in *Supplementary Note S1*. Details about genome size estimation based on k-mer distribution using GenomeScope are found in *Supplementary Figure S1*. For DNA read preprocessing, tools and parameters are mentioned in the footnotes of *Supplementary Table S1*.

- Genome assembly and repeat identification: detailed parameters used to run different assembly tools (including Platanus and PBJelly) are mentioned in the footnotes of *Supplementary Table S2*. Detailed parameters used to run RepeatModeler and RepeatMasker are found in the footnotes of *Supplementary Table S3*. Assembly polishing and quality measures' tools and parameters are described in *Supplementary Note S2*.

- Gene prediction and functional annotation: *Supplementary Note S2* describes tools and parameters used to assess the gene model's quality.

- *S. robusta* comprehensive expression atlas and gene differential expression analysis: detailed information about the experimental conditions and pre-processing of the RNA-Seq reads to build the *S. robusta* gene expression atlas are given in *Supplementary Table S4*.

- Comparative genomics analysis: a detailed list of tools and parameters used to build PLAZA Diatoms 1.0 is reported in *Supplementary Table S7*.

- Identification of core and dispensable *S. robusta* protein-coding genes: all information is given in the main Methods.

- SNP calling and analysis of gene selection patterns: detailed information about the tools and parameters used to generate a high confidence SNP dataset are given in *Supplementary Note S6*.

- Identification of *S. robusta* genes with high pennate/raphid/benthic signature: all information is given in the main Methods.

5) The authors generate additional transcriptomes for this manuscript. They need to provide additional details about the growth and harvest conditions.

Experimental conditions for the RNA-Seq data generation are described in *Supplementary Table S4* as the length of the methods section is limited. In this table, we give information about the control and treatment procedures for each condition, as well as the number of replicates, strains used, duration of the treatment and read length. In the caption of the table, we provide more details about growth conditions, RNA extraction, and sequencing.

6) The authors should provide details about the flow cytometry methods used to estimate genome size and possible explanations for the discrepancy in size estimates.

We have added more detailed information about the flow cytometry methods in *Supplementary Note S1*. In short, the genome size was estimated repeatedly (n = 28) comparative to two other diatom species with known genome size (*P. tricornutum* and *F. cylindrus*). Please note the estimates obtained in this way were rather variable (standard deviation 17.6 Mb, mean of 151 Mb), which may explain the discrepancy with the other size estimates. To underline the uncertainty of this method, we added the standard deviation in the main Results:

"A k-mer distribution analysis of the Illumina reads revealed a high level of heterozygosity (0.79%) and an estimated genome size of 117 Mb (*Supplementary Figure S1*), while flow cytometry yielded an estimate of **151 Mb (standard deviation 17.65 Mb, *Supplementary Note S1*)**."

7) The authors should provide more details on the manual checking used to remove only the 4 scaffolds out of 33 scaffolds that were designated as potential bacterial contamination.

We revised the legend of the *Supplementary Figure S2* and added more details:

"[…] These scaffolds were manually inspected and only 4 scaffolds were removed from the assembly as true contaminants **since these contain genes without strong expression evidence and coming from the same contaminant species (e.g. *Escherichia coli*).**"

In addition to this, we have performed an extra control experiment to assess the contamination of *Seminavis robusta*'s scaffolds based on a BLASTN search against the complete non-redundant nucleotide database (date 01/10/19). Scaffolds that had an identity >70% and alignment coverage >25% with non-stramenopiles organisms were deemed to be potential contaminants. Apart from the scaffolds already removed, this new analysis did not identify any additional scaffolds as contaminants. These results confirm that the *Seminavis robusta* genome is not suffering from strong contamination.

"*Supplementary Figure S2*, legend: **Secondly, a BLASTN sequence similarity search (identity >70% and coverage >25% with non-stramenopiles organisms) against the NCBI nucleotide database was performed to identify scaffolds that could still represent potential contamination. Apart from the scaffolds already removed, this extra analysis did not identify any additional scaffold as true contaminant.**"

8) Figure S3. This figure/legend needs more detail to be interpretable. What are the different lanes for each labeled contig? Although expected product sizes are provided in Table S8, it would be helpful to include the expected product size in this figure.

We added the expected produced size to the figure and revised the legend of the *Supplementary Figure S3*:

"Supplementary Figure S3: Gels from the experimental validation of the *Seminavis robusta* genome assembly. From panel A–D, twenty-two regions amplified by PCR are shown. **There are three lanes from different PCR runs for each amplified region, reporting the expected product size on the upper part. More details about these amplified regions, their coordinates, primers and exact product size obtained are given in *Supplementary Note S2* and *Supplementary Table S9*.**"

9) Detection of reference-specific genes deserves more discussion. What is the likelihood that these genes are truly strain-specific? What sequencing depth of the re-sequenced strains would be required to address this question?

As shown in *Figure 2* of our reply to comment 4 of reviewer 2, the SGSGeneLoss methodology to determine presence/absence gene variation is stable for different sequencing depths. However, since we only detected 90 potentially specific genes (of which 82 are reference specific), and following the advice of reviewer #1 and reviewer #2 to reduce this section to improve the clarity and the focus of this manuscript, we decided to remove these results from the main text.

10) The sentence on page 10 beginning with "The six times higher fraction of dispensable genes…" requires a reference citation.

As explained in the methods, we used the resequencing data of *Phaeodactylum tricornutum* (Rastogi, A., et al., 2019) and reprocessed the raw data to also address the gene presence/absence variation in this species. We revised the text to also cite this reference in the results and not only in the methods:

"However, determining the size of the dispensable gene content using re-sequencing data of ten geographically distant accessions in *P. tricornutum* **(Rastogi, A., et al., 2019)** revealed that only 4.46% of the genes are absent in at least one accession (558/12,517 core genes, 172 de novo-assembled genes). The six times higher fraction of dispensable genes in S. robusta suggests much more de novo gene evolution in this benthic diatom compared to P. tricornutum, although convergent gene loss due to long-term culturing for the latter species might provide an alternative explanation."

<u>Reference:</u>

Rastogi, A., et al. (2019). A genomics approach reveals the global genetic polymorphism, structure, and functional diversity of ten accessions of the marine model diatom *Phaeodactylum tricornutum*. ISME J, 14 (2), 347-363.

11) Also, it is not clear whether the sentence on page 15 beginning with "Expression analysis of the GPCR rhodopsin-like protein ortholog…" is part of this study or is a reference to other work. Please clarify.

We performed a differential expression analysis using the data from Valle, K. C., et al. 2014 under different light conditions. This is explained in more details in *Supplementary Figure S20B*, but we revised the main text to clarify this:

"**A differential expression analysis under different light conditions (Valle K. C. et al. 2014) of the GPCR rhodopsin-like protein ortholog in *P. tricornutum* shows that** this gene is specifically upregulated under blue-green light exposure (*Supplementary Figure S21B*), confirming our hypothesis this family plays an important role in light sensing."

<u>Reference:</u>

Valle, K. C., et al. (2014). System Responses to Equal Doses of Photosynthetically Usable Radiation of Blue, Green, and Red Light in the Marine Diatom Phaeodactylum Tricornutum. PLoS One, 9 (12), e114211

12) Please explain how reference strain chosen and add specificity to DNA isolation techniques. Clarify if this strain was used for transcriptome studies and if not used (as it appears), please explain why.

We have added more detailed information about the DNA isolation techniques in *Supplementary Note S1*. Strain D6 from *S. robusta* clade 1 (MT+) was chosen as a reference strain for its fast growth and ease of use in the laboratory. The size of the strain had become quite small by the time the transcriptomic experiments were set up, so other strains were used for later experiments. For most new transcriptomic experiments, we adopted 85A, a clade 1 (MT+) strain that is closely related to D6 in the *S. robusta* pedigree: they share a parent and all parental strains are the result of a single cross (*Figure 6*). All strains used for each transcriptomics experiment are shown in *Supplementary Table S4* of our manuscript. Read

mapping statistics revealed that the usage of these closely related strains did not interfere with downstream analyses.

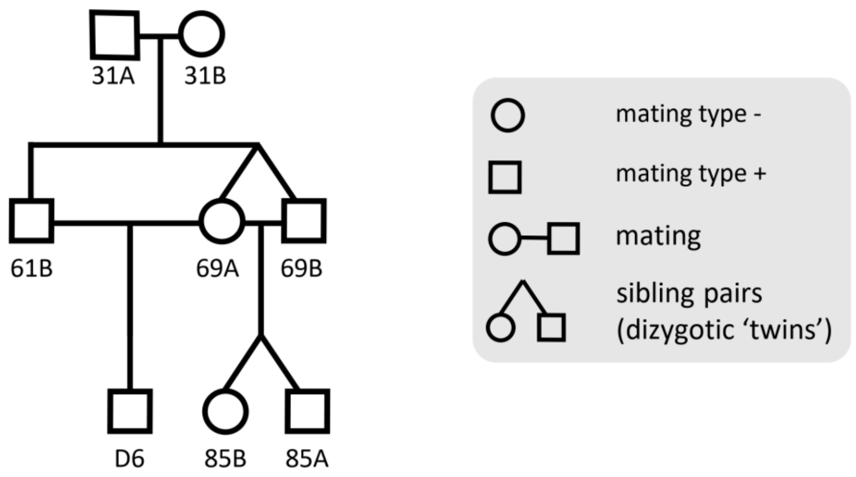

Figure 6: Simplified pedigree showing the relationship between strain D6 (reference strain of genome assembly) and strain 85A (MT+ strain used for most new transcriptomic experiments).

13) Please note that diatoms have metacaspases, not caspases (page 11). Please correct.

We agree with the reviewer and revised the text:

"These families are related to essential functions (e.g. light-harvesting involved in photosynthesis, **metacaspases** involved in proteolysis, cyclins controlling cell cycle regulation) but also encompass tandem-enriched expansions involved in signaling, including HSF, cGMP and LRR related proteins."

14) Page 4. The authors state "Newly generated expression data combined with existing data…" Please provide references for existing data.

References to existing transcriptome data, as well as information on experimental conditions for these samples, are given in *Supplementary Table S4*. We revised the text to indicate this:

"Newly generated expression data combined with existing data (***Supplementary Table S4***) were used to create a large-scale gene expression atlas profiling 31 different experimental conditions."

REVIEWERS' COMMENTS:

Reviewer #2 (Remarks to the Author):

My concerns were adequately addressed with this revision. Regarding the quality of the assembly, I would suggest that the limitations pointed by the authors should be detailed in the Methods section. There are probably ways to improve HMW DNA extraction for diatoms (and to perform HiC), so writing about the problems explicitely may help in the future.

Reviewer #3 (Remarks to the Author):

I have reviewed the revised version of the manuscript and am satisfied by the author modifications in response to my original review.

Response letter for reviewers NCOMMS-20-04487B - The *Seminavis robusta* genome provides insights into the evolutionary adaptations of benthic diatoms

Reviewer #2 (Remarks to the Author):

My concerns were adequately addressed with this revision. Regarding the quality of the assembly, I would suggest that the limitations pointed by the authors should be detailed in the Methods section. There are probably ways to improve HMW DNA extraction for diatoms (and to perform HiC), so writing about the problems explicitly may help in the future.

We would like to thanks to the reviewer for all its constructive comments. We have added some sentences in the Method section "DNA extraction, library preparation and genome size estimation" (see below in green) to indicate the challenges of HMW DNA extraction for diatoms in order to address the last comment of reviewer.

"Both Illumina and PacBio technologies were used for sequencing of *S. robusta* D6 strain mating type plus (MT+) to take advantage of short-read (better quality) and long-read (better contiguity) sequencing approaches. Diatoms have silicified cell walls, which means that the high molecular weight (HMW) DNA extraction is not trivial. Harsh conditions are needed to break the wall, which also affects the integrity of the chromosomal DNA. Our final assembly (see next section) is based on a hybrid dataset combining 18 million Illumina paired-end reads as well as >1 million PacBio long reads (*Supplementary Table 1*). In particular, Illumina DNA was extracted using a DNeasy Plant Mini Kit (Qiagen), paired-end libraries were prepared with an insert size of 500-800 bp and sequencing was performed on a 2x 300bp Miseq system. In the case of PacBio, different HMW DNA extraction protocols were tested, Blue Pippin size selection was applied to enrich for long fragments, libraries were prepared with an insert size of 10,000 bp, and sequencing was performed in two different locations: 10 SMRT cells were sequenced on the RSII at VIB Nucleomics Core (Leuven, Belgium) whereas an extra 1 SMRT cell was sequenced on the RS at GATC Biotech AG (Konstanz, Germany). In all scenarios the obtained average read length was around 5kb (*Supplementary Table 1*), which is lower compared to standard PacBio read lengths applied on other non-diatom species. A comparison with the *Fragilariopsis cylindrus* genome [12] where the average PacBio read length was 2.4kb reveals our long reads are of good quality. More details about DNA isolation methods and genome size estimation by flow cytometry are found in *Supplementary Note 1.* Additionally, k-mer distribution statistical approach was used to estimate the genome size, repeat content and heterozygosity (*Supplementary Figure 1*)."

Reviewer #3 (Remarks to the Author):

I have reviewed the revised version of the manuscript and am satisfied by the author modifications in response to my original review.

We would like to thanks also to the reviewer for all its constructive comments and we are glad that he/she is satisfied with our revised version.
